# Synthesis, Antiparasitic Activity and Substituent Effects of Methyl 5-(Hetero)aryl or Alicyclicaminothieno[2,3-*b*]pyridine-2-carboxylates

**DOI:** 10.3390/molecules31081313

**Published:** 2026-04-17

**Authors:** Francisco Ribeiro, Juliana P. Sousa, Nuno Santarém, Joana Tavares, Anabela Cordeiro-da-Silva, Maria-João R. P. Queiroz

**Affiliations:** 1 Centro de Química, Universidade do Minho (CQUM), Campus de Gualtar, 4710-057 Braga, Portugal; b15328@uminho.pt (F.R.); pg52275@uminho.pt (J.P.S.); 2 Host-Parasite Interactions, I3S—Instituto de Investigação e Inovação em Saúde, Universidade do Porto, Rua Alfredo Allen, 208, 4200-135 Porto, Portugal or nsantarem@ff.up.pt (N.S.); or jtavares@icbas.up.pt (J.T.); or cordeiro@ff.up.pt (A.C.-d.-S.); 3 Laboratório de Microbiologia, Departamento de Ciências Biológicas, Faculdade de Farmácia, Universidade do Porto, Rua de Jorge Viterbo Ferreira, 228, 4050-313 Porto, Portugal; 4 Departmento de Biologia Molecular, ICBAS—Instituto de Ciências Biomédicas Abel Salazar, Universidade do Porto, Rua de Jorge Viterbo Ferreira, 228, 4050-313 Porto, Portugal

**Keywords:** Buchwald–Hartwig amination, di(hetero)aryl or alicyclicamines, homogeneous catalysis, thieno[2,3-*b*]pyridines, *T. brucei*, *L. infantum*, substituent effects

## Abstract

Di(hetero) aryl and alicyclic amine derivatives of thieno[2,3-*b*]pyridine were synthesized in good to high yields (45–76%) via palladium-catalyzed Buchwald–Hartwig amination. The reactions were performed using methyl 5-bromothieno[2,3-*b*]pyridine-2-carboxylate, prepared in this work, and a variety of substituted anilines bearing either electron-donating groups (EDGs) or electron-withdrawing groups (EWGs), as well as pyridinyl amines, and saturated heterocyclic amines such as morpholine and piperidine. For most substrates, the optimal conditions involved Pd(OAc)_2_, *rac*-BINAP, and Cs_2_CO_3_ in toluene at 100 °C under argon. Substrate bearing EWGs and electron-deficient pyridinyl amines required Xantphos as the ligand, while reactions with piperidine were only successful using Pd_2_(dba)_3_ as a palladium (0) source. The antiparasitic activity of the synthesized compounds was evaluated against *Trypanosoma brucei* (*T. brucei*) and *Leishmania infantum* (*L. infantum*) in both promastigote and amastigote forms. Most compounds exhibited no significant cytotoxicity (CC_50_ > 100 μM) in PMA-differentiated THP-1 derived macrophage cells. Analysis of substituent effects focusing on the nature of amino substitution at position C(5) revealed distinct trends in antiparasitic activity. Notably, one compound exhibited activity against *Leishmania infantum* promastigotes that was nearly four times higher than that of the reference drug miltefosine, and its selectivity index was also approximately fourfold higher.

## 1. Introduction

The palladium-catalyzed amination of (hetero)aryl halides has become an important method for the synthesis of di(hetero)arylamines over the past 25 years [1]. This reaction has contributed to the development of pharmaceuticals, agrochemicals, and organic materials. Improvements in catalyst and ligand design have expanded the scope of this transformation, enabling its application in both academic and industrial research. The scope of the reaction is wide and includes aryl and heteroaryl halides as well as various amine partners, such as primary and secondary alkylamines, anilines, and nitrogen-containing heterocycles (e.g., pyrroles and pyrazoles). As a result, the development of efficient and general methods for C-N bond formation in di(hetero)arylamines remains highly relevant, particularly in the context of medicinal chemistry. Aromatic and heteroaromatic scaffolds are of particular interest because of their biological and physicochemical properties [2,3,4].

Vector-borne parasitic diseases remain a major global health challenge, particularly in tropical and subtropical regions. *Trypanosoma brucei*, the agent of human African trypanosomiasis, and *L. infantum*, responsible for visceral leishmaniasis, are transmitted via infected insect vectors and exhibit complex life cycles with pronounced morphological and biochemical adaptations. Current treatments are limited by toxicity, high cost, prolonged regimens, and emerging resistance, highlighting the urgent need for new, safe, and effective antiparasitic agents [5].

Thieno[2,3-*b*]pyridines’ derivatives have gained importance due to their reported anti-inflammatory [6], antiviral [7] and antiparasitic [8,9] activities.

**Figure 1:**
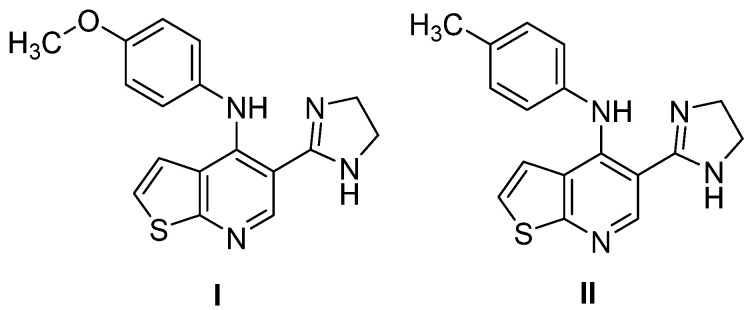
The 5-(4,5-dihydro-1*H*-imidazol-2-yl)-4-(arylamino)thieno[2,3-*b*]pyridine derivatives (**I**, **II**) with anti-*L. amazonensis* activity [8].

Castro et al. reported, in 2012 [8], the synthesis and antiparasitic evaluation of a new series of 5-(4,5-dihydro-1*H*-imidazol-2-yl)-4-(arylamino)thieno[2,3-*b*]pyridine derivatives that showed antileishmanial activity. Among these compounds, the most promising activity against *L.amazonensis* was observed for compounds **I** and **II**, which bear a *p*-methoxy substituent on the phenyl ring, and for the derivative containing a *p*-methyl substituent, respectively (Figure 1). The analysis of the concentration required to inhibit 50% of *L. amazonensis* growth after 24 h (EC_50_) revealed that compounds **I** and **II** (EC_50_ = 29.49 and 32.23 µM, respectively) exhibited a better antileishmanial profile than Glucantime (EC_50_ = 163.7 µM), a standard drug used in the treatment of leishmaniasis.

**Figure 2:**
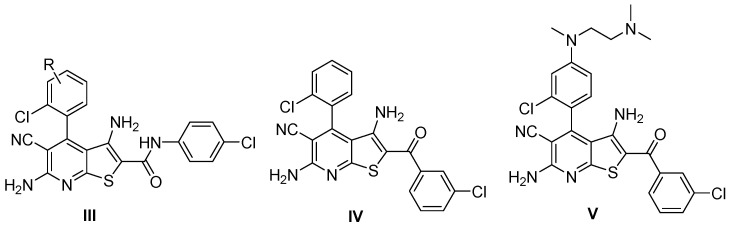
Thieno[2,3-*b*]pyridines with antiplasmodial activity (**III**, **IV**, **V**) [9,10].

In 2020, Kunick et al. reported the synthesis of a series of thieno[2,3-*b*]pyridine-2-carboxamide derivatives (**III**), representing a novel class of compounds with potent antiplasmodial activity [9]. These derivatives effectively inhibited the proliferation of erythrocytic forms of the parasite at very low concentrations, with half-maximal inhibitory concentrations (IC_50_) in the nanomolar range. Among the synthesized compounds, derivative **III** (R = H) exhibited very high antiplasmodial activity (IC_50_ = 199 nM) compared to the lead ketones **IV** and **V** (Figure 2). Compounds **III** were initially designed based on ketones **IV** and **V**, which are selective inhibitors of plasmodial glycogen synthase kinase-3 (PfGSK-3), with IC_50_ values for PfGSK-3 enzyme inhibition of 0.48 μM and 1.20 μM [10], respectively. Interestingly, while compound **III** (R = H) does not inhibit PfGSK-3, it suppresses the proliferation of erythrocytic parasite forms far more effectively than ketones **IV** and **V**. Cytotoxicity assays using HEK293T cells indicated that compound **III** is not toxic.

Herein, we report the synthesis of twenty-one new methyl 5-[(hetero)aryl- or alicyclicamino]thieno[2,3-*b*]pyridine-2-carboxylates. These compounds were obtained via a Pd-catalyzed Buchwald-Hartwig amination of methyl 5-bromothieno[2,3-*b*]pyridine-2-carboxylate, also prepared in this work, with aniline derivatives, (hetero)arylamines, and saturated heterocyclic amines. The resulting compounds were evaluated against *T. brucei* and *L. infantum*, and the effects of substituents were studied. The structural modification of the thieno[2,3-*b*]pyridine scaffold was intentionally restricted to the C(5) position in order to systematically investigate the influence of substituents at this position on biological activity while maintaining the integrity of the core pharmacophore. The cytotoxicity was evaluated in phorbol 12-myristate 13-acetate (PMA)-differentiated THP-1-derived macrophages.

## 2. Results and Discussion

### 2.1. Synthesis of Methyl 5-[(Hetero)aryl or Alicyclicamino]thieno[2,3-b]pyridine-2-carboxylates ***2a**–**2u***

After the preparation of methyl 5-bromothieno[2,3-*b*]pyridine-2-carboxylate (**1**) (see the Supplementary Materials), it was subsequently reacted with various aniline derivatives, heteroarylamines, and heteroalicyclic amines. The reaction conditions were selected based on previous experience of our research group [11] and reports by other authors [12] for Pd-catalyzed C-N Buchwald-Hartwig amination reactions, employing either Xantphos or *rac*-BINAP as ligands, 1,4-dioxane or toluene as solvents, and Cs_2_CO_3_ as the base (Table 1). The desired products were obtained in good to high yields (45–76%).

Analysis of Table 1 indicates that the nature and position of phenyl substituents or heterocyclic rings, as well as the choice of ligands and/or solvents, influenced the product yields. Compound **2a**, bearing an unsubstituted phenyl ring, was obtained in 61% yield under (i**bc**) conditions. *Ortho*- or *para*-electron-donating groups (EDGs) on the phenyl ring, which enhance amino nucleophilicity, generally led to higher yields, as illustrated by **2b** (*o*-OMe, 75%) and **2e** (*o*,*p*-OMe, 65%) derivatives. In contrast, *meta*-EDGs, such as in **2c** *(m*-OMe, 50%) and **2l** (*o*-Cl, *m*-OMe, 45%), resulted in comparatively lower yields under the same conditions.

The mono- and dimethylated compounds **2i**–**2k** were obtained in 60–65% yield under (i**bc**) conditions.

The Xantphos ligand, commonly employed for less reactive or deactivated amines [12], proved to be effective under (i**ac**) conditions, where anilines bearing the weak electron-withdrawing fluorine substituent afforded compounds **2n**−**o** in 50–60%.

Compound **2p** (*m*-CN), obtained under (i**ac**) conditions, was isolated in 50% yield, due to the presence of the electron-withdrawing (EWG) cyano group, which reduces the nucleophilicity of the corresponding aniline. In contrast, compound **2q** (*p*-CN) was isolated in a higher yield (76%) under the same conditions, which may be attributed to its precipitation from the reaction mixture, allowing purification by simple washing with ether rather than by column chromatography.

When amines were incorporated into an electron-deficient pyridine ring under (i**ad**) conditions, the 2-pyridyl derivative **2r** was obtained in only 50% yield due to the inductive electron-withdrawing effect of the pyridine nitrogen, which reduces the nucleophilicity of the amino group. In contrast, the 3-pyridyl derivative **2s** was obtained in a higher yield (70%) under the same conditions, as the pyridine nitrogen is more distant from the amino group, resulting in a reduced inductive electron-withdrawing effect.

For the heteroalicyclic amine derivatives, distinct reactivity patterns were observed. Compound **2t**, derived from morpholine, was formed only when *rac*-BINAP was used instead of Xantphos in toluene. In the case of compound **2u**, obtained from piperidine, Pd(OAc)_2_ in toluene proved ineffective with either ligand. Product formation was achieved only when a Pd(0) catalyst, [Pd_2_(dba)_3_ (tris(dibenzylideneacetone)dipalladium(0))], was employed in combination with Xantphos in dioxane, affording **2u** in 50% yield. Under (i**a**) conditions, piperidine, as a strongly electron-rich Lewis base [13], can coordinate two molecules simultaneously to the metal center, forming a stable bis(amine) palladium complex that suppresses the catalytic cycle by maintaining palladium in an unreactive state [14]. This behavior likely explains the need to employ a Pd(0) species from the beginning rather than generating it *in situ* from Pd(II). The presence of an oxygen atom in position 4 in morpholine exerts an inductive electron-withdrawing effect, slightly reducing the amine Lewis basicity but maintaining the reactivity, while significantly reducing the “poisoning” of the metal catalyst in the process.

### 2.2. Cell Growth Inhibitory Effect of Compounds ***2a**–**u*** on T. brucei and L. infantum Promastigotes and Amastigotes and PMA-Differentiated THP-1-Derived Macrophages Cell Line as a Toxicity Model

The antiparasitic activity of the synthesized compounds was evaluated against *T. brucei* bloodstream forms and *L. infantum*, including both promastigote (extracellular) and amastigote (intracellular) forms. *L. infantum* amastigotes, the intracellular stage residing within human host macrophages, represent the clinically relevant form responsible for disease manifestation. Evaluating drug activity against this stage is critical, as compounds effective only against promastigotes may not reach or act on intracellular parasites. Screening in promastigotes, despite being a simplified model, enables the early identification of compounds capable of affecting *Leishmania* viability under more accessible and controlled conditions. Such molecules might otherwise be filtered out in complex intracellular assays, where factors like host-cell uptake, compartmentalization, or macrophage metabolism can mask intrinsic antiparasitic activity. Thus, testing agents across both life stages provides a more layered perception of the antiparasitic potential of the molecules.

All compounds were screened at single concentrations of 20 μM and 10 μM, and only at 10 μM for *L. infantum* amastigotes. The IC_50_ was calculated for the most active compounds, with activity more than 30% at 10 μM, and more than 50% at 20 μM (see the Supplementary Materials).

A preliminary cytotoxicity assessment of the compounds was performed using PMA-differentiated THP-1-derived macrophage cell line as a toxicity model to determine CC_50_ value, defined as the concentration required to reduce cell viability by 50%. The selectivity index (SI, CC_50_/IC_50_) provides a measure of compound selectivity for the parasite over host cells. Generally, an SI greater than 10 is considered to be promising, indicating high parasite specificity and low host toxicity, whereas values below 5 suggest limited selectivity and increased risk to host cells.

The antiparasitic activity, cytotoxicity, and predicted selectivity index (SI) are presented in Table 2.

Analysis of Table 2 shows that compounds **2f** (*m*,*p*-diOMe) and **2g** (*m*,*m*-diOMe) are the most potent compounds against *T. brucei* with IC_50_ values of 9.66 and 7.88 µM, respectively, and SI > 10. Importantly, most compounds did not exhibit significant cytotoxicity (CC_50_ > 100 μM). The only exception was compound **2l** (*o*-Cl, *m*-OMe), which showed increased cytotoxicity (CC_50_ > 50 µM) and reduced antitrypanosomal activity compared with **2c** (*m*-OMe)**.** The presence of the *ortho*-chloro substituent may introduce steric hindrance or alter molecular conformation, negatively impacting both potency and selectivity. For *L. infantum* promastigotes compounds **2h** (*m*-Me), **2i** (*p*-Me), **2j** (*o*,*p*-diMe), and **2k** (*m*,*m*-diMe), **2p** (*m*-CN) and **2q** (*p*-CN) presented IC_50_ < 10 μM. The best compound is **2q,** presenting a SI > 40. For *L. infantum* amastigotes, none of the compounds was active.

The diversity of synthesized compounds enabled the evaluation of substituent effects on antiparasitic activity.

For *T. brucei*, compounds bearing EDGs were generally more active. Substituent position also influenced activity, with *meta*-substitution being particularly favorable. This is illustrated by the higher potency of compound **2c** (*m*-OMe) compared with **2d** (*p*-OMe), as well as the strong activity of **2g** (*m*,*m*-diOMe). Compounds bearing EDGs acting mainly through inductive effects, such as **2j** (*o*,*p*-diMe) and **2k** (*m*,*m*-diMe), showed moderate activity (IC_50_ < 14 μM). Overall, methoxy-substituted compounds performed better than methyl-substituted analogues, suggesting that, in addition to electron-donating effects, the methoxy oxygen atom acting as a hydrogen-bond acceptor may enhance interactions with the biological target. In general, disubstituted compounds were more active than monosubstituted ones.

In contrast, a strong EWG reduced antitrypanosomal activity. The cyano derivatives **2p** (*m*-CN) and **2q** (*p*-CN) were inactive against *T. brucei*, showing that this type of substituent is unfavourable for activity.

The mono-fluorinated compound **2n** (*m*-F) showed only modest potency (IC_50_ = 15.15 µM) and was less active than the corresponding *m*-methoxy analogue.

Overall, these results indicate that EDGs are important against *T. brucei*. In contrast, the substituent effects against *L. infantum* promastigotes revealed distinct electronic requirements. In particular, the introduction of EW cyano groups significantly enhanced activity against *L. infantum*. Both **2p** and **2q** were active, with the *p*-cyano derivative **2q** showing especially strong potency (IC_50_ = 2.50 µM). This compound exhibited nearly four times greater activity against *L. infantum* promastigotes than the reference drug miltefosine, and its SI was approximately four times higher.

Methoxylated derivatives were generally less effective against *L. infantum*, with only **2e** (*o*,*p*-diOMe) demonstrating moderate activity. Thus, whereas EDGs are advantageous for antitrypanosomal activity, they are not good for antileishmanial potency.

The amino pyridine derivatives **2r** and **2s**, as well as the tertiary heteroalicyclic amine derivatives **2t** and **2u**, were inactive against both parasites. This lack of activity may be attributed to the absence of an N-H hydrogen bond donor (HBD) in the alicyclic amine derivatives. Moreover, when the amine is linked to the pyridine ring, its hydrogen bond donor capacity may be reduced due to electronic effects, which could further contribute to the loss of biological activity.

## 3. Materials and Methods

### 3.1. Chemistry

The reactions were monitored using thin-layer chromatography (TLC) using aluminium-backed silica gel 60 with fluorescent indicator UV_254_. The melting points were determined on a Stuart MP3 melting point apparatus (Stuart Scientific, Staffordshire, UK) and are uncorrected. Ether refers to diethyl ether and petroleum ether refers to petroleum ether 40–60 °C. ^1^H and ^13^C NMR were performed on a Bruker Avance III 400 (Bruker, Bremen, Germany), at 400 MHz for ^1^H and 100.6 MHz for ^13^C, using DEPT θ of 135 ^°^ to differentiate the type of carbons and ^1^H−^13^C (HSQC, HMBC) bidimensional correlations, to identify some signals. HRMS (ESI) results were obtained at The Q Exactive™ Plus (Thermofisher Scientific, Waltham, MA, USA) a quadrupole–orbitrap™ hybrid with an atmospheric pressure ionization (ESI).

#### General Procedure for the Synthesis of Di(hetero)arylamines **2a**–**u**

To a dried Schlenk tube charged with dry toluene or dry 1,4-dioxane (2–3 mL), Pd(OAc)_2_ (6–16 mol%) or Pd_2_(dba)_3_ (6 mol%), *rac*-BINAP or Xanthphos (8–18 mol%), compound **1** (0.100 g, 0.367 mmol), (hetero)arylamines or cyclic saturated heterocyclic amines (1.1–1.2 equiv.) and Cs_2_CO_3_ (1.4–2.0 equiv.) were added under Ar, stirred and heated at 100–110 °C for 2 h–24 h (controlled by TLC). After cooling, AcOEt (5 mL) and H_2_O (5 mL) were added and the phases were separated. The organic phase was washed with H_2_O (2 × 5 mL) and brine (5 mL) to give a residue, which was submitted to column chromatography using ether/petroleum ether mixtures, starting from 10% ether and increasing by 10% of ether each time the polarity of the mixture was changed until 50% ether/petroleum ether (controlling by TLC).

When the compound precipitated, it was filtered under vacuum and washed with ether.

*Methyl 5-(phenylamino)thieno[2,3-b]pyridine-2-carboxylate* (**2a**): Following the general procedure and using Pd(OAc)_2_ (8.00 mg, 0.0367 mmol), *rac*-BINAP (29.0 mg, 0.0490 mmol), compound **1**, aniline (30 μL, 0.304 mmol) and Cs_2_CO_3_ (0.240 g, 0.290 mmol). Column chromatography of the residue gave compound **2a** as a yellow solid (39.0 mg, 61%), m.p. 132.4–133.6 °C. ^1^H NMR (400 MHz, DMSO-*d*_6_) δ = 3.88 (3H, s, OCH_3_), 6.91 (1H, m, Ar-H), 7.15 (2H, m, 2 × Ar-H), 7.29 (2H, m, 2 × Ar-H), 8.06 (1H, d, *J* = 2.8 Hz, 4-H), 8.07 (1H, s, 3-H), 8.48 (1H, d, *J* = 2.8 Hz, 6-H), and 8.56 (1H, s, NH) ppm. ^13^C (100.6 MHz, DMSO-*d*_6_) δ = 52.8 (OCH_3_), 117.1 (4-CH), 117.3 (2 × CH), 121.0 (CH), 129.0 (3-CH), 129.5 (2 × CH), 132.5 (C), 132.8 (C), 138.3 (C), 142.4 (C), 142.9 (6-CH), 152.8 (C), and 162.4 (C=O) ppm. HRMS (ESI/[M+H]^+^): calculated *m*/*z* C_15_H_13_N_2_O_2_S: 285.0693; found: 285.0690 (−1.1 ppm).
*Methyl 5-[(2-methoxyphenyl)amino]thieno[2,3-b]pyridine-2-carboxylate* (**2b**): Following the general procedure and using Pd(OAc)_2_ (8.00 mg, 0.0367 mmol), *rac*-BINAP (29.0 mg, 0.0490 mmol), compound **1**, 2-methoxyaniline (46 μL, 0.404 mmol) and Cs_2_CO_3_ (0.240 g, 0.29 mmol). Column chromatography of the residue gave compound **2b** as a yellow solid (86.0 mg, 75%), m.p. 143.6–144.8 °C. ^1^H NMR (400 MHz, DMSO-*d*_6_) δ = 3.81 (3H, s, 2′-OCH_3_), 3.87 (3H, s, CO*OCH*_3_), 6.90 (1H, app td, *J* = 7.6 and 1.6 Hz, Ar-H), 7.00 (1H, app td, *J* = 7.6 and 1.6 Hz, Ar-H), 7.06 (1H, dd, *J* = 8.0 and 1.6 Hz, Ar-H), 7.25 (1H, dd, *J* = 8.0 and 1.6 Hz, Ar-H), 7.80 (1H, d, *J* = 2.8 Hz, 4-H), 7.92 (1H, s, NH), 8.04 (1H, s, 3-H), and 8.50 (1H, d, *J* = 2.8 Hz, 6-H) ppm. ^13^C (100.6 MHz, DMSO-*d*_6_) δ = 52.7 (2′-OCH_3_), 55.4 (CO*OCH*_3_), 111.9 (Ar-CH), 116.7 (4-CH), 118.6 (Ar-CH), 120.8 (Ar-CH), 122.6 (Ar-CH), 128.9 (3-CH), 130.7 (C), 132.1 (C), 132.6 (C), 139.0 (C), 142.7 (6-CH), 150.6 (2′-C), 152.3 (C), and 162.3 (C=O) ppm. HRMS (ESI/[M+H]^+^): calculated *m*/*z* C_16_H_15_N_2_O_3_S: 315.0798; found: 315.0791 (−2.2 ppm).
*Methyl 5-[(3-methoxyphenyl)amino]thieno[2,3-b]pyridine-2-carboxylate* (**2c**): Following the general procedure and using Pd(OAc)_2_ (8.00 mg, 0.0367 mmol), *rac*-BINAP (29.0 mg, 0.0490 mmol), compound **1**, 3-methoxyaniline (45 μL, 0.404 mmol) and Cs_2_CO_3_ (0.240 g, 0.29 mmol). Column chromatography of the residue gave compound **2c** as a yellow solid (58.0 mg, 50%), m.p. 164.2–165.4 °C. ^1^H NMR (400 MHz, DMSO-*d*_6_) δ = 3.73 (3H, s, 3′-OCH_3_), 3.88 (3H, s, CO*OCH*_3_), 6.48–6.51 (1H, m, Ar-H), 6.67 (1H, app t, *J* = 2.4 Hz, 2′-H), 6.71–6.74 (1H, m, Ar-H), 7.19 (1H, app t, *J* = 8.0 Hz, 5′-H), 8.09 (1H, d, *J* = 2.8 Hz, 4-H), 8.10 (1H, s, 3-H), 8.48 (1H, d, *J* = 2.8 Hz, 6-H), and 8.57 (1H, s, NH) ppm. ^13^C (100.6 MHz, DMSO-*d*_6_) δ = 52.8 (3′-OCH_3_), 54.9 (CO*OCH*_3_), 102.7 (2′-CH), 106.4 (Ar-CH), 109.5 (Ar-CH), 117.5 (4-CH), 129.0 (3-CH), 130.2 (5′-CH), 132.4 (C), 133.0 (C), 138.0 (C), 143.0 (6-CH), 153.0 (C), 160.3 (3′-C), and 162.3 (C=O) ppm. HRMS (ESI/[M+H]^+^): calculated *m*/*z* C_16_H_15_N_2_O_3_S: 315.0798; found: 315.0794 (−3.2 ppm).
*Methyl 5-[(4-methoxyphenyl)amino]thieno[2,3-b]pyridine-2-carboxylate* (**2d**): Following the general procedure and using Pd(OAc)_2_ (8.00 mg, 0.0367 mmol), *rac*-BINAP (29.0 mg, 0.0490 mmol), compound **1**, 4-methoxyaniline (50.0 mg, 0.404 mmol) and Cs_2_CO_3_ (0.240 g, 0.29 mmol). Column chromatography of the residue gave compound **2d** as a yellow solid (70.0 mg, 60%), m.p. 164.2–165.4 °C. ^1^H NMR (400 MHz, DMSO-*d*_6_) δ = 3.73 (3H, s, 4′-OCH_3_), 3.87 (3H, s, CO*OCH*_3_), 6.92 (2H, d, *J* = 8.8 Hz, 3′ and 5′-H), 7.13 (2H, d, *J* = 8.8 Hz, 2′ and 6′-H), 7.81 (1H, d, *J* = 2.8 Hz, 4-H), 8.02 (1H, s, 3-H), 8.28 (1H, s, NH), and 8.39 (1H, d, *J* = 2.8 Hz, 6-H) ppm. ^13^C (100.6 MHz, DMSO-*d*_6_) δ = 52.7 (CO*OCH*_3_), 55.3 (4′-OCH_3_), 114.3 (4-CH), 114.8 (3′ and 5′-CH), 121.1 (2′ and 6′-CH), 128.9 (3-CH), 132.2 (C), 132.8 (C), 134.9 (C), 140.1 (C), 141.7 (6-CH), 151.6 (C), 154.6 (4′-C), and 162.3 (C=O) ppm. HRMS (ESI/[M+H]^+^): calculated *m*/*z* C_16_H_15_N_2_O_3_S: 315.0798; found: 315.0791 (−2.2 ppm).
*Methyl 5-[(2,4-dimethoxyphenyl)amino]thieno[2,3-b]pyridine-2-carboxylate* (**2e**): Following the general procedure and using Pd(OAc)_2_ (8.00 mg, 0.0367 mmol), *rac*-BINAP (29.0 mg, 0.0490 mmol), compound **1**, 2,4-dimethoxyaniline (62.0 mg, 0.404 mmol) and Cs_2_CO_3_ (0.240 g, 0.29 mmol). Column chromatography of the residue gave compound **2e** as a yellow solid (82.0 mg, 65%), m.p. 132.4–132.6 °C. ^1^H NMR (400 MHz, DMSO-*d*_6_) δ = 3.76 (3H, s, OCH_3_), 3.77 (3H, s, OCH_3_), 3.86 (3H, s, CO*OCH*_3_), 6.53 (1H, dd, *J* = 8.0 and 2.8 Hz, 5′-H), 6.67 (1H, d, *J* = 2.8 Hz, 3′-H), 7.15 (1H, d, *J* = 8.8 Hz, 6′-H), 7.42 (1H, d, *J* = 2.8 Hz, 4-H), 7.72 (1H, s, NH), 7.98 (1H, s, 3-H), and 8.35 (1H, d, *J* = 2.8 Hz, 6-H) ppm. ^13^C (100.6 MHz, DMSO-*d*_6_) δ = 52.7 (CO*OCH*_3_), 55.3 (OCH_3_), 55.5 (OCH_3_), 99.8 (3′-CH), 104.7 (5′-CH), 113.9 (4-CH), 122.7 (C), 123.9 (6′-CH), 128.8 (3-CH), 131.9 (C), 132.7 (C), 141.2 (6-CH), 151.0 (C), 153.7 (C), 156.9 (C), and 162.4 (C=O) ppm. HRMS (ESI/[M+H]^+^): calculated *m*/*z* C_17_H_17_N_2_O_4_S:345.0904; found: 345.0895 (−2.6 ppm).
*Methyl 5-[(3,4-dimethoxyphenyl)amino]thieno[2,3-b]pyridine-2-carboxylate* (**2f**): Following the general procedure and using Pd(OAc)_2_ (8.00 mg, 0.0367 mmol), *rac*-BINAP (29.0 mg, 0.0490 mmol), compound **1**, 3,4-dimethoxyaniline (62.0 mg, 0.404 mmol) and Cs_2_CO_3_ (0.240 g, 0.290 mmol). Column chromatography of the residue gave compound **2f** as a yellow solid (63.0 mg, 50%), m.p. 136.0–137.0 °C. ^1^H NMR (400 MHz, DMSO-*d*_6_) δ = 3.72 (3H, s, OCH_3_), 3.73 (3H, s, OCH_3_), 3.87 (3H, s, CO*OCH*_3_), 6.71 (1H, dd, *J* = 8.8 and 2.4 Hz, 6′-H), 6.77 (1H, d, *J* = 2.4 Hz, 2′-H), 6.91 (1H, d, *J* = 8.8 Hz, 5′-H), 7.89 (1H, d, *J* = 2.8 Hz, 4-H), 8.04 (1H, s, 3-H), 8.31 (1H, s, NH), and 8.41 (1H, d, *J* = 2.8 Hz, 6-H) ppm. ^13^C (100.6 MHz, DMSO-*d*_6_) δ = 52.7 (OCH_3_), 55.4 (OCH_3_), 56.0 (CO*OCH*_3_), 104.7 (2′-CH), 110.8 (6′-CH), 113.1 (5′-CH), 115.0 (4-CH), 129.0 (3-CH), 132.2 (C), 132.8 (C), 135.5 (C), 139.8 (C), 141.9 (6-CH), 144.2 (C), 149.6 (C), and 151.8 (C), 162.4 (C=O) ppm. HRMS (ESI/[M+H]^+^): calculated *m*/*z* C_17_H_17_N_2_O_4_S: 345.0904; found: 345.0901 (−0.9 ppm).
*Methyl 5-[(3,5-dimethoxyphenyl)amino]thieno[2,3-b]pyridine-2-carboxylate* (**2g**): Following the general procedure and using Pd(OAc)_2_ (8.00 mg, 0.0367 mmol), *rac*-BINAP (29.0 mg, 0.0440 mmol), compound **1**, 3,5-dimethoxyaniline (62.0 mg, 0.404 mmol) and Cs_2_CO_3_ (0.240 g, 0.290 mmol). Column chromatography of the residue gave compound **2g** as a yellow solid (63.0 mg, 50%), m.p. 104.4–105.6 °C. ^1^H NMR (400 MHz, DMSO-*d*_6_) δ = 3.70 (6H, s, OCH_3_), 3.88 (3H, s, CO*OCH*_3_), 6.08 (1H, t, *J* = 2.0 Hz, 4’-H), 6.27 (2H, *J* = 2.0 Hz, 2’ and 6’-H), 8.11-8.12 (2H, m, 3 and 4-H), 8.47 (1H, d, *J* = 2.8 Hz, 6-H), and 8.57 (1H, s, NH) ppm. ^13^C (100.6 MHz, DMSO-*d*_6_) δ = 52.8 (CO*OCH*_3_), 55.1 (2 × OCH_3_), 93.1 (4′-CH), 95.3 (2′ and 6′-CH), 118.2 (4-CH), 129.2 (3-CH), 132.5 (C), 132.8 (C), 137.9 (C), 143.2 (6-CH), 144.4 (C), 153.2 (C), 161.3 (C), and 162.3 (C) ppm. HRMS (ESI/[M+H]^+^): calculated *m*/*z* C_17_H_17_N_2_O_4_S:345.0904; found: 345.0898 (−1.7 ppm).
*Methyl 5-(3-tolylamino)thieno[2,3-b]pyridine-2-carboxylate* (**2h**): Following the general procedure and using Pd(OAc)_2_ (82.0 mg, 0.0367 mmol), *rac*-BINAP (29.0 mg, 0.0490 mmol), compound **1**, *m*-toluidine (47.0 mg, 0.440 mmol) and Cs_2_CO_3_ (0.167 g, 0.514 mmol). Column chromatography of the residue gave compound **2h** as a yellow solid (66.0 mg, 60%), m.p. 174.0–176.0 °C. ^1^H NMR (400 MHz, DMSO-*d*_6_) δ = 2.26 (3H, s, CH_3_), 3.88 (3H, s, OCH_3_), 6.74 (1H, broad d, *J* = 8 Hz, 6′-H), 6.94 (2H, m, 2′-H and 4′-H), 7.17 (1H, app. t, *J* = 8 Hz, 5′-H), 8.04 (1H, d, *J* = 2.4 Hz, 4-H), 8.08 (1H, s, 3-H), 8.47 (1H, d, *J* = 2.4 Hz, 6-H), and 8.48 (1H, broad s, NH) ppm. ^13^C (100.6 MHz, DMSO-*d*_6_) δ = 21.2 (CH_3_), 52.8 (OCH_3_), 114.4 (4′-CH), 117.1 (4-CH), 117.8 (2′-CH), 121.7 (6′-CH), 129.0 (3-CH), 129.2 (5′-CH), 132.4 (C), 132.7 (C), 138.3 (C), 138.7 (C), 142.4 (C), 142.8 (6-CH), 152.7 (C), and 162.3 (C=O) ppm. HRMS (ESI/[M+H]^+^): calculated *m*/*z* ([C_16_H_14_N_2_O_2_S]^+^): 299.0849, found 299.0847 (−0.7 ppm).
*Methyl 5-(4-tolylamino)thieno[2,3-b]pyridine-2-carboxylate* (**2i**): Following the general procedure and using Pd(OAc)_2_ (82.0 mg, 0.0367 mmol), *rac*-BINAP (29.0 mg, 0.0440 mmol), compound **1**, *p*-toluidine (47.0 mg, 0.440 mmol) and Cs_2_CO_3_ (0.240 g, 0.734 mmol). Column chromatography of the residue gave compound **2i** as a yellow solid (70.0 mg, 65%), m.p. 180.0–182.2 °C. ^1^H NMR (400 MHz, DMSO-*d*_6_) δ = 2.25 (3H, s, CH_3_), 3.88 (3H, s, OCH_3_), 7.06 (2H, d, *J* = 8.8 Hz, 2′-H and 6′-H), 7.11 (2H, d, *J* = 8.8 Hz, 3′H and 5′-H), 7.96 (1H, d, *J* = 2.8 Hz, 4-H), 8.05 (1H, s, 3-H), 8.42 (1H, broad s, NH), and 8.44 (1H, d, *J* = 2.8 Hz, 6-H) ppm. ^13^C (100.6 MHz, DMSO-*d*_6_) δ = 20.3 (CH_3_), 52.7 (OCH_3_), 115.8 (4-CH), 118.1 (2′ and 6′-CH), 128.9 (3-CH), 129.8 (3′ and 5′-CH), 130.1 (C), 132.3 (C), 132.7 (C), 138.9 (C), 139.6 (C), 142.3 (6-CH), 152.2 (C), and 162.3 (C=O) ppm. HRMS (ESI/[M+H]^+^): calculated *m*/*z* ([C_16_H_14_N_2_O_2_S]^+^): 299.0849, found 299.0845 (−1.3 ppm).
*Methyl 5-[(2,4-dimethylphenyl)amino]thieno[2,3-b]pyridine-2-carboxylate* (**2j**): Following the general procedure and using Pd(OAc)_2_ (82.0 mg, 0.0367 mmol), *rac*-BINAP (29.0 mg, 0.0440 mmol), compound **1**, 2,4-dimethylaniline (53.0 mg, 0.440 mmol) and Cs_2_CO_3_ (0.240 g, 0.734 mmol). Column chromatography of the residue gave compound **2j** as a yellow solid (73.0 mg, 65%), m.p. 136.0–138.0 °C. ^1^H NMR (400 MHz, DMSO-*d*_6_) δ = 2.15 (3H, s, CH_3_), 2.25 (3H, s, CH_3_), 3.86 (3H, s, OCH_3_), 6.99 (1H, dd, *J =* 8.0 and 2.0 Hz, 5′-H), 7.08–7.11 (2H, m, 3′ and 6′-H), 7.46 (1H, d, *J* = 2.8 Hz, 4-H), 7.82 (1H, broad s, NH), 7.99 (1H, s, 3-H), and 8.39 (1H, d, *J* = 2.8 Hz, 6-H) ppm. ^13^C (100.6 MHz, DMSO-*d*_6_) δ = 17.8 (CH_3_), 20.4 (CH_3_), 52.7 (OCH_3_), 114.7 (4-CH), 122.1 (6′-CH), 127.4 (5′-CH), 128.8 (3-CH), 131.3 (C), 131.7 (3′-CH), 132.1 (C), 132.7 (C), 132.8 (C), 137.2 (C), 140.8 (C), 141.5 (6-CH), 151.4 (C), and 162.3 (C=O) ppm. HRMS (ESI/[M+H]^+^): calculated *m*/*z* ([C_17_H_16_N_2_O_2_S]^+^): 313.1006, found 313.1000 (−1.9 ppm).
*Methyl 5-[(3,5-dimethylphenyl)amino]thieno[2,3-b]pyridine-2-carboxylate* (**2k**): Following the general procedure and using Pd(OAc)_2_ (82.0 mg, 0.0367 mmol), *rac*-BINAP (29.0 mg, 0.0440 mmol), compound **1**, 3,5-dimethylaniline (47.0 mg, 0.440 mmol) and Cs_2_CO_3_ (0.167 g, 0.514 mmol). Column chromatography of the residue gave compound **2k** as a yellow solid (69.0 mg, 60%), m.p. 155.0–157.0 °C. ^1^H NMR (400 MHz, DMSO-*d*_6_) δ = 2.21 (6H, s, 2 × CH_3_), 3.87 (3H, s, OCH_3_), 6.55 (1H, s, 4′-H), 6.75 (1H, s, 2′-H and 6′-H), 8.02 (1H, d, *J* = 2.8 Hz, 4-H), 8.08 (1H, s, 3-H), 8.39 (1H, s, NH), and 8.45 (1H, d, *J* = 2.8 Hz, 6-H) ppm. ^13^C (100.6 MHz, DMSO-*d*_6_) δ = 21.1 (2 × CH_3_), 52.8 (OCH_3_), 115.1 (2′-CH and 6′-CH), 117.2 (4-CH), 122.7 (4′-CH), 129.0 (3-CH), 132.3 (C), 132.7 (C), 138.4 (2 × C), 142.3 (C), 142.8 (6-CH), 152.6 (C), and 162.3 (C=O) ppm. HRMS (ESI/[M+H]^+^): calculated *m*/*z* ([C_17_H_16_N_2_O_2_S]^+^): 313.1006, found 313.1001 (−1.9 ppm).
*Methyl 5-[(2-chloro-5-methoxyphenyl)amino]thieno[2,3-b]pyridine-2-carboxylate* (**2l**): Following the general procedure and using Pd(OAc)_2_ (8.00 mg, 0.0367 mmol), *rac*-BINAP (29.0 mg, 0.049 mmol), compound **1**, 2-chloro-5-methoxyaniline (75.0 mg, 0.404 mmol) and Cs_2_CO_3_ (0.240 g, 0.290 mmol). Column chromatography of the residue gave compound **2l** as a yellow solid (57.0 mg, 45%), m.p. 196.5–197.7 °C. ^1^H NMR (400 MHz, DMSO-*d*_6_) δ = 3.69 (3H, s, OCH_3_), 3.88 (3H, s, OCH_3_), 6.60 (1H, dd, *J* = 8.8 and 2.8 Hz, 4′-H), 6.81 (1H, d, *J* = 2.8 Hz, 6′-H), 7.37 (1H, d, *J* = 8.8 Hz, 3′-H), 7.96 (1H, d, *J* = 2.8 Hz, 4-H), 8.10 (1H, s, 3-H), 8.14 (1H, s, NH), and 8.56 (1H, d, *J* = 2.8 Hz, 6-H) ppm. ^13^C (100.6 MHz, DMSO-*d*_6_) δ = 52.8 (OCH_3_), 55.4 (OCH_3_), 104.8 (6′-CH), 108.7 (4′-CH), 115.4 (C), 119.8 (4-CH), 129.0 (3-CH), 130.7 (3′-CH), 132.6 (C), 137.7 (C), 140.2 (C), 143.7 (6-CH), 154.0 (C), 158.9 (C), and 162.3 (C=O) ppm. HRMS (ESI) [M+H]^+^, *m*/*z* Calculated for C_16_H_14_ClN_2_O_3_S: 349.0409; found: 349.0407 (−0.6 ppm).
*Methyl 5-[(2-fluorophenyl)amino]thieno[2,3-b]pyridine-2-carboxylate* (**2m**): Following the general procedure and using Pd(OAc)_2_ (8.00 mg, 0.0367 mmol), Xantphos (25.0 mg, 0.044 mmol), compound **1**, *o*-fluoroaniline (45.0 mg, 0.404 mmol) and Cs_2_CO_3_ (0.240 g, 0.290 mmol). The mixture was heated for 6 h. Upon cooling, H_2_O (5 mL) was added to the reaction mixture and a yellow solid precipitated. This was collected by filtration, washed with H_2_O, and dried at 50 °C. The solid was further washed with some ether to afford **2m** (61.0 mg, 60%) as a yellow solid, m.p. 206.0–208.0 °C. ^1^H NMR (400 MHz, DMSO-*d*_6_) δ = 3.88 (3H, s, OCH_3_), 7.02–7.05 (1H, m, 4′-H), 7.12–7.16 (1H, m, Ar-H), 7.24–7.29 (1H, m, 3′-H), 7.33–7.38 (1H, m, Ar-H), 7.85–7.86 (1H, m, 4-H), 8.07 (1H, s, 3-H), 8.40 (1H, broad s, NH), and 8.50 (1H, d, *J* = 2.8 Hz, 6-H) ppm. ^13^C (100.6 MHz, DMSO-*d*_6_) δ = 52.8 (OCH_3_), 116.2 (d, *J* = 19.0 Hz, 3′-CH), 117.5 (4-CH), 120.4 (d, *J* = 2.7 Hz, CH), 122.8 (d, *J* = 7.4 Hz, 4′-CH), 125.0 (d, *J* = 3.5 Hz, CH), 128.9 (3-CH), 129.9 (d, *J* = 11.1 Hz, C), 132.6 (d, *J* = 11.1 Hz, C), 138.3 (C), 142.5 (CH), 153.1 (C), 154.0 (d, *J* = 243.4 Hz, C-F), and 162.3 (C=O) ppm. HRMS (ESI/[M+H]^+^): calculated for *m*/*z* [C_15_H_11_F N_2_O_2_S]^+^: 303.0599, found 303.0594 (−1.6 ppm).
*Methyl 5-[(3-fluorophenyl)amino]thieno[2,3-b]pyridine-2-carboxylate* (**2n**): Following the general procedure and using Pd(OAc)_2_ (8.00 mg, 0.0367 mmol), Xantphos (25.0 mg, 0.044 mmol), compound **1**, *m*-fluoroaniline (45.0 mg, 0.404 mmol) and Cs_2_CO_3_ (0.240 g, 0.290 mmol). The mixture was heated for 5 h. Upon cooling, H_2_O (5 mL) was added to the reaction mixture and a yellow solid precipitated. This was collected by filtration, washed with H_2_O, and dried at 50 °C. The solid was further washed with some ether to afford **2n** (61.0 mg, 55%) as a yellow solid, m.p. 198.0–200.0 °C. ^1^H NMR (400 MHz, DMSO-*d*_6_) δ = 3.88 (3H, s, OCH_3_), 6.65–6.69 (1H, m, 4′-H), 6.88–6.94 (2H, m, 2′ and 6′-H), 7.26–7.32 (1H, m, 5′-H), 8.10 (1H, s, 3-H), 8.16 (1H, d, *J* = 2.4 Hz, 4-H), 8.50 (1H, d, *J* = 2.8 Hz, 6-H), and 8.79 (1H, br s, N-H) ppm. ^13^C (100.6 MHz, DMSO-*d*_6_) δ = 52.9 (OCH_3_), 102.9 (d, *J* = 25.1 Hz, 2′-CH), 106.8 (d, *J* = 22.1 Hz, 4′-CH), 112.5 (d, *J* = 2.0 Hz, 6′-CH), 119.0 (4-CH), 129.1 (3-CH), 131.1 (d, *J* = 11.1 Hz, 5′-CH), 132.7 (d, *J* = 5.0 Hz, 1′-C), 137.2 (C), 143.4 (6-CH), 144.8 (C), 145.0 (C), 153.9 (C), 162.3 (C=O), and 163.2 (d, *J* = 242.4 Hz, C-F) ppm. HRMS (ESI/[M+H]^+^): calculated for *m*/*z* [C_15_H_11_FN_2_O_2_S]^+^: 303.0599, found 303.0593 (−2.0 ppm).
*Methyl 5-[(4-fluorophenyl)amino]thieno[2,3-b]pyridine-2-carboxylate* (**2o**): Following the general procedure and using Pd(OAc)_2_ (8.00 mg, 0.0367 mmol), Xantphos (25.0 mg, 0.044 mmol), compound **1**, *p*-fluoroaniline (45.0 mg, 0.404 mmol) and Cs_2_CO_3_ (0.240 g, 0.290 mmol). Column chromatography of the residue gave compound **2o** as a yellow solid (53.0 mg, 50%), m.p. 201.0–203.0 °C. ^1^H NMR (400 MHz, DMSO-*d*_6_) δ = 3.87 (3H, s, OCH_3_), 7.11–7.19 (4H, m, Ar-H), 7.97 (1H, d, *J* = 2.8 Hz, 4-H), 8.04 (1H, s, 3-H), 8.44 (1H, d, *J* = 2.8 Hz, 6-H), and 8.51 (1H, br s, NH) ppm. ^13^C (100.6 MHz, DMSO-*d*_6_) δ = 52.9 (OCH_3_), 116.1 (d, *J* = 22.1 Hz, 3′ and 5′-CH), 116.2 (4-CH), 119.7 (d, *J* = 8.0 Hz, 2′ and 6′-CH), 128.9 (3-CH), 132.5 (C), 132.8 (C), 138.7 (d, J = 2.0 Hz, 1′-C), 138.9 (C), 142.4 (6-CH), 152.6 (C), 162.4 (C=O), and 157.2 (d, *J* = 227.3 Hz, C-F) ppm. HRMS (ESI/[M+H]^+^): calculated for *m*/*z* [C_15_H_11_FN_2_O_2_S]^+^: 303.0599, found 303.0594 (−1.6 ppm).
*Methyl 5-[(3-cyanophenyl)amino]thieno[2,3-b]pyridine-2-carboxylate* (**2p**): Following the general procedure and using Pd(OAc)_2_ (82.0 mg, 0.0367 mmol), Xantphos (25.0 mg, 0.044 mmol), compound **1**, 3-aminobenzonitrile (48.0 mg, 0.404 mmol) and Cs_2_CO_3_ (0.240 g, 0.734 mmol). Column chromatography of the residue gave compound **2p** as a yellow solid (55.0 mg, 50%), m.p. 212.0–214.0 °C. ^1^H NMR (400 MHz, DMSO-*d*_6_) δ = 3.88 (3H, s, OCH_3_), 7.27 (1H, dt, *J* = 7.2 and 1.2 Hz, 3′-H), 7.38–7.47 (3H, m, 3 × Ar-H), 8.10 (1H, s, 3-H), 8.18 (1H, d, *J* = 2.8 Hz, 4-H), 8.51 (1H, d, *J* = 2.4 Hz, 6-H), 8.91 (1H, s, NH) ppm. ^13^C (100.6 MHz, DMSO-*d*_6_) δ = 52.9 (OCH_3_), 112.3 (C), 118.3 (Ar-CH), 119.0 (C), 119.7 (4-CH), 121.0 (Ar-CH), 123.6 (3′-CH), 129.0 (3-CH), 130.8 (Ar-CH), 132.7 (C), 132.8 (C), 136.6 (C), 143.6 (6-CH), 144.0 (C), 154.3 (C), and 162.3 (C=O) ppm. HRMS (ESI/[M+H]^+^): calculated *m*/*z* ([C_16_H_11_N_3_O_2_S]^+^): 310.0645, found 310.0642 (−0.9 ppm).
*Methyl 5-[(4-cyanophenyl)amino]thieno[2,3-b]pyridine-2-carboxylate* (**2q**): Following the general procedure and using Pd(OAc)_2_ (82.0 mg, 0.0367 mmol), Xantphos (25.0 mg, 0.044 mmol), compound **1**, 4-aminobenzonitrile (48.0 mg, 0.404 mmol) and Cs_2_CO_3_ (0.240 g, 0.734 mmol). The mixture was heated for 8 h. After cooling, H_2_O (5 mL) was added to the reaction mixture and a yellow solid precipitated. This was collected by filtration, washed with H_2_O, and dried at 50 °C. The solid was further washed with some ether to afford **2q** (87.0 mg, 76%) as a yellow solid, m.p. 220.0–222.0 °C. ^1^H NMR (400 MHz, DMSO-*d*_6_) δ = 3.90 (3H,s, OCH_3_), 7.16 (2H,d, *J* = 8.8 Hz, 2′ and 6′-H), 7.64 (2H, d, *J* = 8.8 Hz, 3′ and 5′-H), 8.14 (1H, s, 3-H), 8.26 (1H, d, *J* = 2.8 Hz, 4-H), 8.57 (1H, d, *J* = 2.8 Hz, 6-H), 9.22 (1H, s, NH) ppm. ^13^C (100.6 MHz, DMSO-*d*_6_) δ = 52.9 (OCH_3_), 100.4 (C), 115.0 (2′ and 6′-CH), 119.7 (C), 122.1 (4-CH), 128.9 (3-CH), 132.6 (C), 133.0 (C), 133.8 (3′ and 5′-CH), 135.4 (C), 144.4 (6-CH), 147.7 (C), 155.3 (C), 162.2 (C=O) ppm. HRMS (ESI/[M+H]^+^): calculated *m*/*z* ([C_16_H_11_N_3_O_2_S]^+^): 310.0645, found 310.0642 (−0.9 ppm).
*Methyl 5-(pyridin-2-ylamino)thieno[2,3-b]pyridine-2-carboxylate* (**2r**): Following the general procedure and using Pd(OAc)_2_ (13.0 mg, 0.0587 mmol), Xanthphos (38.0 mg, 0.066 mmol), compound **1**, pyridin-2-amine (41.0 mg, 0.440 mmol) and Cs_2_CO_3_ (0.215 g, 0.661 mmol). Column chromatography of the residue gave compound **2r** as a yellow solid (50.0 mg, 50%), m.p. 228.0–230.0 °C. ^1^H NMR (400 MHz, DMSO-*d*_6_) δ = 3.89 (3H, s, OCH_3_), 6.84 (1H, m, 5′-H), 6.89 (1H, d, *J =* 8.4 Hz, 3′-H), 7.63 (1H, m, 4′-H), 8.17 (1H, s, 3-H), 8.21 (1H, dd, *J* = 5.2 and 1.2 Hz, 6′-H), 8.82 (1H, d, *J =* 2.4, HetAr-H), 8.94 (1H, d, *J* = 2.4 Hz, HetAr-H), and 9.49 (1H, s, NH) ppm. ^13^C (100.6 MHz, DMSO-*d*_6_) δ = 52.8 (OCH_3_), 107.8 (C), 111.2 (3′-CH), 115.2 (5′-CH), 119.8 (CH), 129.2 (3-CH), 132.4 (C), 136.3 (C), 137.7 (4′-CH), 142.8 (CH), 147.2 (6′-CH), 153.5 (C), 155.4 (C), and 162.4 (C=O) ppm. HRMS (ESI/[M+H]^+^): calculated *m*/*z* ([C_14_H_11_N_3_O_2_S]^+^): 286.0645, found 286.0640 (−1.7 ppm).
*Methyl 5-(pyridin-3-ylamino)thieno[2,3-b]pyridine-2-carboxylate* (**2s**): Following the general procedure and using Pd(OAc)_2_ (13.0 mg, 0.0587 mmol), Xanthphos (38.0 mg, 0.066 mmol), compound **1**, pyridin-3-amine (41.0 mg, 0.440 mmol) and Cs_2_CO_3_ (0.215 g, 0.661 mmol). Column chromatography of the residue gave compound **2s** as a yellow solid (75.0 mg, 70%), m.p. 193.0–195.0 °C. ^1^H NMR (400 MHz, DMSO-*d*_6_) δ = 3.89 (3H, s, OCH_3_), 7.30 (1H, dd, *J* = 8.2 and 4.8 Hz, 5′-H), 7.55–7.58 (1H, m, 4′-H), 8.09 (1H, s, 3-H), 8.11–8.14 (2H, m, HetAr-H), 8.43 (1H, d, *J* = 2.8 Hz, HetAr-H), 8.51 (1H, d, *J* = 2.8 Hz, HetAr-H), and 8.76 (1H, broad s, NH) ppm. ^13^C (100.6 MHz, DMSO-*d*_6_) δ = 52.8 (OCH_3_), 117.8 (CH), 123.0 (4′-CH), 124.0 (5′-CH), 128.9 (3-CH), 132.6 (C), 136.7 (C), 137.3 (C), 139.2 (C), 139.5 (C), 141.6 (CH), 142.9 (CH), 153.5 (C), and 162.2 (C=O) ppm. HRMS (ESI/[M+H]^+^): calculated *m*/*z* ([C_14_H_11_N_3_O_2_S]^+^):286.0645, found 286.0642 (−1.1 ppm).
*Methyl 5-morpholinothieno[2,3-b]pyridine-2-carboxylate* (**2t**): Following the general procedure and using Pd(OAc)_2_ (82.0 mg, 0.0367 mmol), *rac*-BINAP (29.0 mg, 0.0440 mmol), compound **1**, morpholine (19.0 mg, 0.223 mmol) and Cs_2_CO_3_ (0.167 g, 0.514 mmol). Column chromatography of the residue gave compound **2t** as a yellow solid (26.0 mg, 50%), m.p. 184.0–188.0 °C. ^1^H NMR (400 MHz, DMSO-*d*_6_) δ = 3.19–3.22 (4H, m, 2′ and 6′-CH_2_), 3.77–3.79 (4H, m, 3′ and 5′-CH_2_), 3.90 (3H, s, OCH_3_), 7.84 (1H, d, *J* = 2.8 Hz, 4-H), 8.05 (1H, s, 3-H), and 8.63 (1H, d, *J* = 2.8 Hz, 6-H) ppm. ^13^C (100.6 MHz, DMSO-*d*_6_) δ = 48.6 (2′ and 6′-CH_2_), 52.8 (OCH_3_), 65.9 (3′ and 5′-CH_2_), 117.3 (4-CH), 128.8 (3-CH), 132.5 (C), 132.6 (C), 141.9 (6-CH), 145.5 (C), 152.8 (C), and 162.3 (C=O) ppm. HRMS (ESI/[M+H]^+^): calculated for *m*/*z* ([C_13_H_14_N_2_O_3_S]^+^):279.0798, found 279.0795 (−1.1 ppm).
*Methyl 5-(piperidin-1-yl)thieno[2,3-b]pyridine-2-carboxylate* (**2u**): Following the general procedure and using Pd_2_(dba)_3_ (20.0 mg,0.022 mmol), Xantphos (17.0 mg, 0.030 mmol), compound **1**, piperidine (37.0 mg, 0.440 mmol) and Cs_2_CO_3_ (0.167 g, 0.514 mmol). Column chromatography of the residue gave compound **2u** as a yellow solid (51.0 mg, 50%), m.p. 122.0–124.0 °C. ^1^H NMR (400 MHz, DMSO-*d*_6_) δ = 1.50–1.56 (2H, m, 4′-CH_2_), 1.61–1.66 (4H, m, 2 × CH_2_), 3.19 (4H, m, 2 × CH_2_), 3.88 (3H, s, OCH_3_), 7.80 (1H, d, *J* = 2.8 Hz, 4-H), 8.02 (1H, s, 3-H), and 8.58 (1H, d, *J* = 2.8 Hz, 6-H) ppm. ^13^C (100.6 MHz, DMSO-*d*_6_) δ = 23.7 (4′-CH_2_), 25.1 (3′ and 5′-CH_2_), 49.9 (2′ and 6′-CH_2_), 52.8 (OCH_3_), 117.6 (4-CH), 128.9 (3-CH), 132.3 (C), 132.8 (C), 142.8 (6-CH), 146.1 (C), 152.2 (C), and 162.4 (C=O) ppm. HRMS (ESI/[M+H]^+^): calculated for *m*/*z* ([C_14_H_16_N_2_O_2_S]^+^):277.1006, found 277.1003 (−1.1 ppm).

### 3.2. In Vitro Antiparasitic Assays

#### 3.2.1. Parasite Cultures

*Trypanosoma brucei* Lister 427 bloodstream forms were cultured in complete HMI-9 medium supplemented with 10% heat-inactivated fetal bovine serum (FBS) and 100 IU/mL penicillin/streptomycin at 37 °C in a humidified 5% CO_2_ incubator. Parasites were maintained in T25 ventilated flasks and subpassaged every 2 days at a density of 1 × 10^4^ cells/mL. *Leishmania infantum* MHOM/MA/67/ITMAP-263 promastigotes were cultured at 27 °C in complete RPMI-1640 medium supplemented with 10% heat-inactivated FBS, 2 mM L-glutamine, 100 IU/mL penicillin/streptomycin, and 20 mM HEPES. Parasites were maintained in T25 non-ventilated flasks and subpassaged every 5 days at a density of 1 × 10^6^ cells/mL [13].

Luciferase-expressing *L. infantum* (MHOM/MA/67/ITMAP-263) axenic amastigotes carrying episomal luciferase were maintained in MAA/20 medium at 37 °C in a humidified 5% CO_2_ incubator. Subculturing was performed every 7 days at a density of 1 × 10^6^ cells/mL in 5 mL using T25 ventilated flasks [14].

#### 3.2.2. Cell Cultures

The human leukemia cell line THP-1 (ATCC^®^ TIB-202™, American Type Culture Collection, Manassas, VA, USA) was cultured in RPMI-1640 medium supplemented with 10% heat-inactivated FBS, 2 mM L-glutamine, 100 IU/mL penicillin/streptomycin, and 20 mM HEPES. Cells were maintained in a humidified incubator at 37 °C with 5% CO_2_ and subcultured every 3 days in 20 mL of medium at a density of 1 × 10^5^ cells/mL in T 75 flasks. All cell culture reagents were purchased from Lonza Bioscience (Morrisville, NC, USA).

#### 3.2.3. *In Vitro* Anti-*T. brucei* Activity Assays

The activity of the synthesized compounds against *Trypanosoma brucei* bloodstream forms was evaluated using a modified resazurin-based assay as previously described [15]. Parasites were seeded in 96-well plates at a density of 1 × 10^4^ cells/mL in 100 μL of supplemented complete medium containing serial dilutions of the tested compounds. The final assay volume was adjusted to 200 μL per well. Pentamidine was included in all assays as a reference antitrypanosomal drug for quality control. Each condition was tested in duplicate. After 72 h of incubation under standard *T. brucei* culture conditions, 20 μL of 0.5 mM resazurin solution was added to each well, and plates were incubated for an additional 4 h under the same conditions. Fluorescence was measured at 544 nm excitation and 590 nm emission using a Synergy 2 Multi-Mode Reader (BioTek, Winooski, VT, USA).

Results were expressed as the percentage of parasite growth inhibition relative to untreated controls and represent the mean of at least three independent experiments. The IC_50_ values were determined by non-linear regression analysis using GraphPad Prism version 8.1.1 for Windows (GraphPad Software, San Diego, CA, USA).

#### 3.2.4. Activity Against *L. infantum* MHOM/MA/67/ITMAP-263 Promastigotes

The efficacy of the compounds against *Leishmania infantum* MHOM/MA/67/ITMAP-263 promastigotes was evaluated using a resazurin-based assay. Parasites were seeded in 96-well plates at a density of 1 × 10^6^ cells/mL in 100 μL of supplemented complete medium containing serial dilutions of the tested compounds. The final assay volume was adjusted to 200 μL per well. Miltefosine was included in all assays as a reference antileishmanial drug for quality control. Each condition was tested in duplicate. After 72 h of incubation under standard *L. infantum* culture conditions, 20 μL of 0.5 mM resazurin solution was added to each well, followed by an additional 4 h incubation under the same conditions. Fluorescence was measured as described in Section 3.2.3. Results were expressed as the percentage of parasite growth inhibition relative to untreated controls, and IC_50_ values were determined as described for *T. brucei*.

#### 3.2.5. *In Vitro* Evaluation Against *L. infantum* Amastigotes on Infected Cells

The activity against *L. infantum* intracellular amastigotes was evaluated as described [16], with some modifications. Briefly, THP-1 cells were resuspended in RPMI complete medium at a density of 8 × 10^5^ cells/mL and 100 μL/well were seeded in a 96-well plate and were differentiated into macrophages by an addition of 40 ng/mL of phorbol-myristate 13-acetate (PMA, Sigma, Saint Louis, MI, USA) for 24 h followed by replacement with fresh medium for more 24 h. After that, cells were infected for 4 h with *L. infantum* axenic amastigotes expressing episomal luciferase in a macrophage: amastigotes ratio of 1:10 at 37 °C, 5% CO_2_. Non-internalized parasites were washed, and compounds were added at different concentrations in a final volume of 100 μL. As quality control, a dose–response curve to miltefosine was included in all assays. Each condition was carried out in quadruplicate. After 72 h of incubation, the media was substituted by 100 μL of PBS and 25 μL of Glo-lysis buffer from the Steady-Glo Luciferase Assay System (Promega, Madison, WI, USA) was added. The plates have been placed in an agitator at 100 rpm for 10 min at room temperature. Finally, 30 μL of the Steady-Glo reagent (Promega, Madison, WI, USA) was added to the plate and incubated for 15 min in the dark under the same conditions. A total of 140 μL of the well content was transferred to white-bottom 96-well plates. The luminescence intensity was read using a Synergy 2 Multi-Mode Reader (Biotek, Winooski, VT, USA). The antileishmanial effect was evaluated by the determination of the IC_50_ value and calculated by the non-linear regression analysis using GraphPad Prism version 8.1.1 for Windows (GraphPad Software, San Diego, CA, USA). The results represent the average of at least three independent experiments.

#### 3.2.6. Cytotoxicity in THP-1-Derived Macrophages

The cytotoxicity effect of compounds on THP-1-derived macrophages was assessed by the colorimetric MTT assay (3-(4,5-dimethylthiazol-2-yl)-2,5-diphenyl tetrazolium bromide). Briefly, THP-1 cells were suspended in RPMI complete medium at a density of 8 × 10^5^ cells/mL and 100 μL/well were seeded in a 96-well plate and were differentiated into macrophages by an addition of 40 ng/mL of phorbol-myristate 13-acetate (PMA, Sigma, Saint Louis, MI, USA) for 48 h followed by replacement with fresh medium for more 24 h. Subsequently, cells were incubated with 100 μL of compounds ranging from 100 to 12.5 μM after dilution in the RPMI complete medium. Each condition was carried out in quadruplicate. After 72 h of incubation at 37 °C, 5% CO_2_, the medium was removed and 200 μL of 0.5 mg/mL MTT solution diluted in RPMI was added. Plates were incubated for an additional 4 h. Then 160 μL of media was removed and the same volume of 2-propanol was added. Absorbance was read at 570 nm using a Synergy 2 Multi-Mode Reader (Biotek, Winooski, VT, USA). Cytotoxicity was evaluated by the determination of the CC_50_ value (drug concentration that reduced the percentage of viable cells in 50%) and calculated by non-linear regression analysis using GraphPad Prism version 8.1.1 for Windows (GraphPad Software, San Diego, CA, USA). The results represent the average of at least three independent experiments. For each compound, the selectivity index (SI) was calculated as the ratio between cytotoxicity in THP-1 (CC_50_, 72 h) and activity against parasites (IC_50_, 72 h).

## 4. Conclusions

A series of novel di(hetero)aryl and alicyclic amines were successfully synthesized via Pd-catalyzed Buchwald-Hartwig amination of methyl 5-bromothieno[2,3-*b*]pyridine-2-carboxylate with various (hetero)aromatic and alicyclic amines in good to high yields, demonstrating the broad applicability of this synthetic methodology. Biological evaluation against *T. brucei* and *L. infantum* revealed activity trends related to the amino substitution pattern at C(5), highlighting the important influence of the substituent electronic effects on antiparasitic activity. Among the methoxylated derivatives, *meta*-substitution favored activity against *T. brucei*, whereas the *o*,*p*-dimethoxylated analogue exhibited selective activity against *L. infantum*, indicating distinct structural requirements for each parasite.

For methyl-substituted derivatives, *meta*-substitution enhanced activity against *L. infantum*, while *para*- and dimethylated analogues displayed activity against both *para*-sites. Electron-withdrawing groups conferred more selective activity profiles: the *meta*-fluoro derivative showed activity against *T. brucei*, while cyano-substituted compounds, particularly the *para*-cyano analogue, demonstrated potent and selective activity against *L. infantum*, surpassing the reference drug miltefosine. In contrast, pyridine and tertiary alicyclic amine derivatives were inactive against both parasites.

Overall, these results elucidate the amino substituent effects at the C(5) position of the thieno[2,3-*b*]pyridine scaffold and provide a basis for the further design and optimization of selective antitrypanosomal and antileishmanial agents. The favorable safety profile, evidenced by negligible cytotoxicity toward PMA-differentiated THP-1 macrophages and the resulting high selectivity indices, further underscores the therapeutic potential of this chemical series for antiparasitic drug development.

## Figures and Tables

**Table 1:** Synthesis of methyl 5-aminothieno[2,3-*b*]pyridine-2-carboxylates derivatives (**2a**−**2u**).

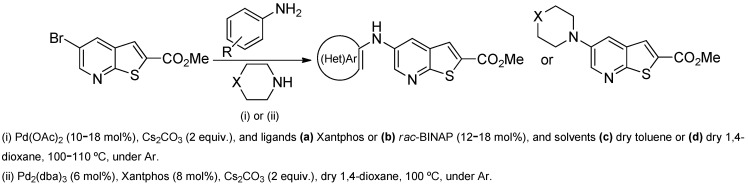			
Compounds	Reaction Conditions	Compounds	Reaction Conditions
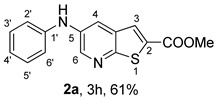	(i**bc**)	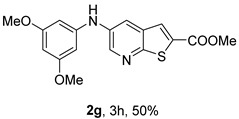	(i**bc**)
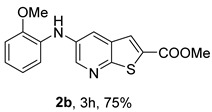	(i**bc**)	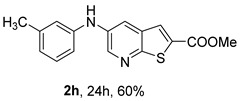	(i**bc**)
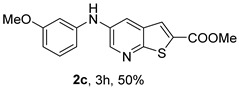	(i**bc**)	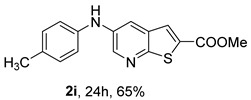	(i**bc**)
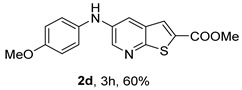	(i**bc**)	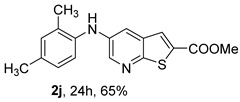	(i**bc**)
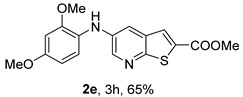	(i**bc**)	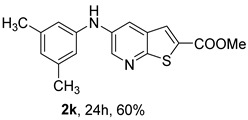	(i**bc**)
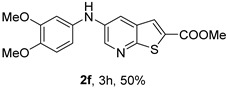	(i**bc**)	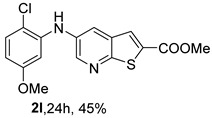	(i**bc**)
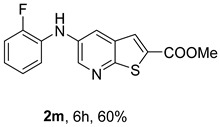	(i**ac**)	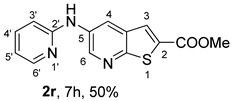	(i**ad**)
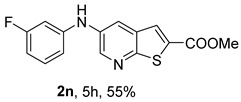	(i**ac**)	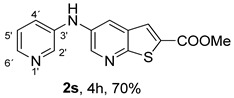	(i**ad**)
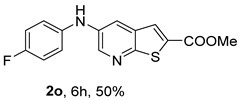	(i**ac**)	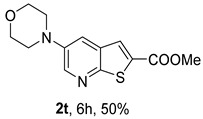	(i**bc**) *
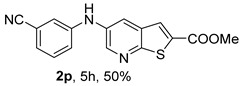	(i**ac**)	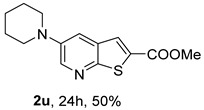	(ii) **
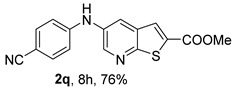	(i**ac**)		

* An attempt with (i**ac**) conditions did not afford **2t**. ** Attempts with (i**ac**) and (i**bc**) conditions did not afford **2u**.

**Table 2:** Antiparasitic activity against *T. brucei* and *L. infantum* and cytotoxicity in THP-1-derived macrophage cells for compounds **2a**–**2q**.

Compound	*T. brucei*			*L. infantum* Promastigotes			*L. infantum* Amastigotes	THP-1
	% of activity ± ST.DEV (single dose 10 μM) *^a^*	IC_50_ μM (95%) *^b^*	SI	% of activity ± ST.DEV (single dose 10 μM) *^a^*	IC_50_ μM (95%) *^b^*	SI	% of activity ± ST.DEV (single dose 10 μM)	CC_50_ (μM)
**2a**	28 ± 2.0	N.A.	−	N.A.	N.A.	−	N.A.	>100
**2b**	N.A.	N.A.	−	N.A.	N.A.	−	N.A.	>100
**2c**	37 ± 4.0	11.42 (10.8–12.2)	>8.76	N.A.	N.A.	−	N.A.	>100
**2d**	38 ± 2.0	14.33 (11.6–18.8)	>6.98	N.A.	N.A.	−	N.A.	>100
**2e**	N.A.	N.A.	−	37 ± 12	11.21 (10.3–12.4)	>8.92	N.A.	>100
**2f**	47 ± 4.0	9.66 (8.49–10.9)	>10.35	N.A.	N.A.	−	N.A.	>100
**2g**	59 ± 1.0	7.88 (6.64–9.28)	>12.70	N.A.	N.A.	−	N.A.	>100
**2h**	N.A.	N.A.	−	55 ± 30	8.39 (5.34–15.3)	>11.90	N.A.	>100
**2i**	47 ± 17	14.76 (10.4–26.4)	>6.78	56 ± 29	6.54 (3.91–11.1)	>15.30	N.A.	>100
**2j**	41 ± 19	11.03 (7.77–16.7)	>9.06	63 ± 28	4.46 (5.53–10.2)	>22.40	N.A.	>100
**2k**	32 ± 1.0	12.97 (9.30–19.1)	>7.71	57 ± 32	8.30 (5.86–12.3)	>12.04	N.A.	>100
**2l**	N.A.	N.A.	−	N.A.	N.A.	−	N.A.	>50
**2m**	N.A.	N.A.	−	N.A.	N.A.	−	N.A.	>100
**2n**	39 ± 5.0	15.15 (13.1–18.1)	>6.43	38 ± 35	N.A.	−	N.A.	>100
**2o**	N.A.	N.A.	−	N.A.	N.A.	−	N.A.	>100
**2p**	N.A.	N.A.	−	65 ± 12	8.58 (5.86–14.1)	>11.70	N.A.	>100
**2q**	N.A.	N.A.	−	68 ± 10	2.50 (1.97–3.19)	>40.00	N.A.	>100
**2r**	N.A.	N.A.	−	N.A.	N.A.	−	N.A.	>100
**2s**	N.A.	N.A.	−	N.A.	N.A.	−	N.A.	>100
**2t**	N.A.	N.A.	−	N.A.	N.A.	−	N.A.	>100
**2u**	N.A.	N.A.	−	N.A.	N.A.	−	N.A.	>100
**pentamidine**	−	0.008487 (0.00666–0.0108)	4961.71	−	−	−	−	42.11 (36.94–48.00)
**miltefosine**	−	−	−	−	9.71 (8.60–10.9)	4.72	−	45.8 (35.2–55.6)

*^a^* % of activity ± ST.DEV (single dose 10 μM) is the average percentage of target activity observed when the compound is applied at 10 μM, along with the standard deviation from replicate measurements*. ^b^* Confidence Interval of 95% (µM) for the mean curve of all independent assays. N.A. = No Activity. Pentamidine and miltefosine were used as reference drugs and internal controls for *T. brucei* and *L. infantum*, respectively.

## Data Availability

The data presented in this study are available in the article and in the Supplementary Materials.

## Supplementary Materials

The following supporting information can be downloaded at: https://www.mdpi.com/article/10.3390/molecules31081313/s1, and contains synthesis of the compound **1**, ^1^H and ^13^C NMR spectra of compounds **1**, **2a**–**2u**, and a table of antiparasitic activity at a single dose 20 μM, against *T*. *brucei* and *L. infantum* promastigotes for compounds **2a**–**2u** are available online. Scheme S1: Synthesis of methyl-5-bromothieno[2,3-*b*]pyridine-2-carboxylate (**1**). Table S1: Antiparasitic activity (single dose 20 μM) against *T. brucei* and *L. infantum* promastigotes for compounds **2a**–**2u**.
