# Supplementary material for: Synthesis, Antiparasitic Activity and Substituent Effects of Methyl 5-(Hetero)aryl or Alicyclicaminothieno[2,3-b]pyridine-2-carboxylates
Source: Molecules. 2026 Apr 17;31(8):1313. doi: 10.3390/molecules31081313 (PMC13118280; doi:10.3390/molecules31081313)
Supplement: Supplementary file 1 [file molecules-31-01313-s001.zip › molecules-4210839-supplementary.pdf]

Supplementary Materials

Synthesis, Antiparasitic Activity and Substituent Effects of Methyl 5-(Hetero)aryl or Alicyclicaminothieno[2,3-*b*]pyridine-2-carboxylates

Francisco Ribeiro [1], Juliana P. Sousa [1], Nuno Santarém [2,3], Joana Tavares [2,4], Anabela Cordeiro-da-Silva [2,3], Maria-João R. P. Queiroz [1*]

1 Centro de Química, Universidade do Minho (CQUM), Campus de Gualtar, 4710-057 Braga, Portugal; b15328@uminho.pt , pg52275@uminho.pt

2 Host-Parasite Interactions, I3S - Instituto de Investigação e Inovação em Saúde, Universidade do Porto, Rua Alfredo Allen, 208, 4200-135 Porto, Portugal; santarem@i3s.up.pt , cordeiro@i3s.up.pt, jtavares@i3s.up.pt

3 Laboratório de Microbiologia, Departamento de Ciências Biológicas, Faculdade de Farmácia, Universidade do Porto, Rua de Jorge Viterbo Ferreira, 228, 4050-313 Porto, Portugal; cordeiro@ff.up.pt, nsantarem@ff.up.pt

4 Departmento de Biologia Molecular, ICBAS – Instituto de Ciências Biomédicas Abel Salazar, Universidade do Porto, Rua de Jorge Viterbo Ferreira, 228, 4050-313 Porto, Portugal.; jtavares@i3s.up.pt

* Correspondence: mjrpq@quimica.uminho.pt.

Synthesis of the precursor methyl-5-bromothieno[2,3-*b*]pyridine-2-carboxylate 1 5
1. **NMR spectra of compound 1** 6
   ^{1}H NMR (DMSO- d_6, 400 MHz) of compound 1 6
   ^{13}C NMR (DMSO- d_6, 100.6 MHz) of compound 1 6
2. **NMR spectra of compound 2a** 7
   ^{1}H NMR (DMSO- d_6, 400 MHz) of compound 2a 7
   ^{13}C NMR (DMSO- d_6, 100.6 MHz) of compound 2a 7
3. **NMR spectra of compound 2b** 8
   ^{1}H NMR (DMSO- d_6, 400 MHz) of compound 2b 8
   ^{13}C NMR (DMSO- d_6, 100.6 MHz) of compound 2b 8
4. **NMR spectra of compound 2c** 9
   ^{1}H NMR (DMSO- d_6, 400 MHz) of compound 2c 9
   ^{13}C NMR (DMSO- d_6, 100.6 MHz) of compound 2c 9
5. **NMR spectra of compound 2d** 10
   ^{1}H NMR (DMSO- d_6, 400 MHz) of compound 2d 10
   ^{13}C NMR (DMSO- d_6, 100.6 MHz) of compound 2d 10
6. **NMR spectra of compound 2e** 11
   ^{1}H NMR (DMSO- d_6, 400 MHz) of compound 2e 11
   ^{13}C NMR (DMSO- d_6, 100.6 MHz) of compound 2e 11
7. **NMR spectra of compound 2f** 12
   ^{1}H NMR (DMSO- d_6, 400 MHz) of compound 2f 12
   ^{13}C NMR (DMSO- d_6, 100.6 MHz) of compound 2f 12
8. **NMR spectra of compound 2g** 13
   ^{1}H NMR (DMSO- d_6, 400 MHz) of compound 2g 13
   ^{13}C NMR (DMSO- d_6, 100.6 MHz) of compound 2g 13
9. **NMR spectra of compound 2h** 14
   ^{1}H NMR (DMSO- d_6, 400 MHz) of compound 2h 14
   ^{13}C NMR (DMSO-d_6, 100.6 MHz) of compound 2h 14

10. NMR spectra of compound 2i 15
^{1}H NMR (DMSO- d_6, 400 MHz) of compound 2i 15
^{13}C NMR (DMSO-d_6, 100.6 MHz) of compound 2i 15
11. NMR spectra of compound 2j 16
^{1}H NMR (DMSO- d_6, 400 MHz) of compound 2j 16
^{13}C NMR (DMSO-d_6, 100.6 MHz) of compound 2j 16
12. NMR spectra of compound 2k 17
^{1}H NMR (DMSO- d_6, 400 MHz) of compound 2k 17
^{13}C NMR (DMSO-d_6, 100.6 MHz) of compound 2k 17
13. NMR spectra of compound 2l 18
^{1}H NMR (DMSO- d_6, 400 MHz) of compound 2l 18
^{13}C NMR (DMSO-d_6, 100.6 MHz) of compound 2l 18
14. NMR spectra of compound 2m 19
^{1}H NMR (DMSO- d_6, 400 MHz) of compound 2m 19
^{13}C NMR (DMSO-d_6, 100.6 MHz) of compound 2m 19
15. NMR spectra of compound 2n 20
^{1}H NMR (DMSO- d_6, 400 MHz) of compound 2n 20
^{13}C NMR (DMSO-d_6, 100.6 MHz) of compound 2n 20
16. NMR spectra of compound 2o 21
^{1}H NMR (DMSO- d_6, 400 MHz) of compound 2o 21
^{13}C NMR (DMSO-d_6, 100.6 MHz) of compound 2o 21
HMBC spectrum of compound 2o 22
17. NMR spectra of compound 2p 23
^{1}H NMR (DMSO- d_6, 400 MHz) of compound 2p 23
^{13}C NMR (DMSO-d_6, 100.6 MHz) of compound 2p 23
18. NMR spectra of compound 2q 24
^{1}H NMR (DMSO- d_6, 400 MHz) of compound 2q 24
^{13}C NMR (DMSO-d_6, 100.6 MHz) of compound 2q 24
19. NMR spectra of compound 2r 25

^{1}H NMR (DMSO- *d_6*, 400 MHz) of compound 2r 25

^{13}C NMR (DMSO-*d_6*, 100.6 MHz) of compound 2r 25

20. NMR spectra of compound 2s 26

^{1}H NMR (DMSO- *d_6*, 400 MHz) of compound 2s 26

^{13}C NMR (DMSO-*d_6*, 100.6 MHz) of compound 2s 26

^{13}C NMR (DMSO-*d_6*, 100.6 MHz) of compound 2s 26

21. NMR spectra of compound 2t 27

^{1}H NMR (DMSO- *d_6*, 400 MHz) of compound 2t 27

^{13}C NMR (DMSO-*d_6*, 100.6 MHz) of compound 2t 27

22. NMR spectra of compound 2u 28

^{1}H NMR (DMSO- *d_6*, 400 MHz) of compound 2u 28

^{13}C NMR (DMSO-*d_6*, 100.6 MHz) of compound 2u 28

Table S1. Antiparasitic activity (single dose 20 μM) against *T. brucei* and *L. infantum* promastigotes for compounds **2a-2u** 29

Synthesis of the precursor methyl-5-bromothieno[2,3-*b*]pyridine-2-carboxylate 1

The methyl 5-bromothieno[2,3-*b*]pyridine-2-carboxylate (**1**), was synthesized by reacting 5-bromo-2-fluoronicotinaldehyde with methyl thioglycolate under basic conditions in DMF at 80 °C for 3 h (Scheme S1).

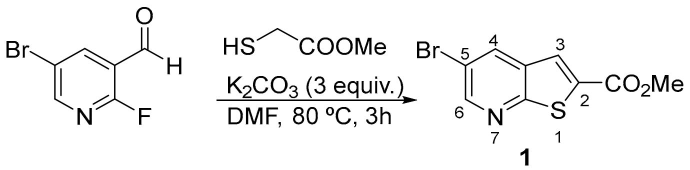

Scheme S1. Synthesis of methyl-5-bromothieno[2,3-*b*]pyridine-2-carboxylate (**1**)

To 5-bromo-2-fluoronicotinaldehyde (0.500 g, 2.45 mmol) in DMF (10 mL), anhydrous K_2CO_3 (1.02 g 7.35 mmol) and methyl thioglycolate (0.329 mL, 3.68 mmol) were added. The reaction was left stirring at 80 ^{o}C. The reaction was completed after 3 hours, monitored by TLC. After cooling, the reaction mixture was poured into ice, stirred, and a precipitate came out. This was filtered under vacuum, washed with water, and dried in the oven at 50 ^{o}C, to give compound 1 as a white solid (0.480 g, 71%), m.p. 162.0-162.2 ^{o}C. ^{1}H NMR (400 MHz, DMSO-d_6) δ = 3.91 (3H, s, OCH_3), 8.14 (1H, s, 3-H), 8.72 (1H, d, J = 2.4 Hz, 4-H), 8.83 (1H, d, J = 2.4 Hz, 6-H) ppm. ^{13}C (100.6 MHz, DMSO-d_6) δ = 53.1 (OCH_3), 116.9 (C), 128.2 (3-CH), 133.7 (C), 134.1 (C), 136.1 (4-CH), 150.2 (6-CH), 160.0 (C), 161.8 (C=O) ppm. MS(ESI) $[M + H]^+$, m/z=273. HRMS (ESI/ $[M+H]^+$): calculated m/z ($[C_9H_6BrNO_2S]^+$): 271.9376, found 271.9375; (Error: -0.4 ppm).

1. NMR spectra of compound 1

¹H NMR (DMSO- d_6, 400 MHz) of compound 1

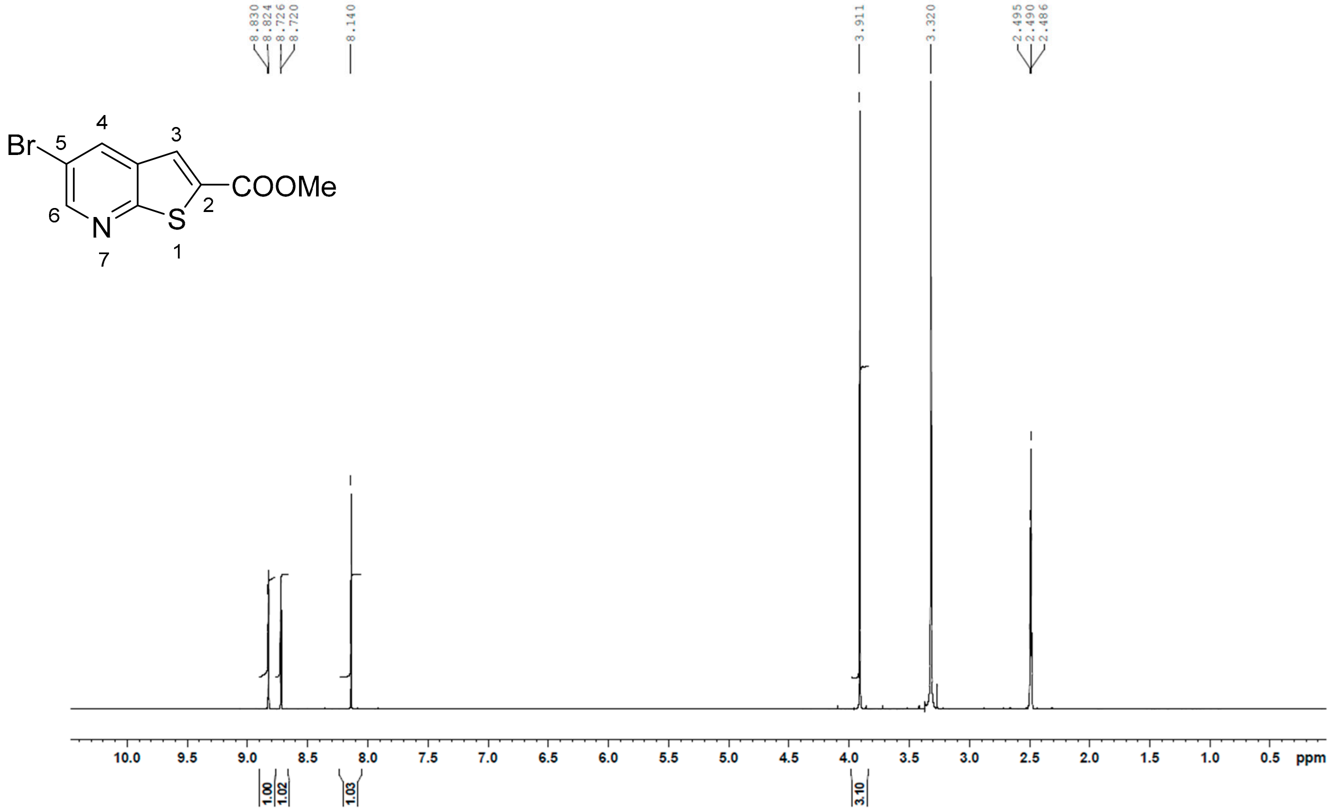

¹³C NMR (DMSO- d_6, 100.6 MHz) of compound 1

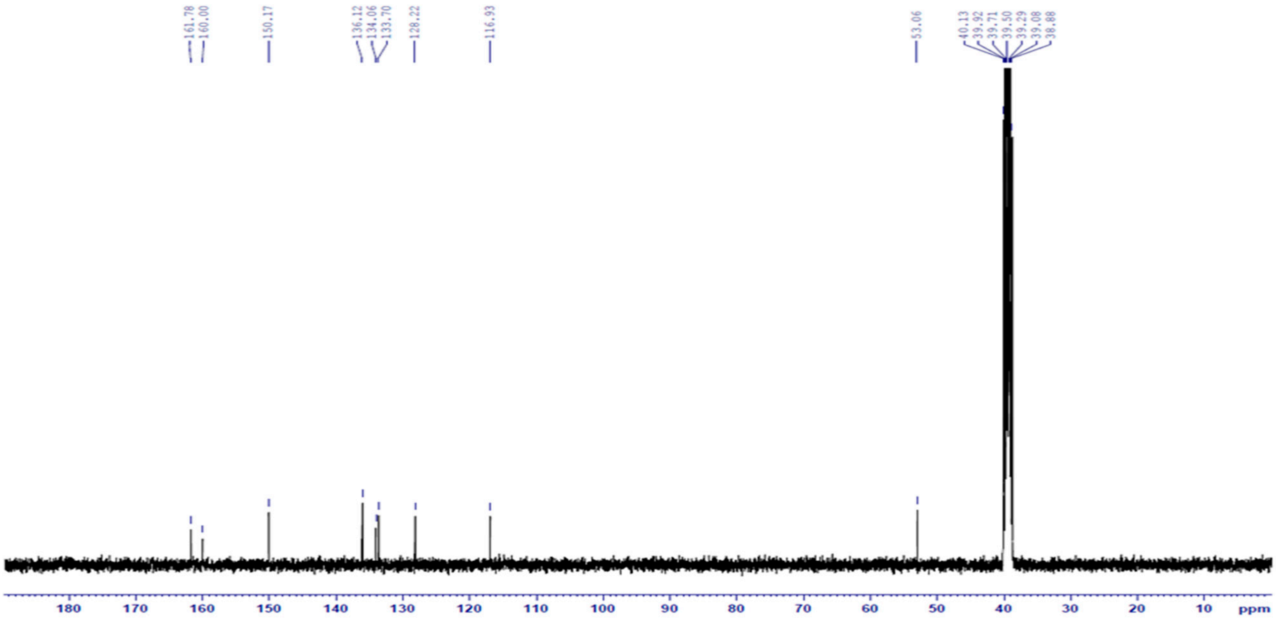

2. NMR spectra of compound 2a

^{1}H NMR (DMSO- *d*$_6$, 400 MHz) of compound 2a

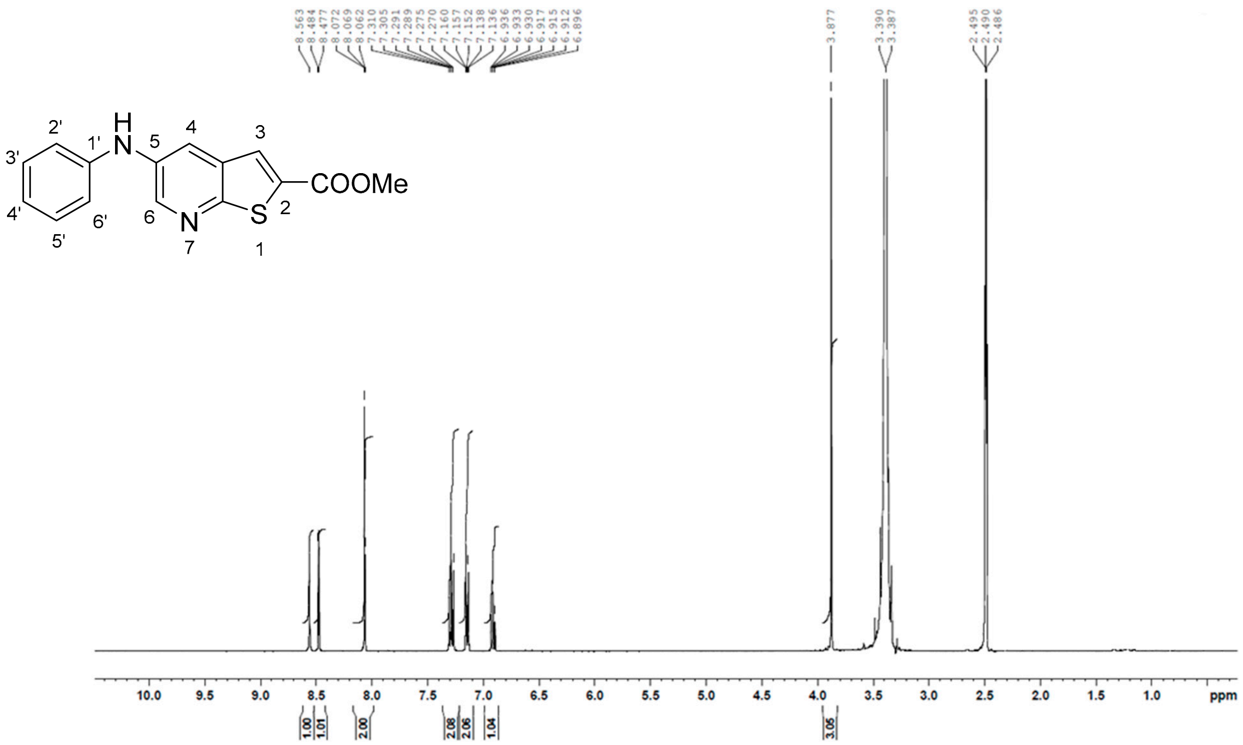

^{13}C NMR (DMSO- *d*$_6$, 100.6 MHz) of compound 2a

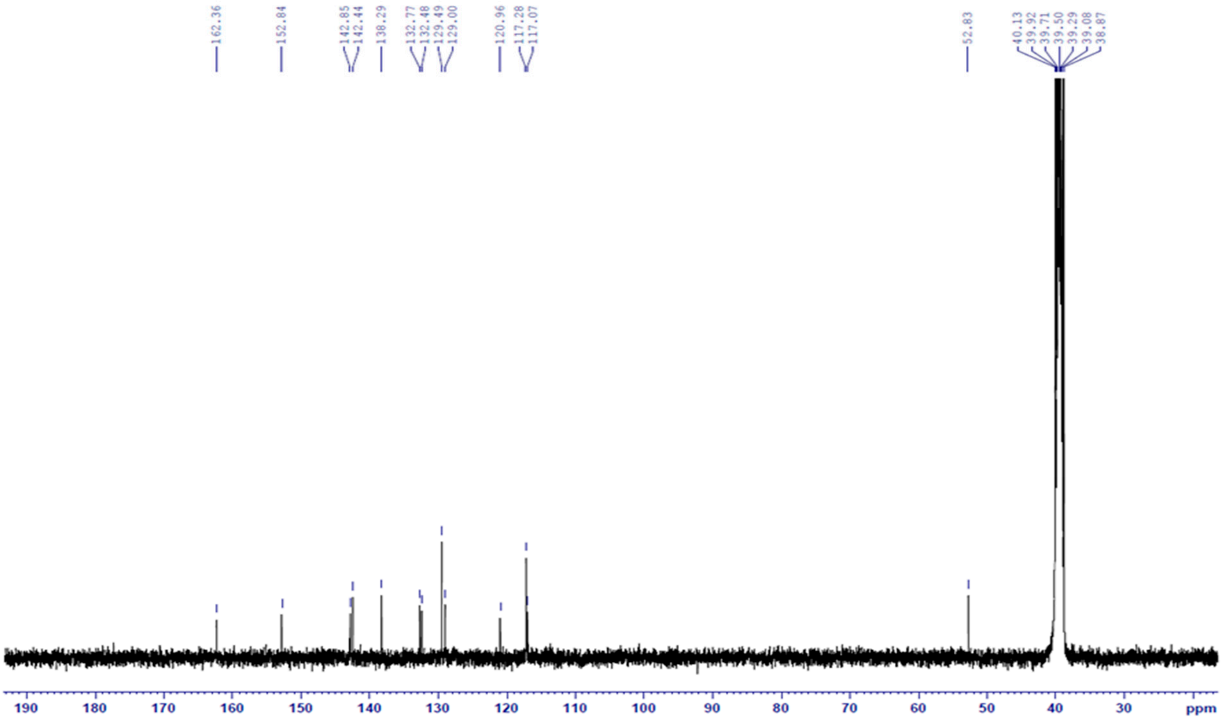

3. NMR spectra of compound 2b

^{1}H NMR (DMSO- *d*6, 400 MHz) of compound 2b

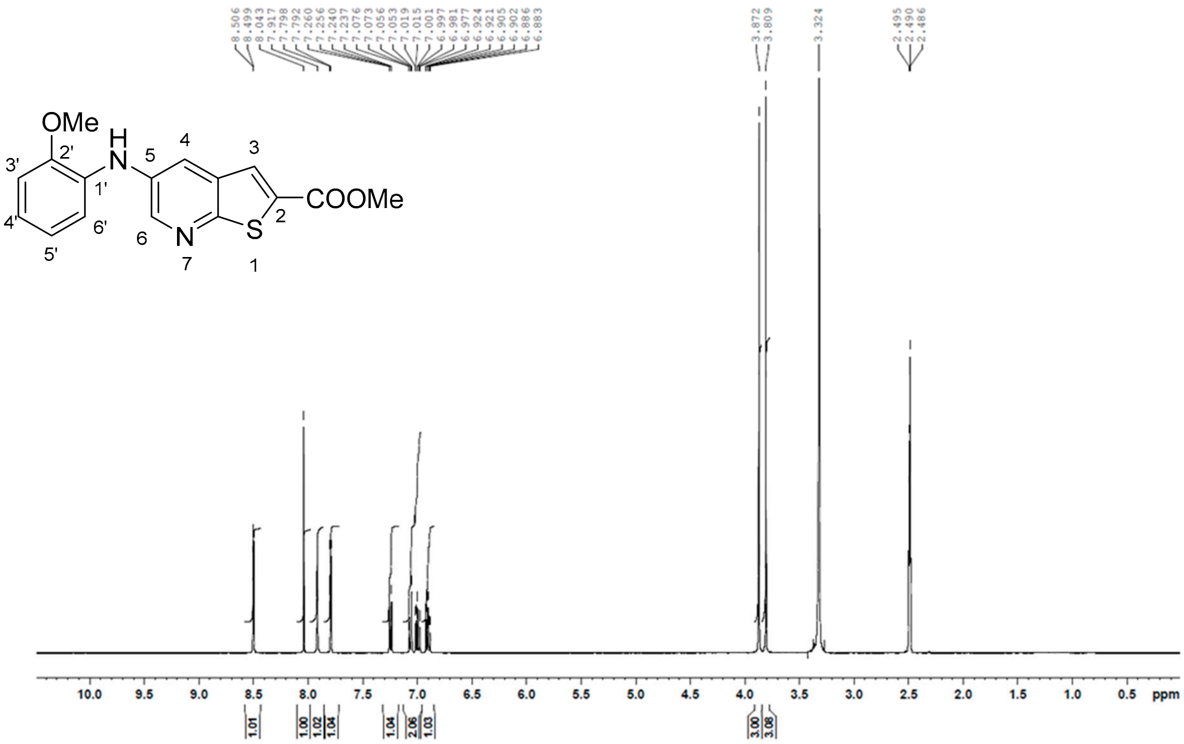

^{13}C NMR (DMSO- *d*6, 100.6 MHz) of compound 2b

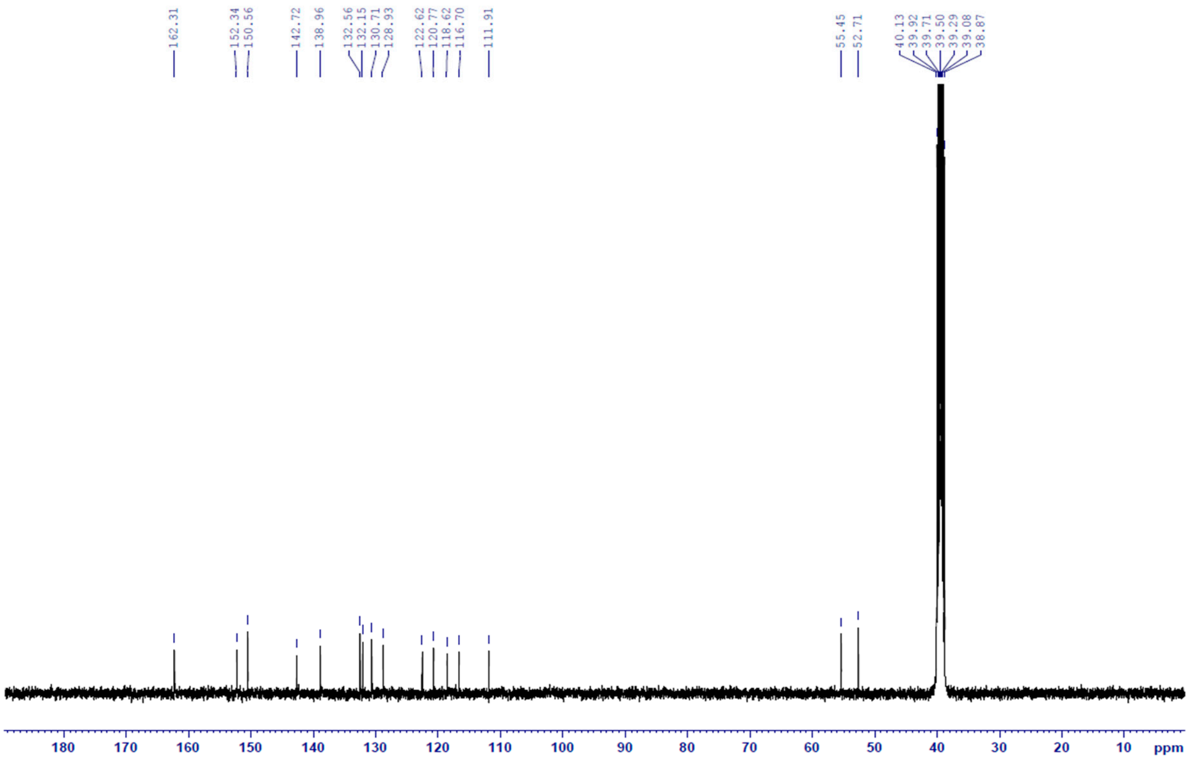

4. NMR spectra of compound 2c

^{1}H NMR (DMSO- *d*6, 400 MHz) of compound 2c

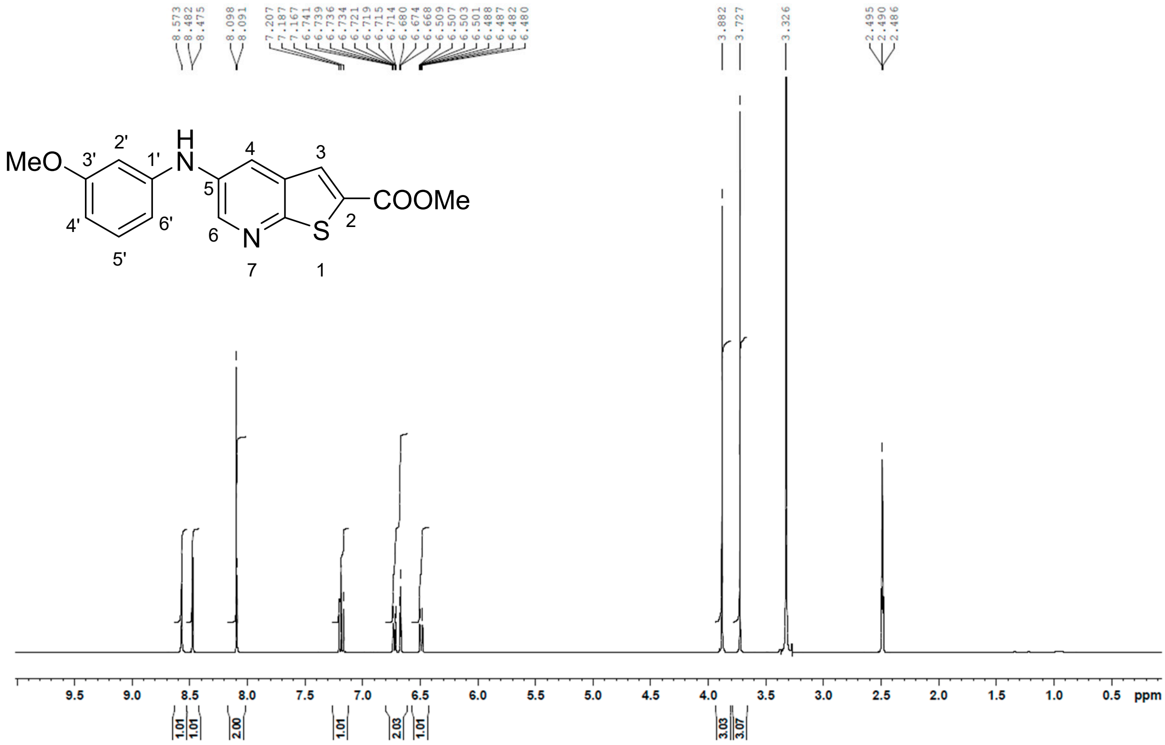

^{13}C NMR (DMSO- *d*6, 100.6 MHz) of compound 2c

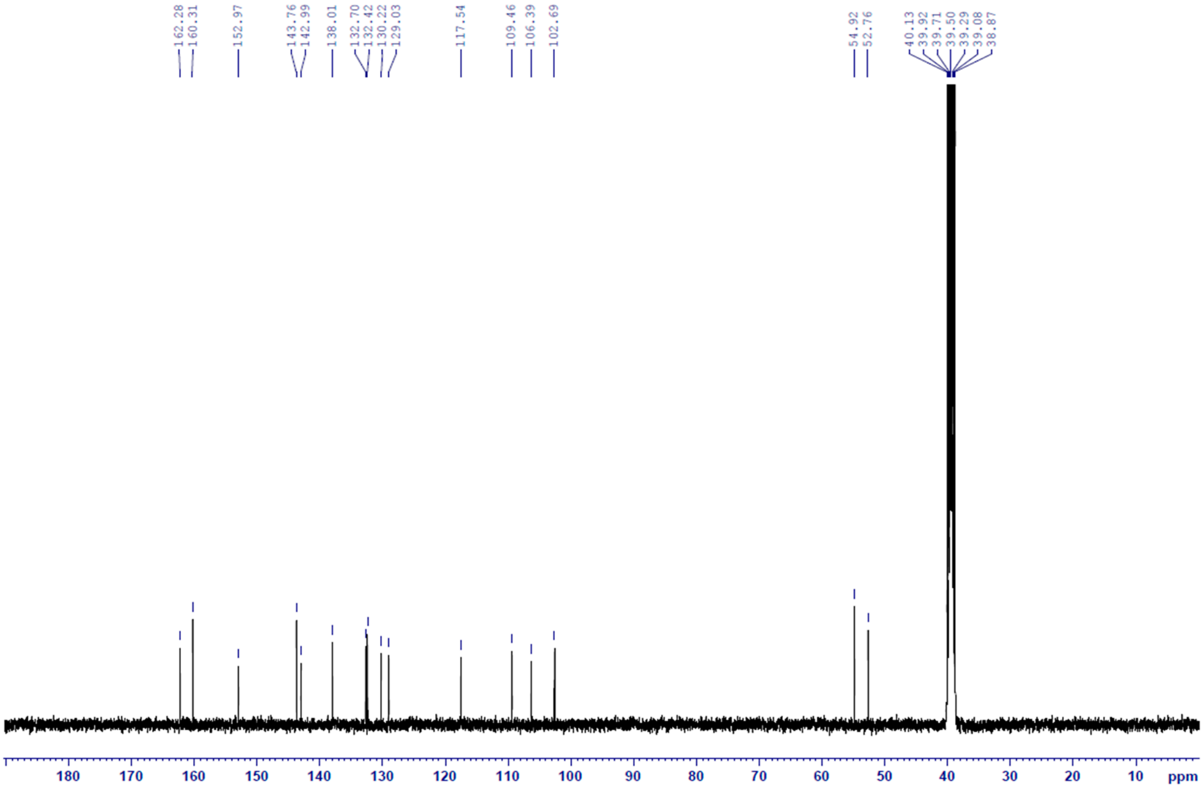

5. NMR spectra of compound 2d

^{1}H NMR (DMSO- d_6, 400 MHz) of compound 2d

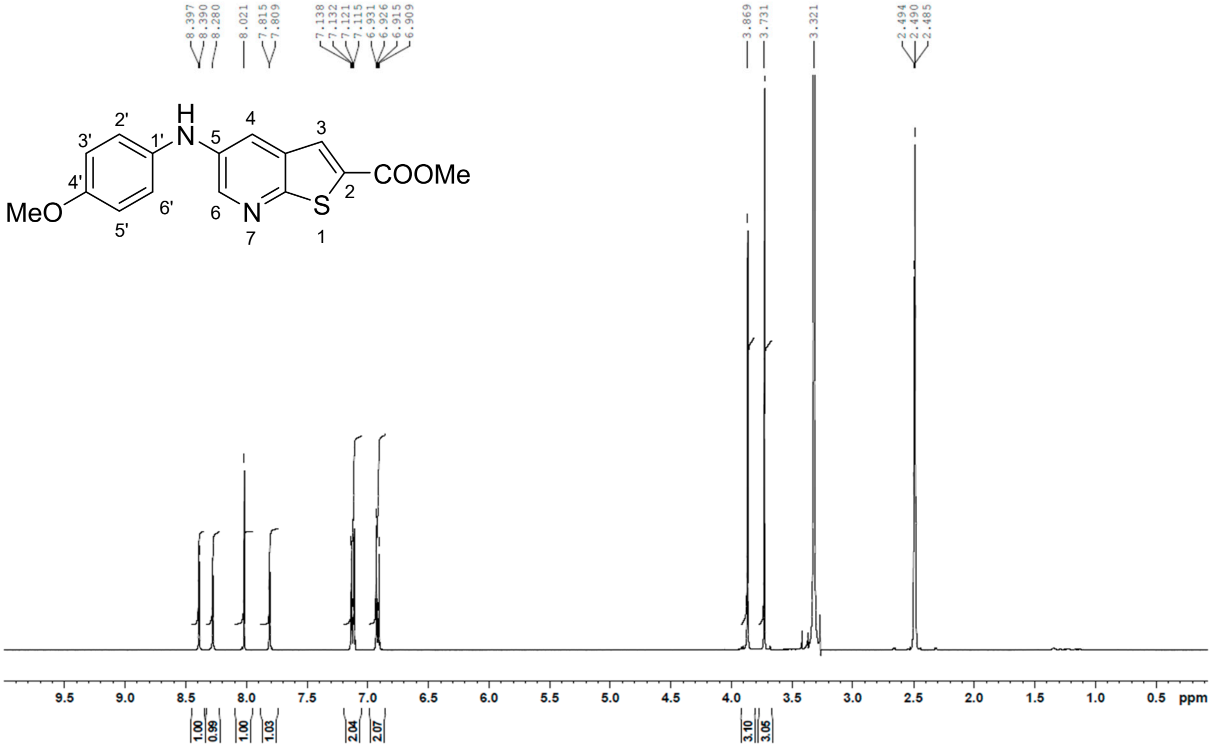

^{13}C NMR (DMSO- d_6, 100.6 MHz) of compound 2d

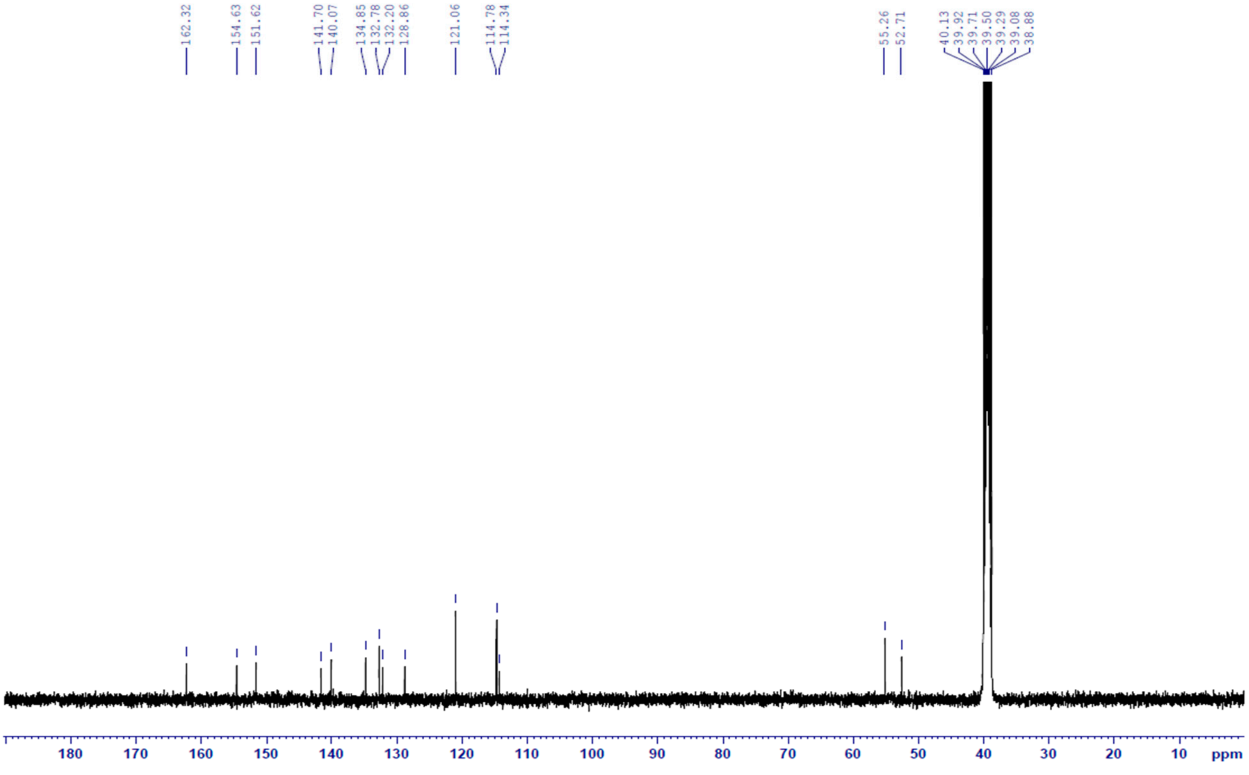

6. NMR spectra of compound 2e

^{1}H NMR (DMSO- *d*6, 400 MHz) of compound 2e

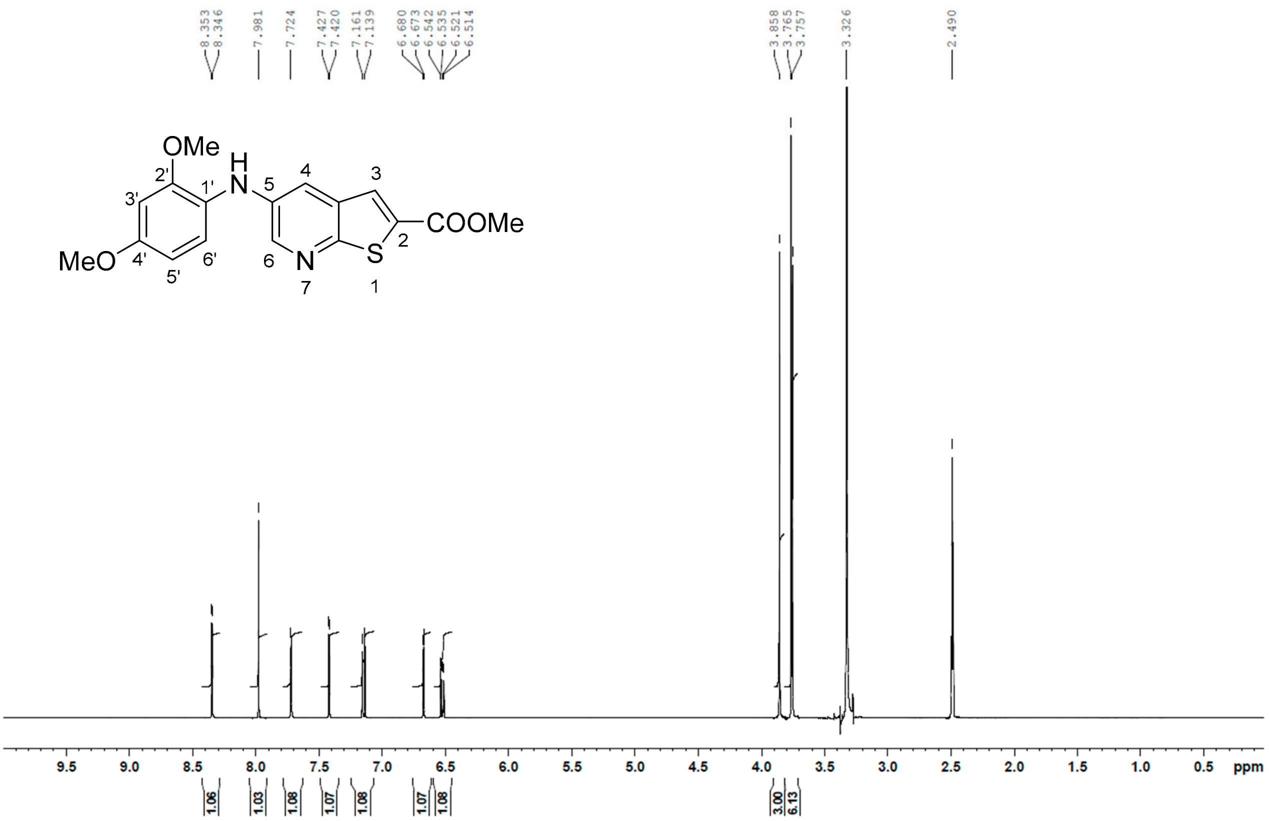

^{13}C NMR (DMSO- *d*6, 100.6 MHz) of compound 2e

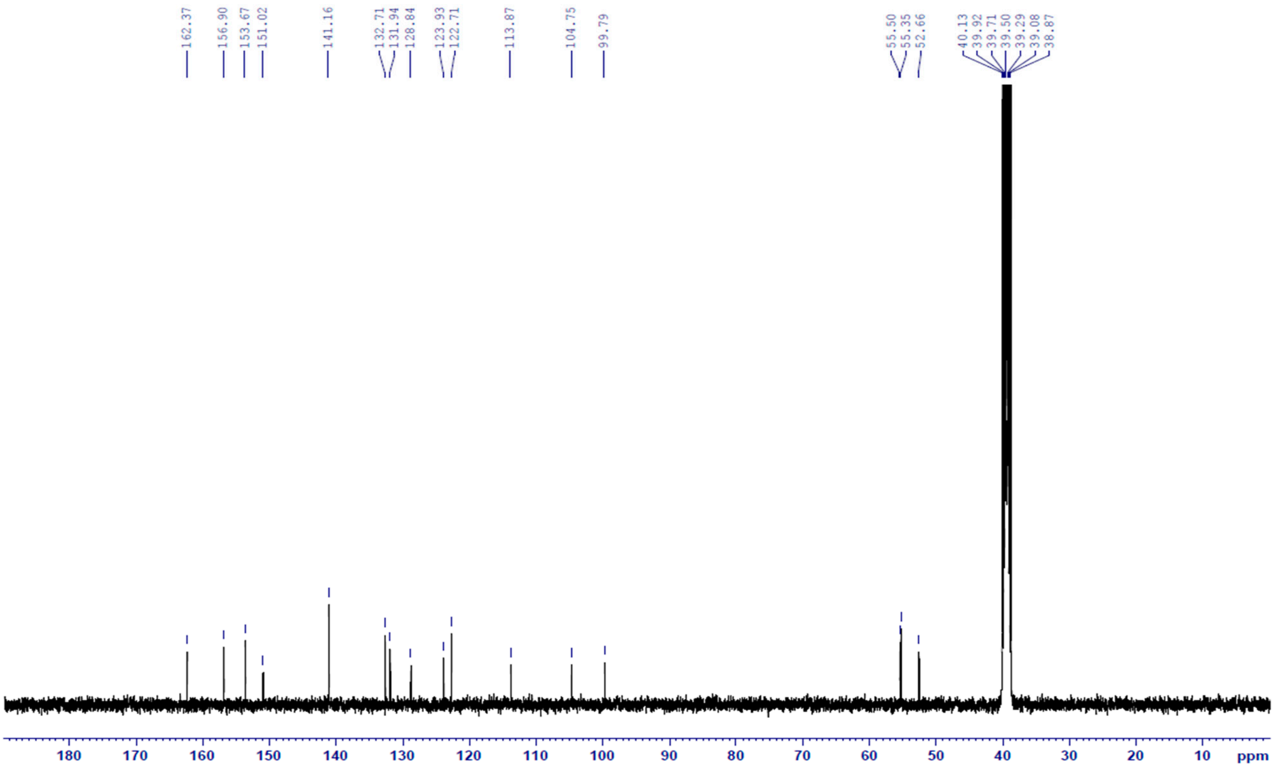

7. NMR spectra of compound 2f

^{1}H NMR (DMSO- *d*6, 400 MHz) of compound 2f

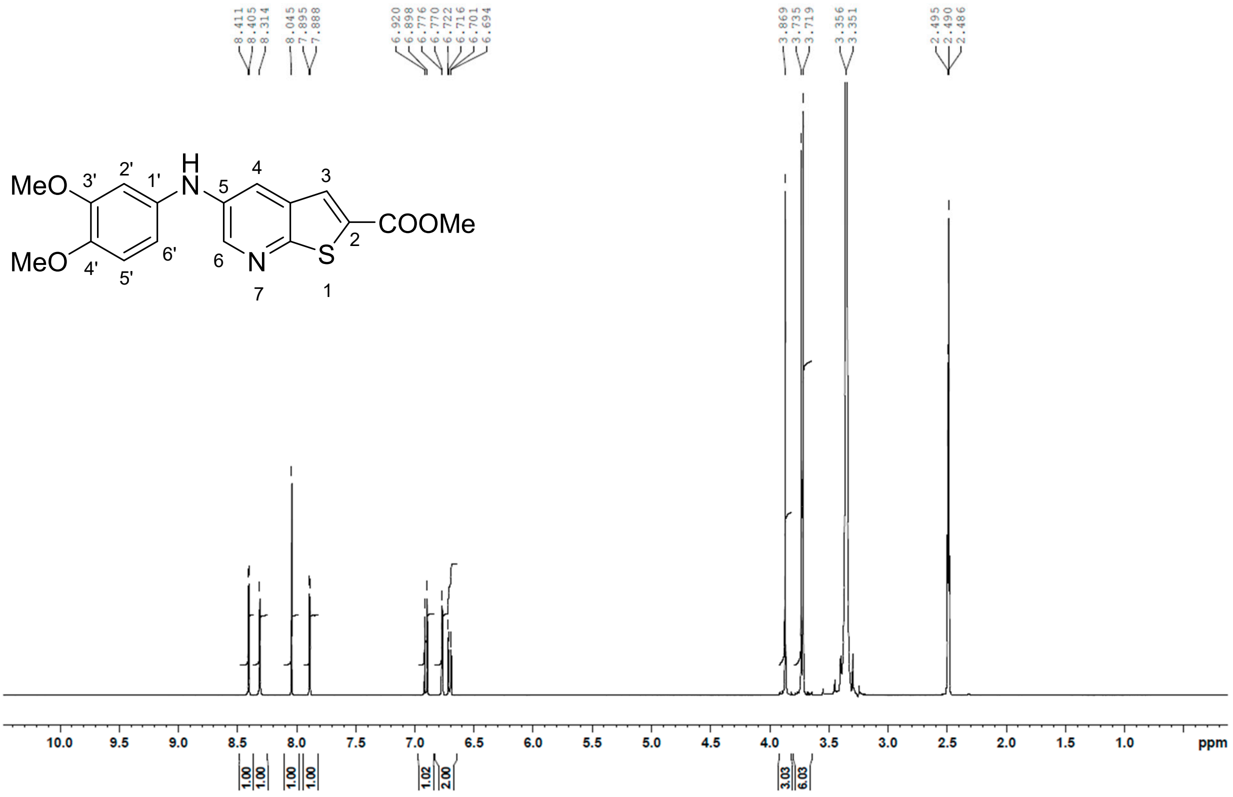

^{13}C NMR (DMSO- *d*6, 100.6 MHz) of compound 2f

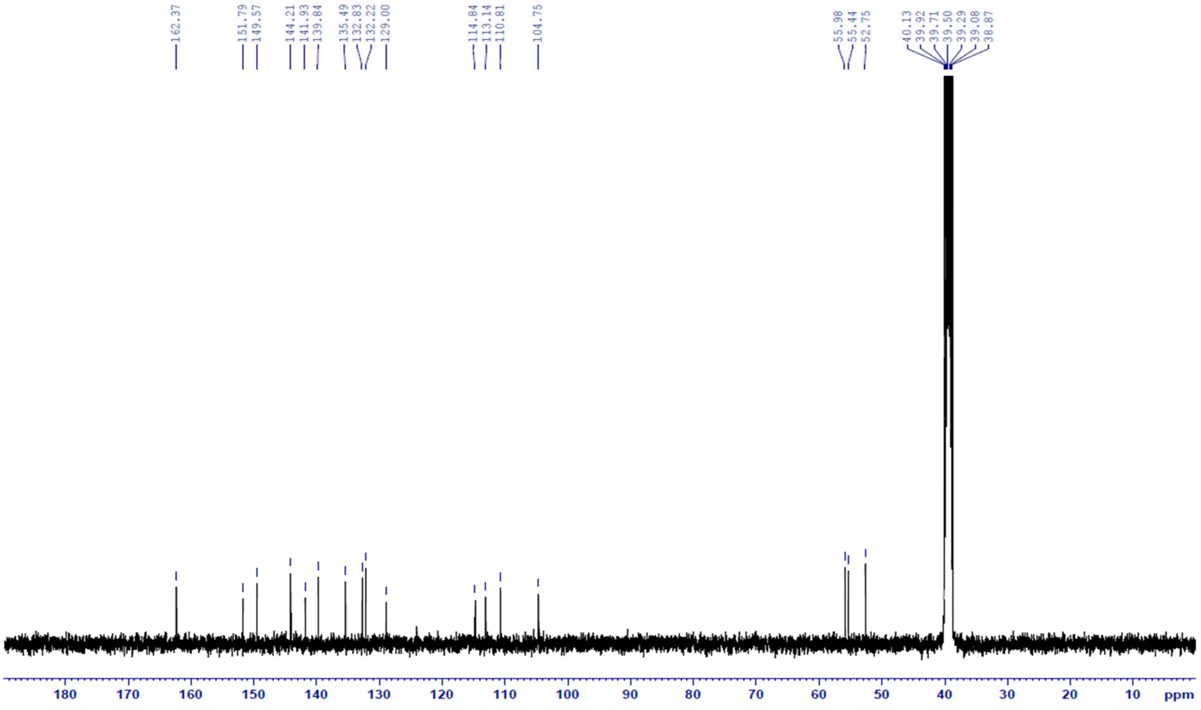

8. NMR spectra of compound 2g

^{1}H NMR (DMSO- d_6, 400 MHz) of compound 2g

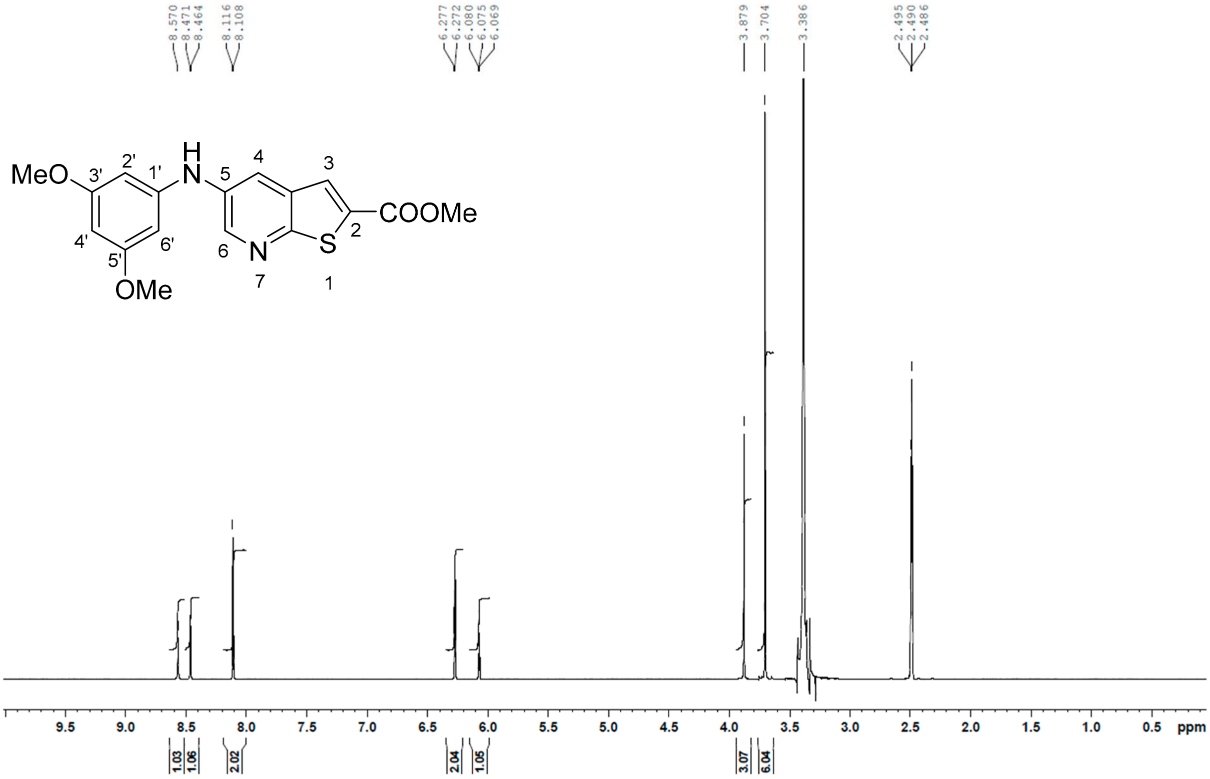

^{13}C NMR (DMSO- d_6, 100.6 MHz) of compound 2g

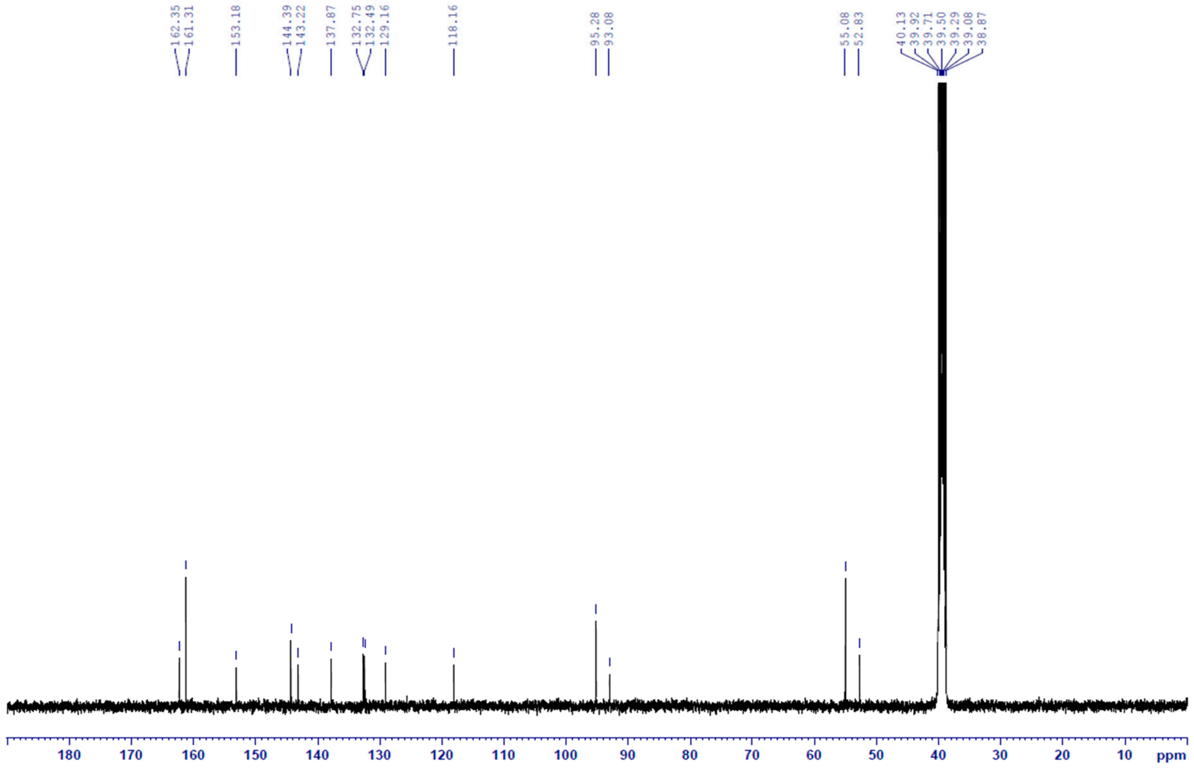

9. NMR spectra of compound 2h

^{1}H NMR (DMSO- *d*6, 400 MHz) of compound 2h

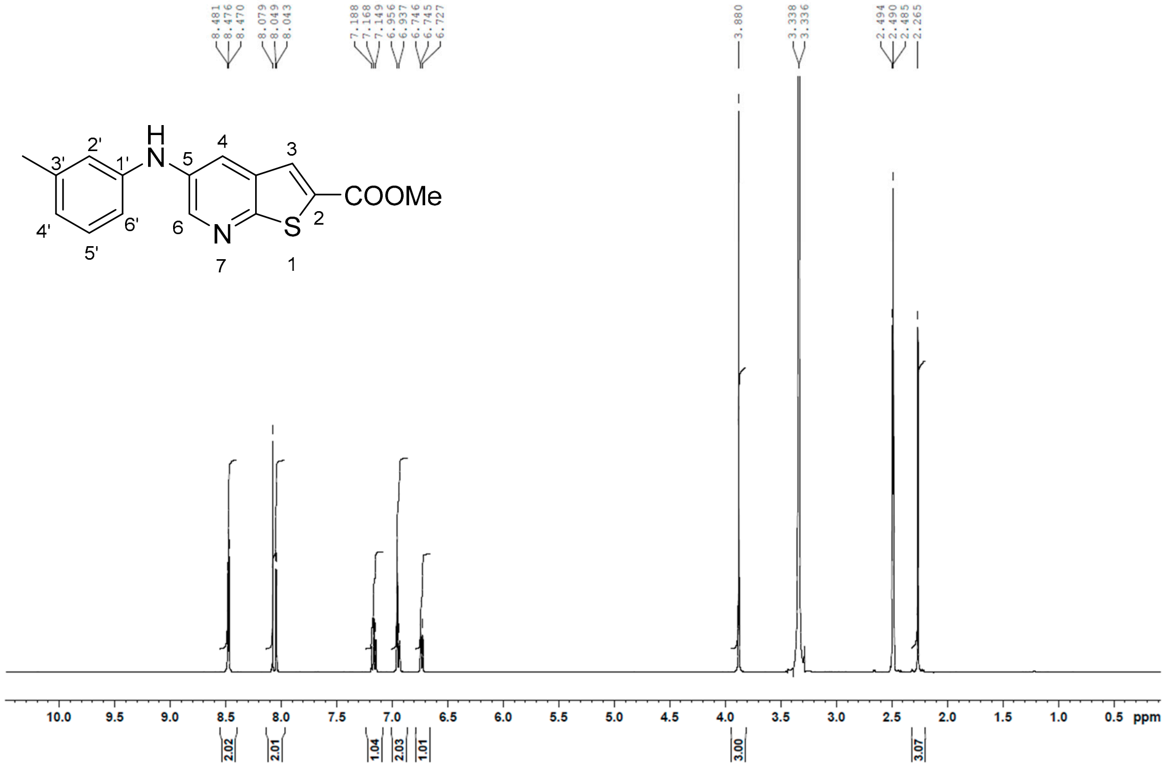

^{13}C NMR (DMSO-*d*6, 100.6 MHz) of compound 2h

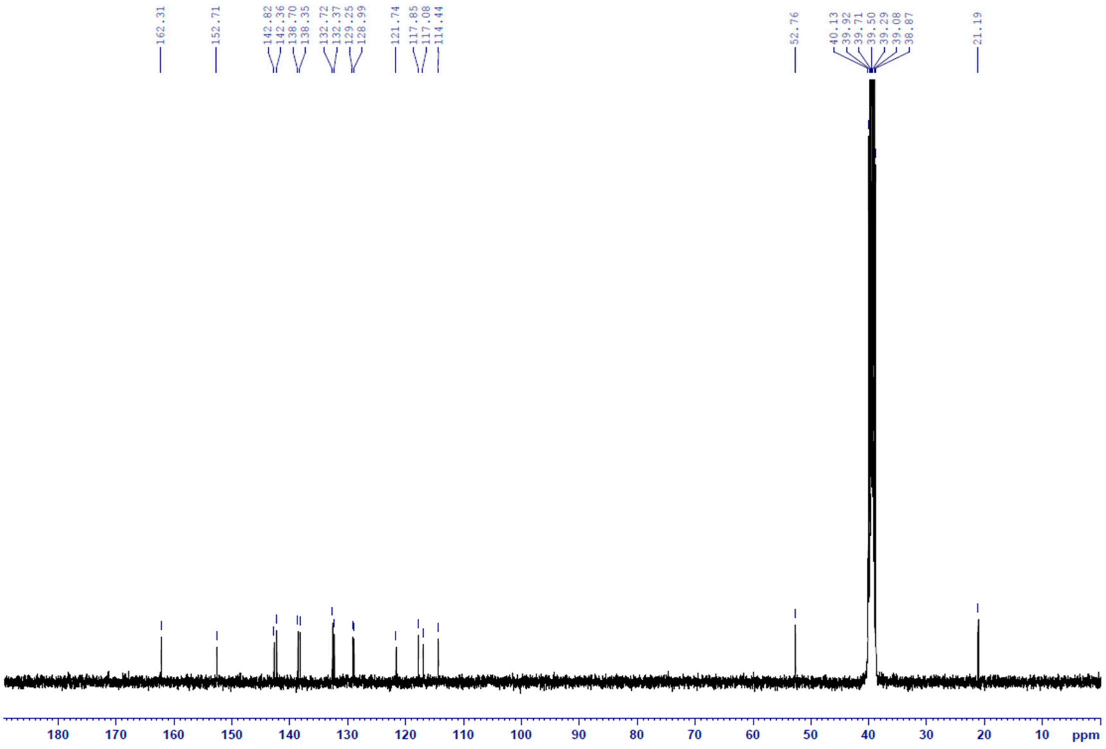

10.NMR spectra of compound 2i

^{1}H NMR (DMSO- d_6, 400 MHz) of compound 2i

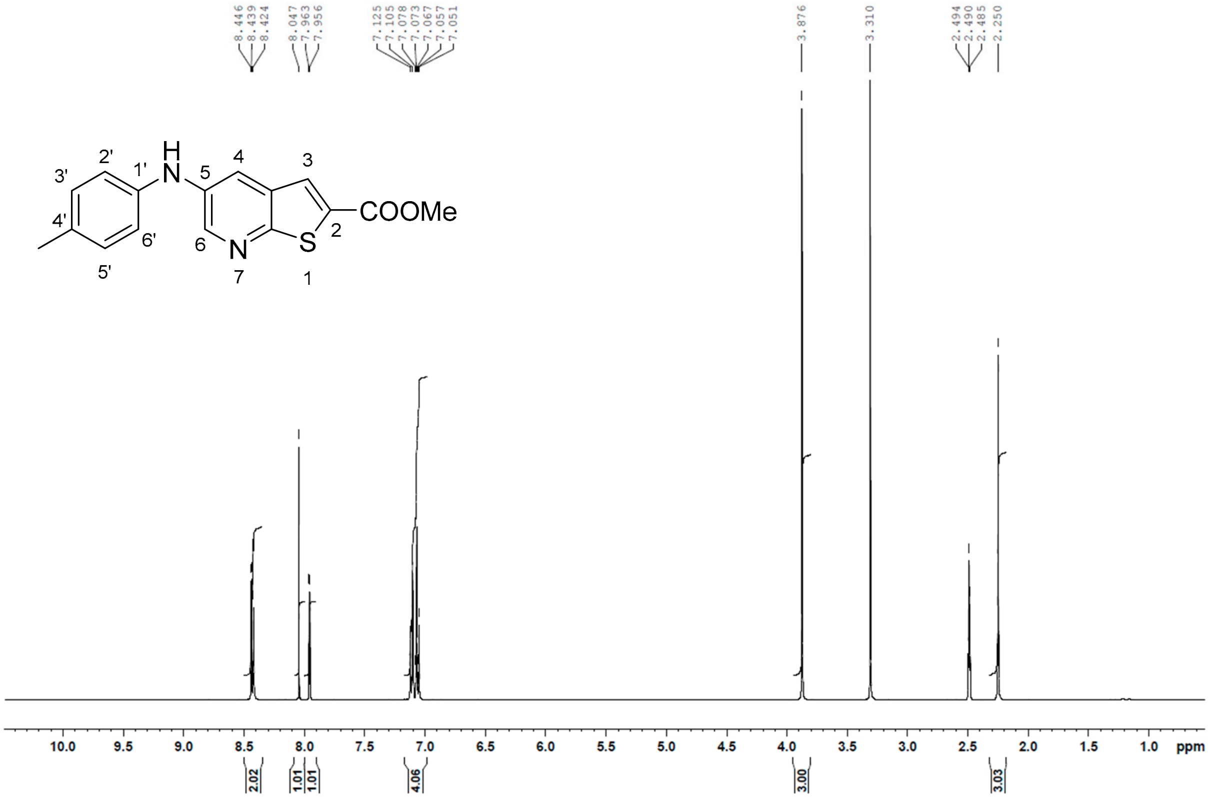

^{13}C NMR (DMSO-d_6, 100.6 MHz) of compound 2i

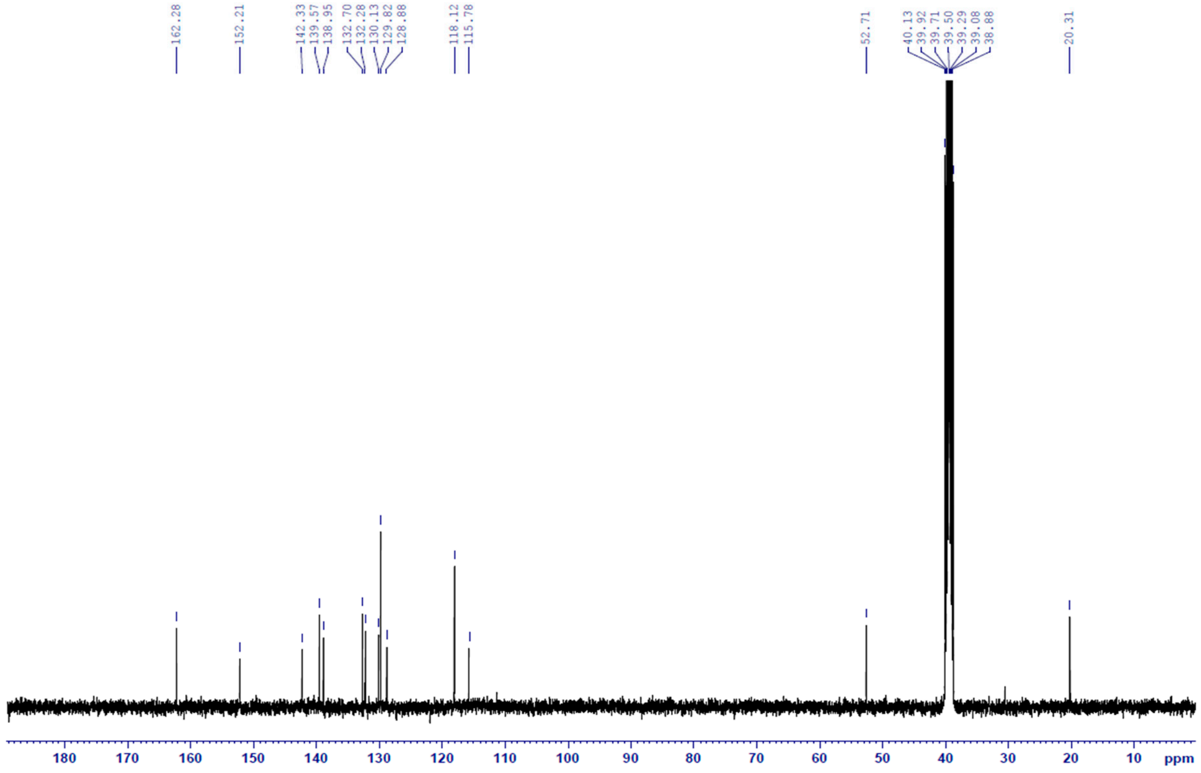

11.NMR spectra of compound 2j

^{1}H NMR (DMSO- d_6, 400 MHz) of compound 2j

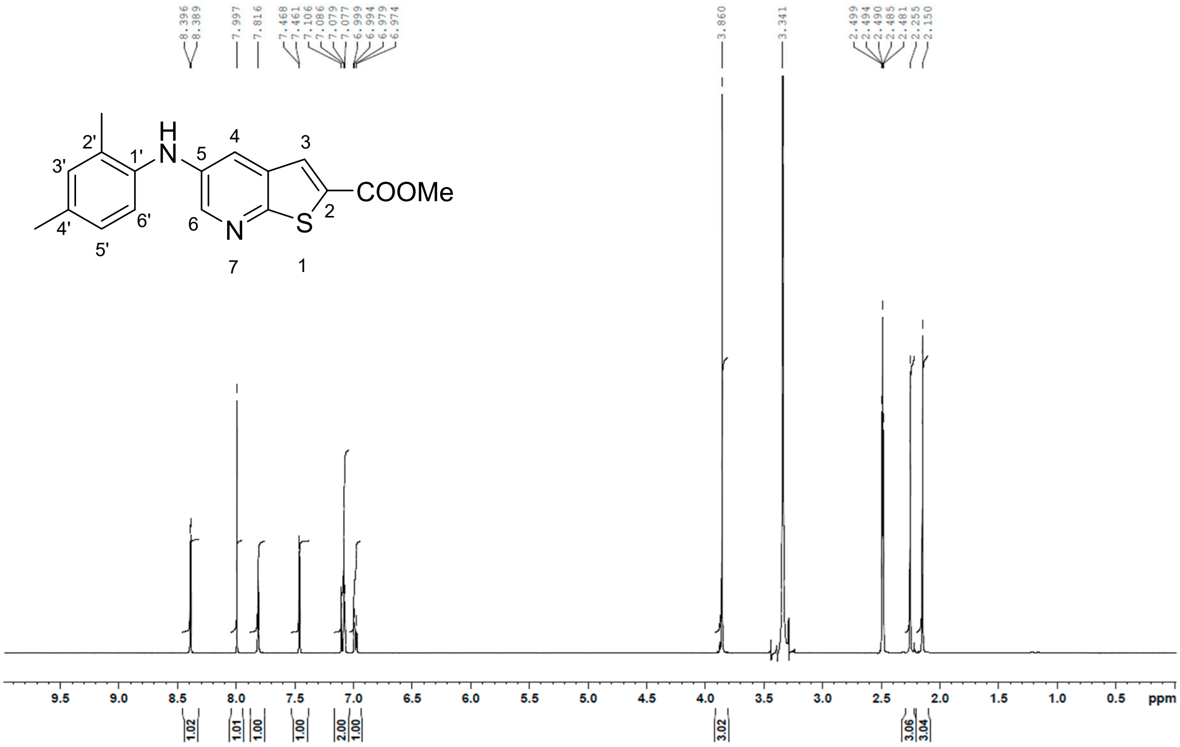

^{13}C NMR (DMSO-d_6, 100.6 MHz) of compound 2j

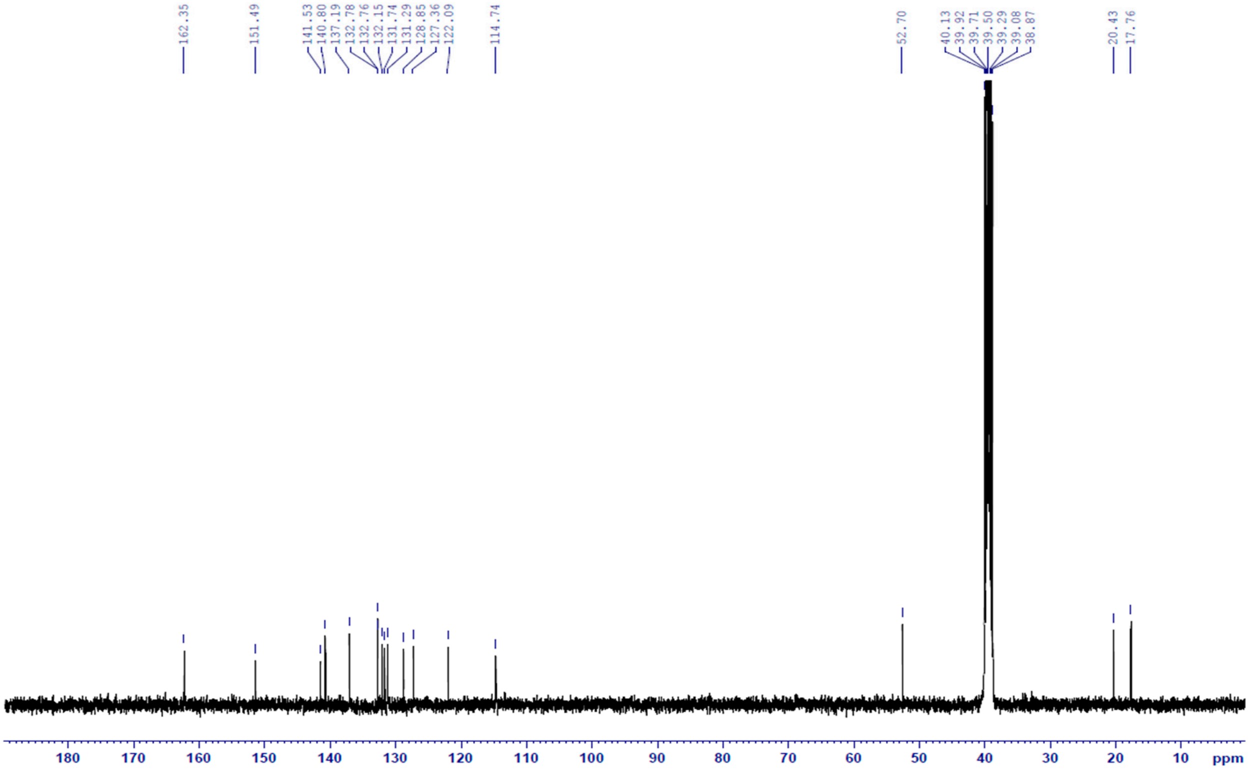

12. NMR spectra of compound 2k

^{1}H NMR (DMSO- *d*6, 400 MHz) of compound 2k

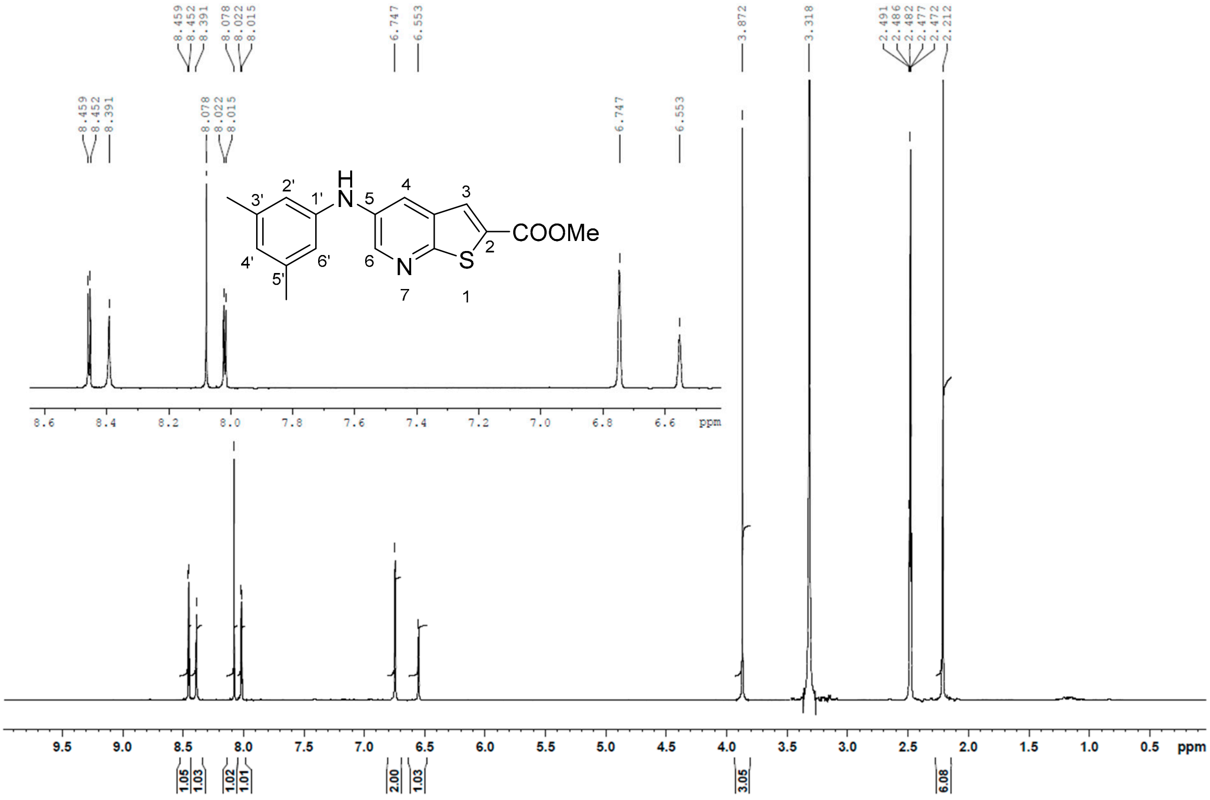

^{13}C NMR (DMSO-*d*6, 100.6 MHz) of compound 2k

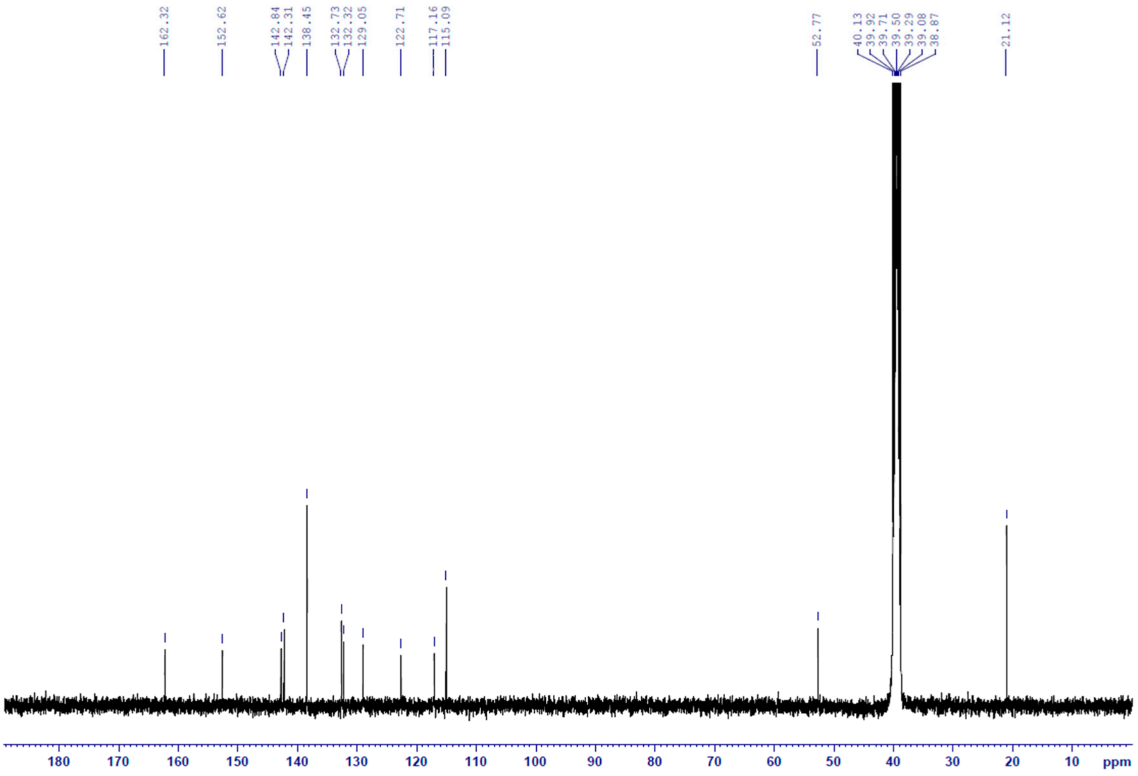

13.NMR spectra of compound 2l

^{1}H NMR (DMSO- d_6, 400 MHz) of compound 2l

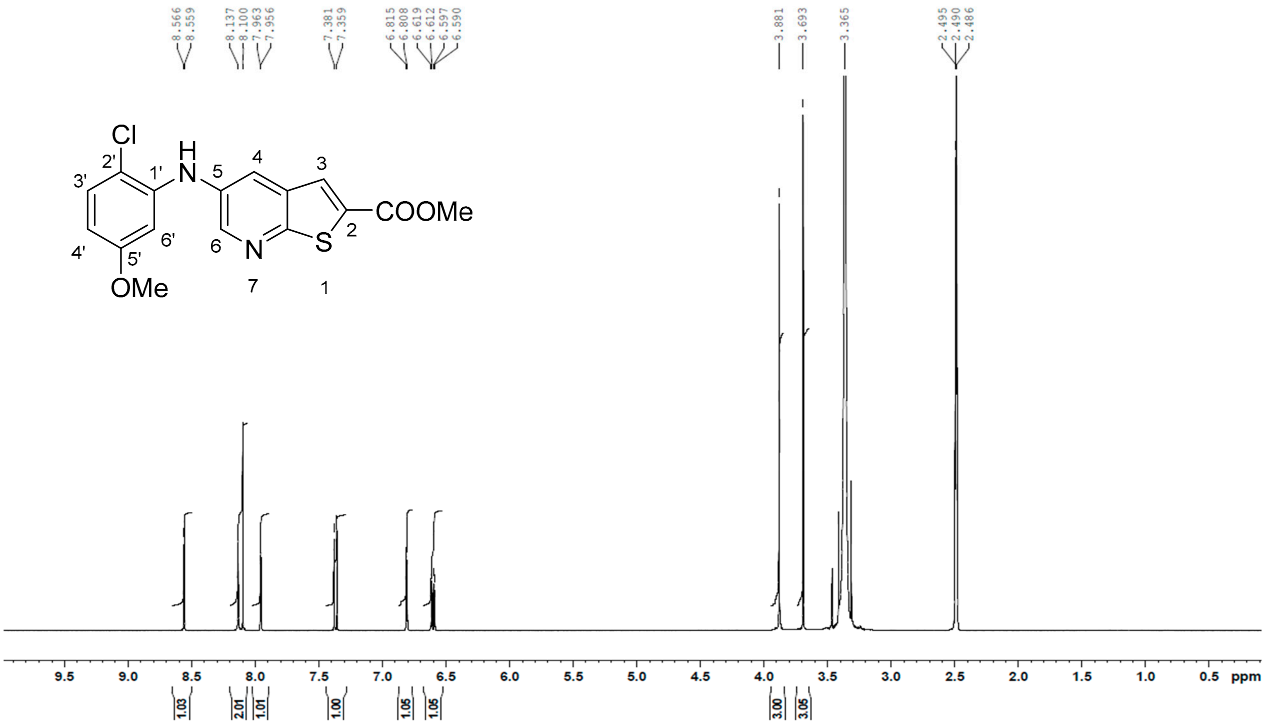

^{13}C NMR (DMSO-d_6, 100.6 MHz) of compound 2l

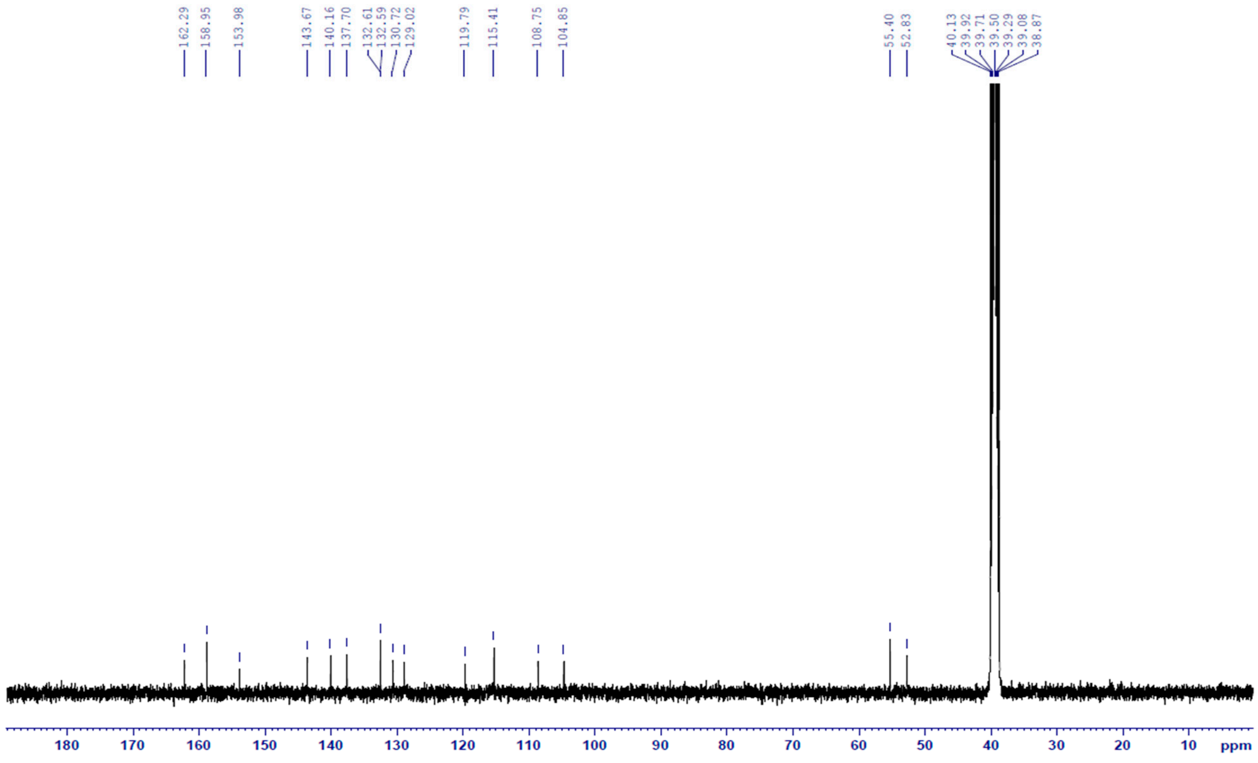

14.NMR spectra of compound 2m

^{1}H NMR (DMSO- *d*6, 400 MHz) of compound 2m

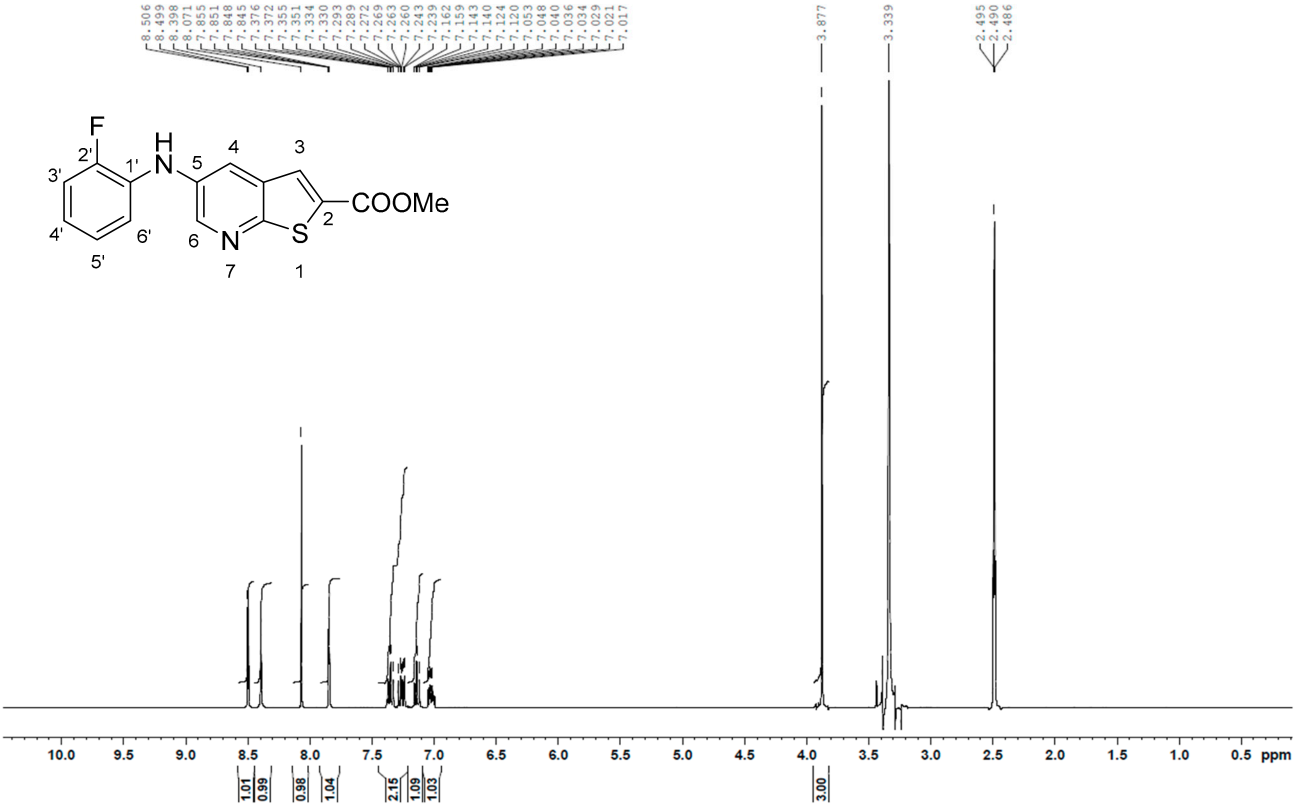

^{13}C NMR (DMSO-*d*6, 100.6 MHz) of compound 2m

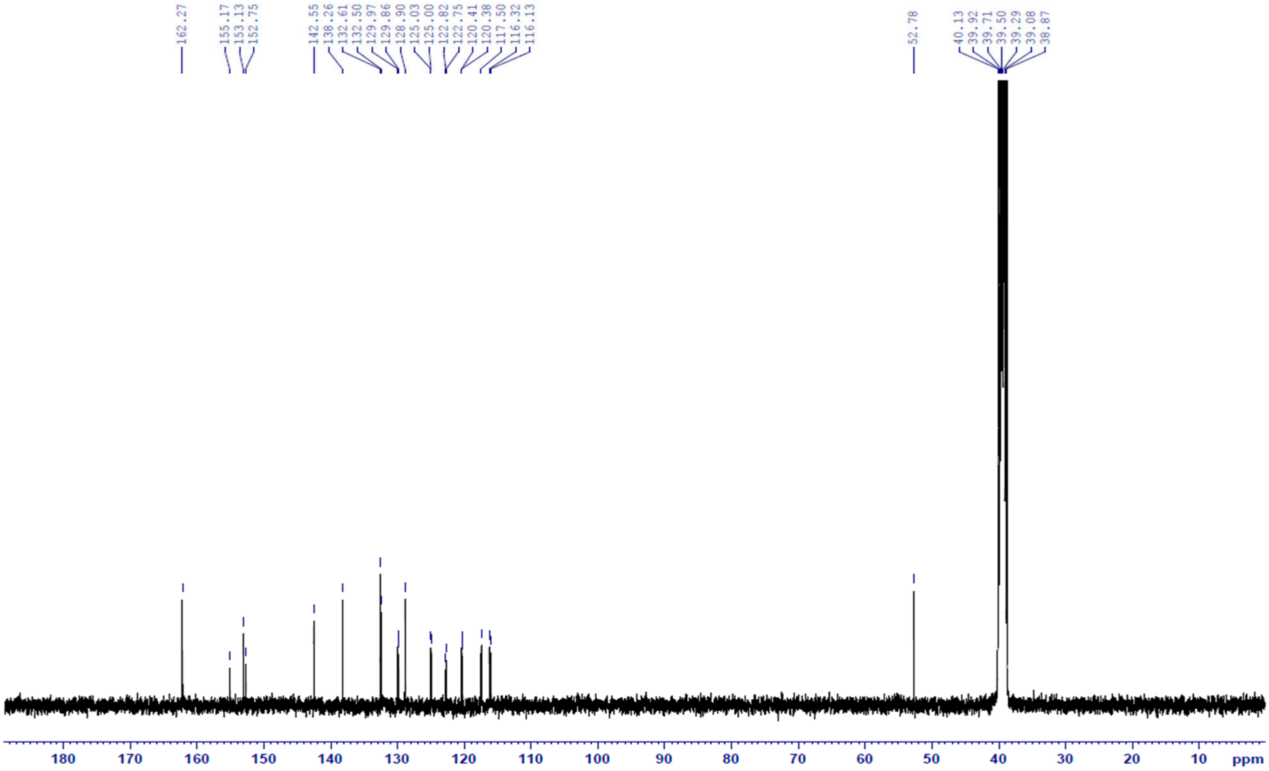

15.NMR spectra of compound 2n

^{1}H NMR (DMSO- d_6, 400 MHz) of compound 2n

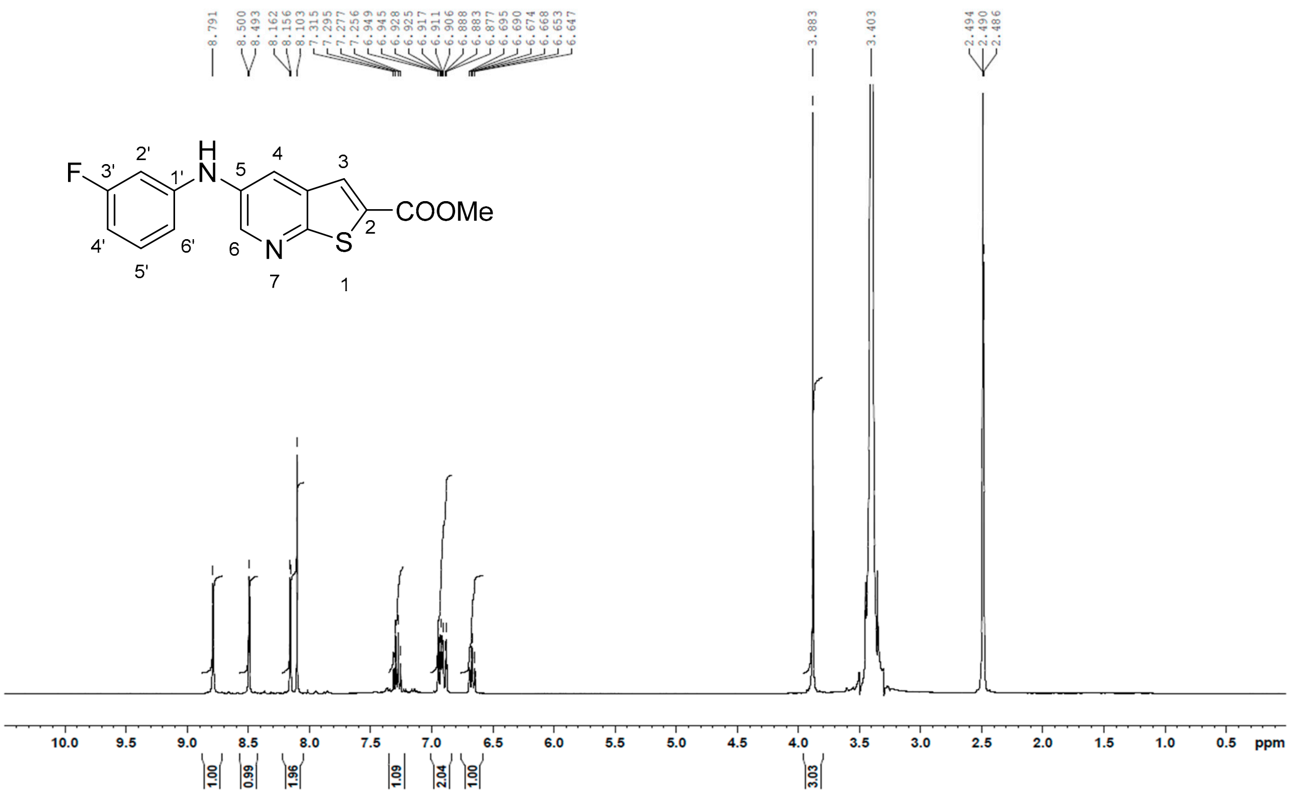

^{13}C NMR (DMSO-d_6, 100.6 MHz) of compound 2n

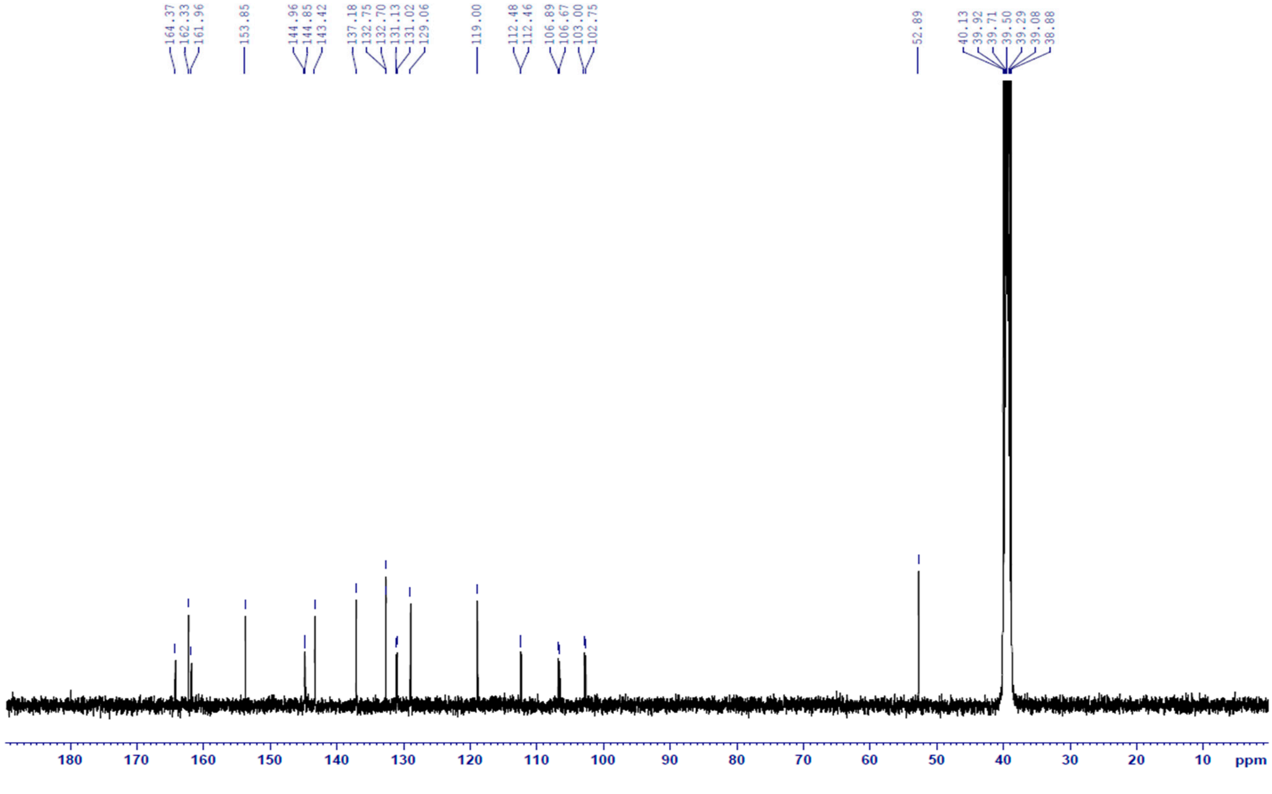

16.NMR spectra of compound 2o

^{1}H NMR (DMSO- *d*6, 400 MHz) of compound 2o

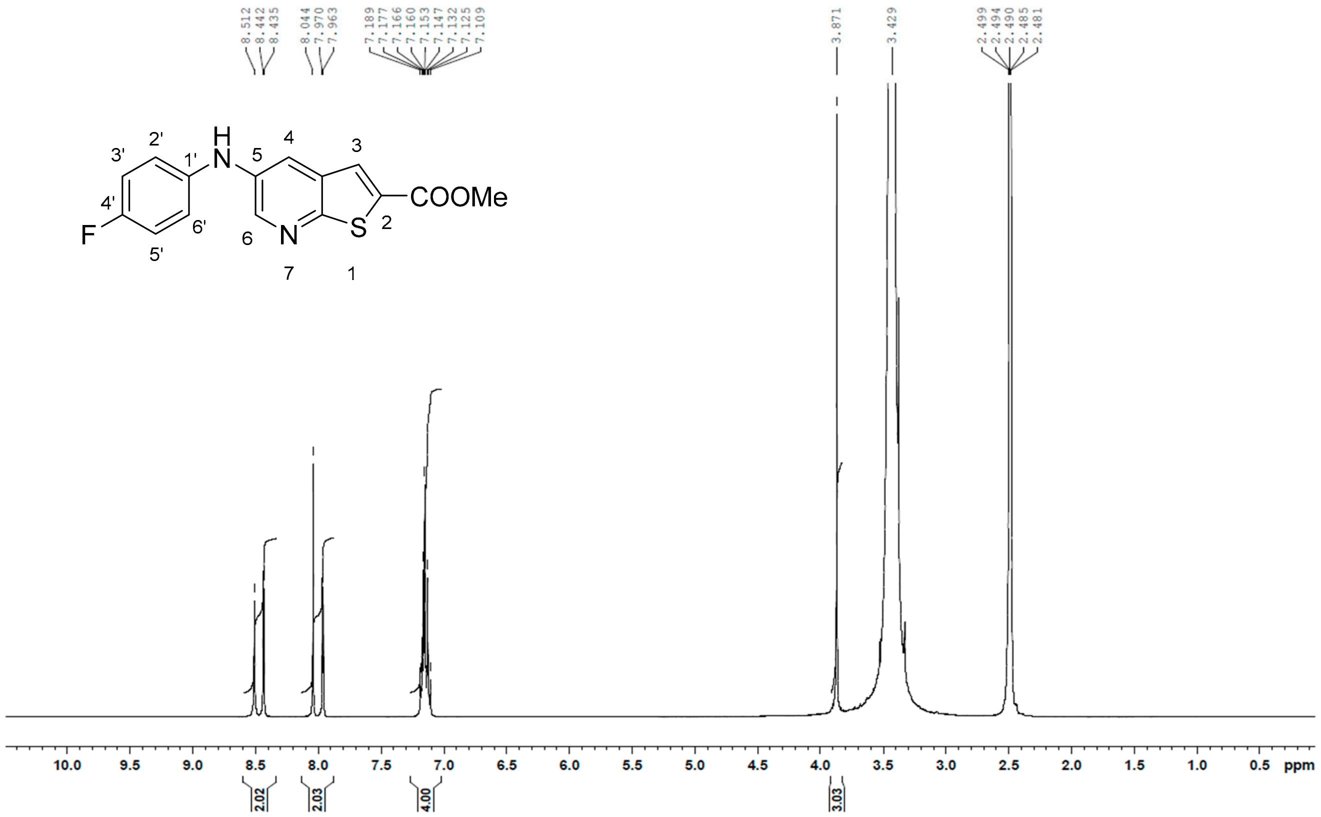

^{13}C NMR (DMSO-*d*6, 100.6 MHz) of compound 2o

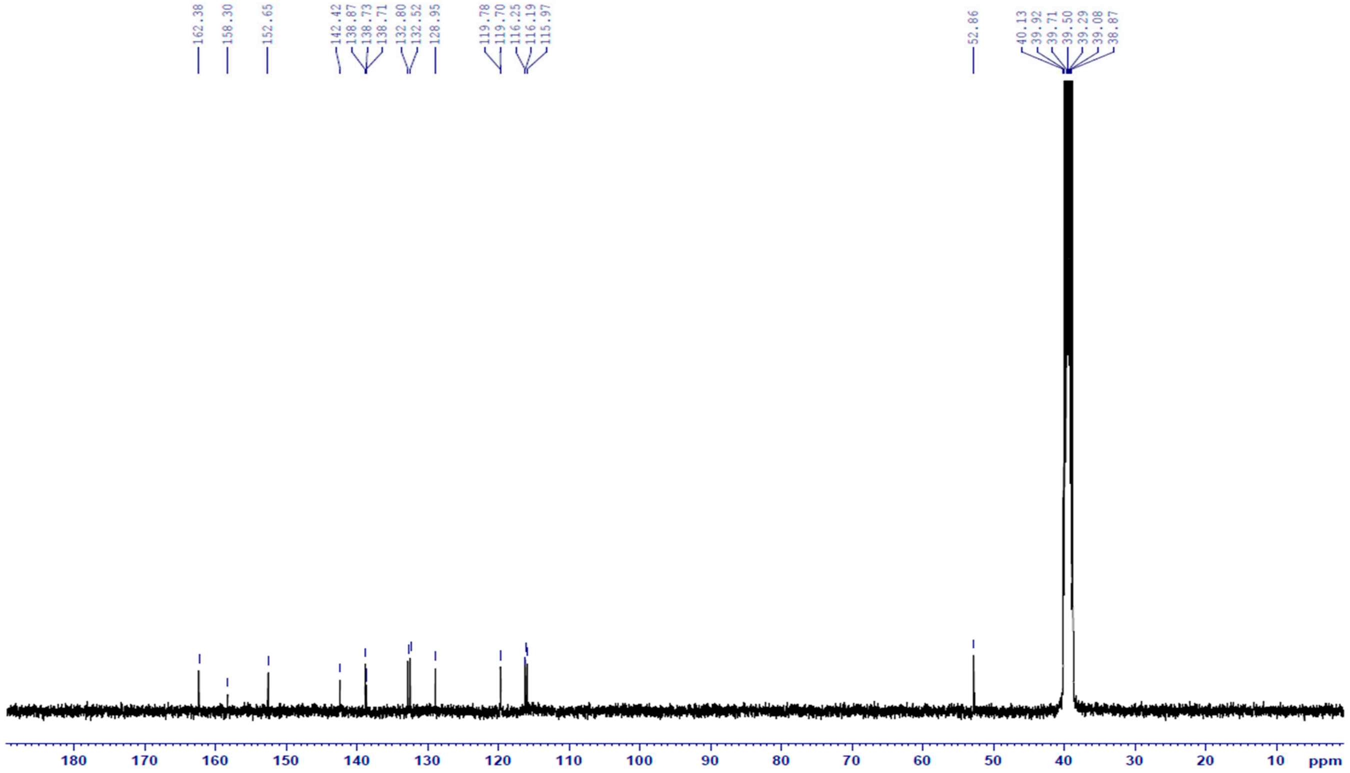

HMBC spectrum of compound 2o

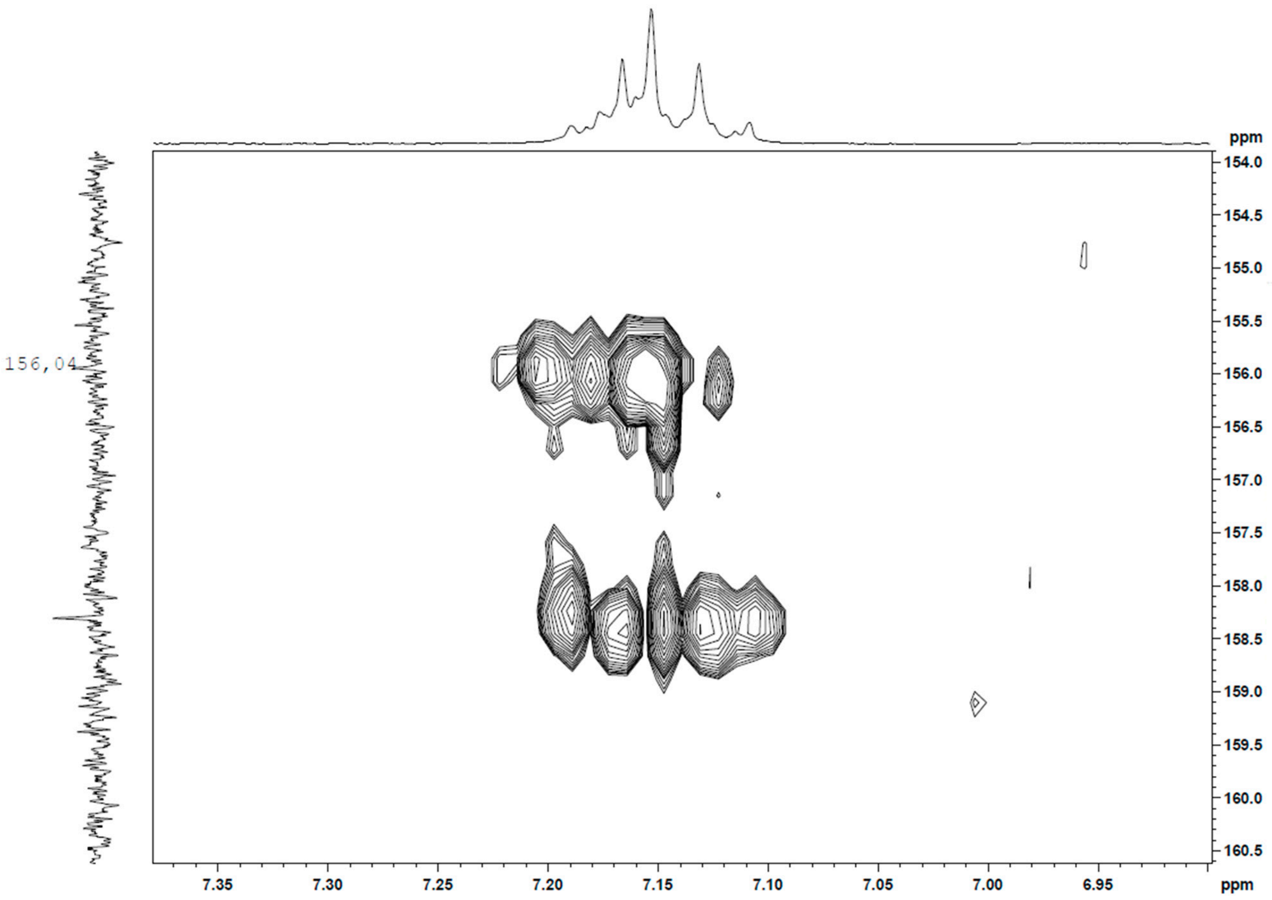

17.NMR spectra of compound 2p

^{1}H NMR (DMSO- d_6, 400 MHz) of compound 2p

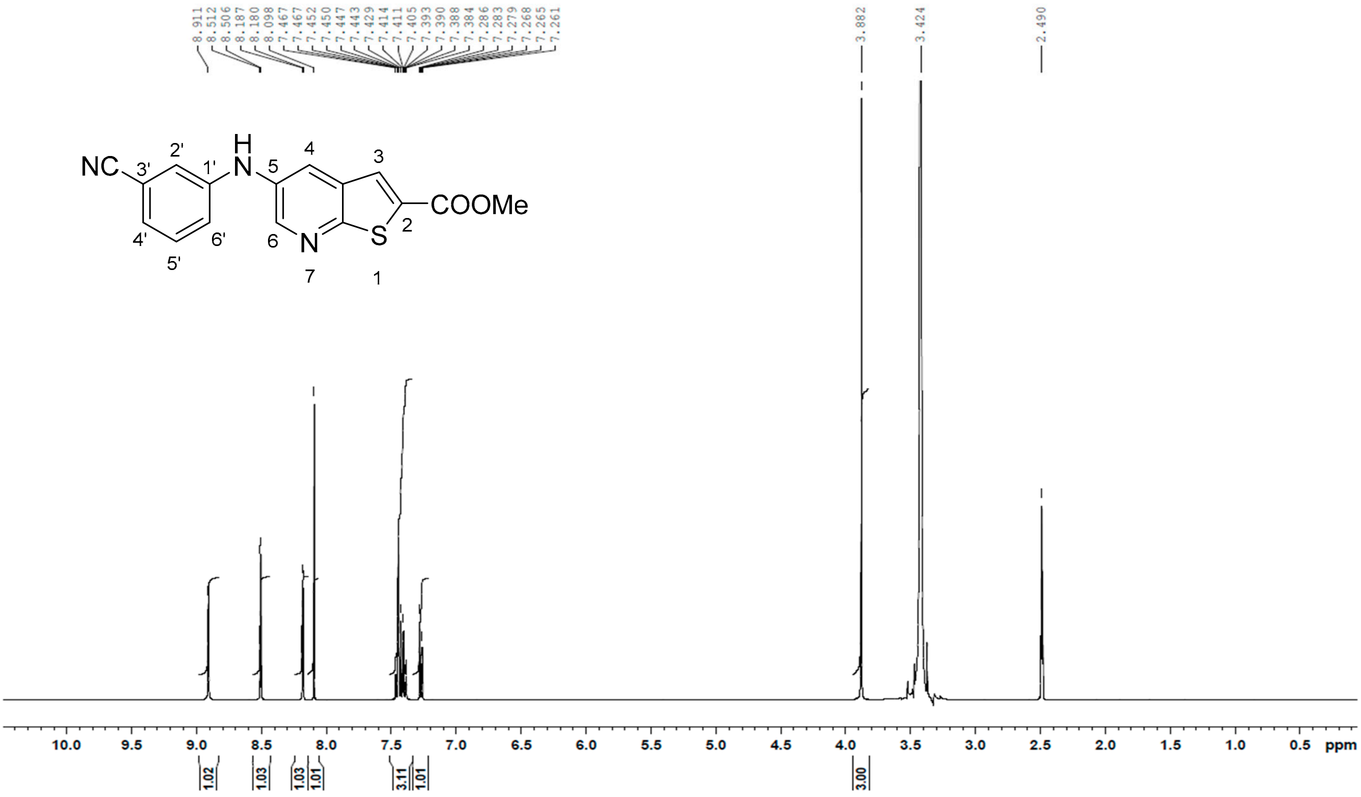

^{13}C NMR (DMSO-d_6, 100.6 MHz) of compound 2p

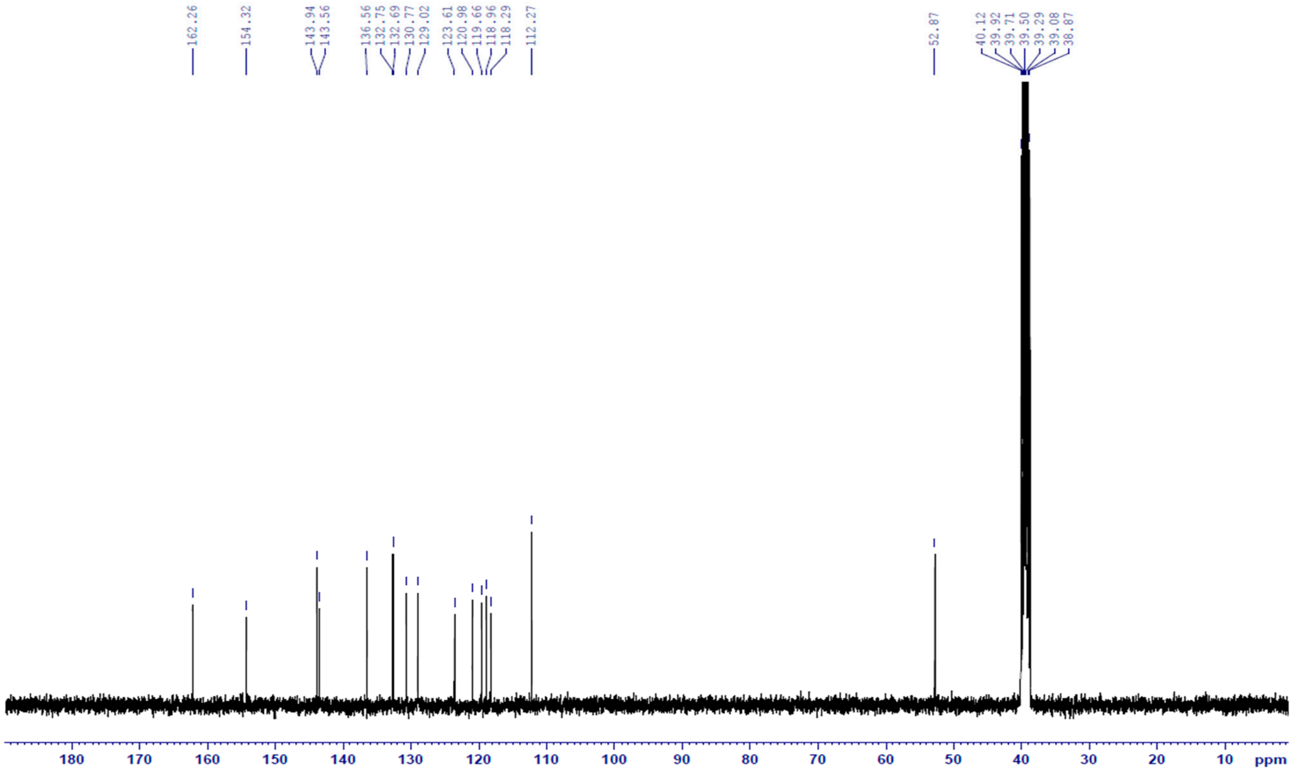

18.NMR spectra of compound 2q

^{1}H NMR (DMSO- d_6, 400 MHz) of compound 2q

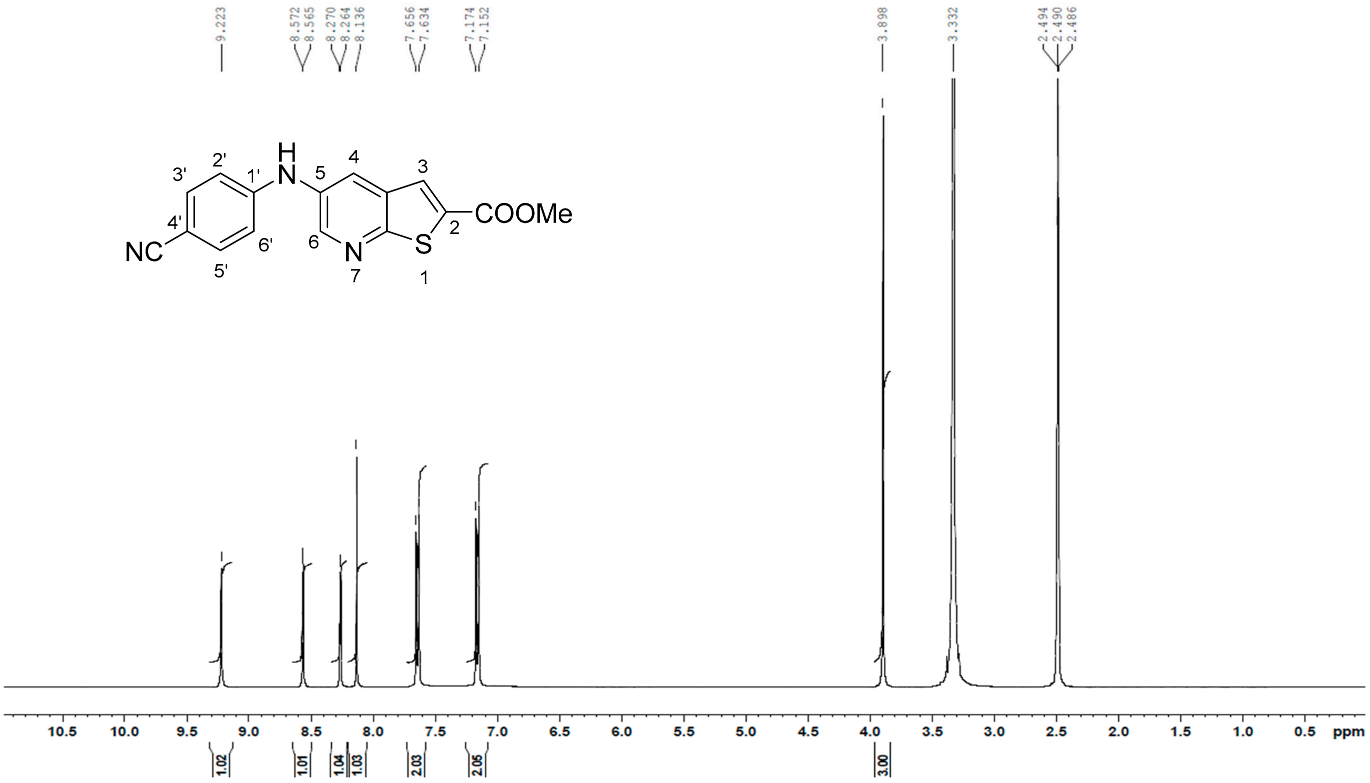

^{13}C NMR (DMSO-d_6, 100.6 MHz) of compound 2q

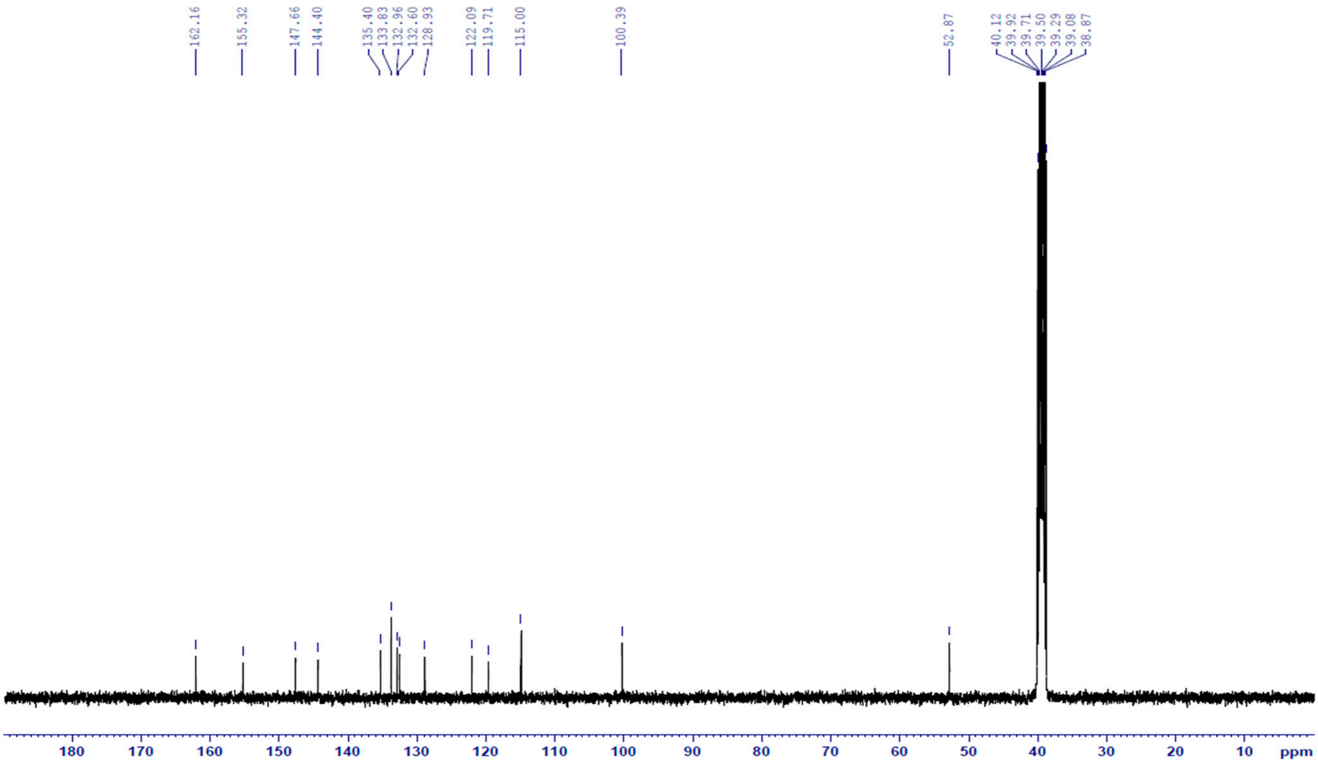

19.NMR spectra of compound 2r

^{1}H NMR (DMSO- d_6, 400 MHz) of compound 2r

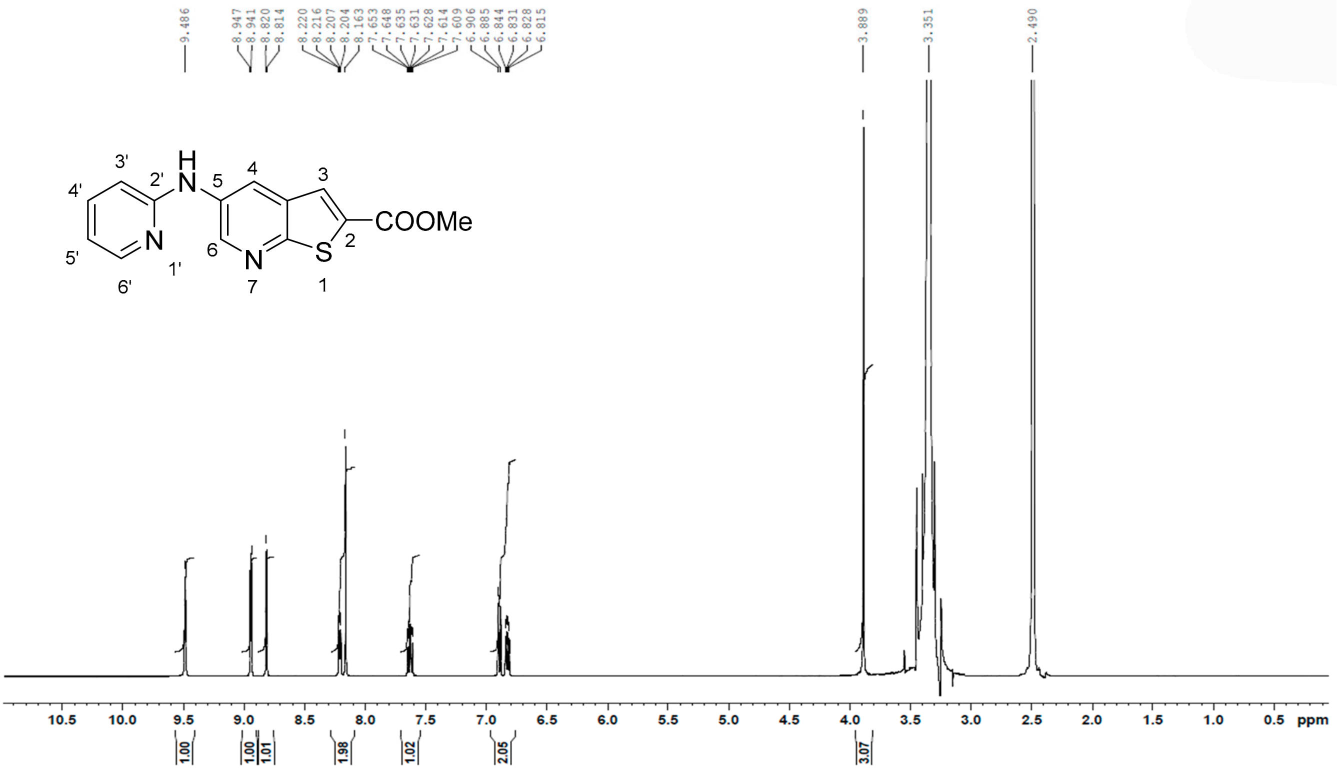

^{13}C NMR (DMSO-d_6, 100.6 MHz) of compound 2r

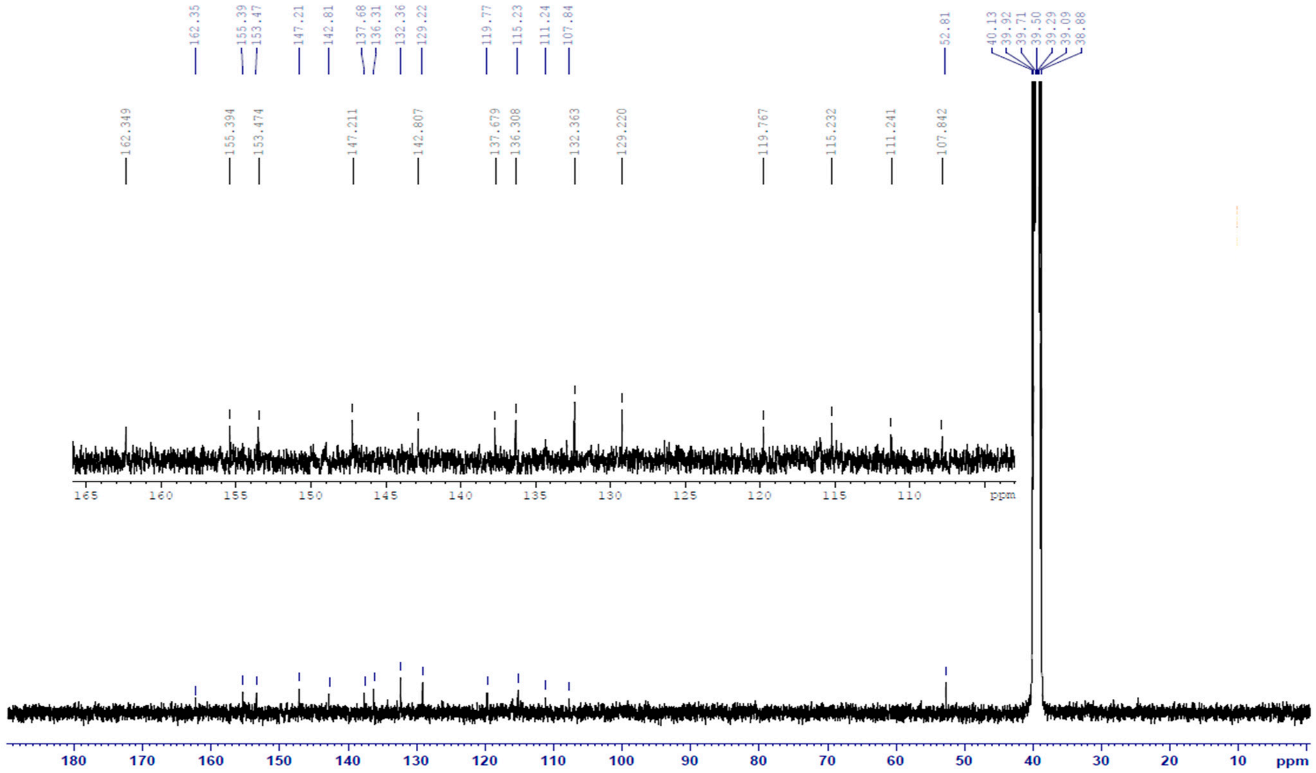

20.NMR spectra of compound 2s

^{1}H NMR (DMSO- *d*6, 400 MHz) of compound 2s

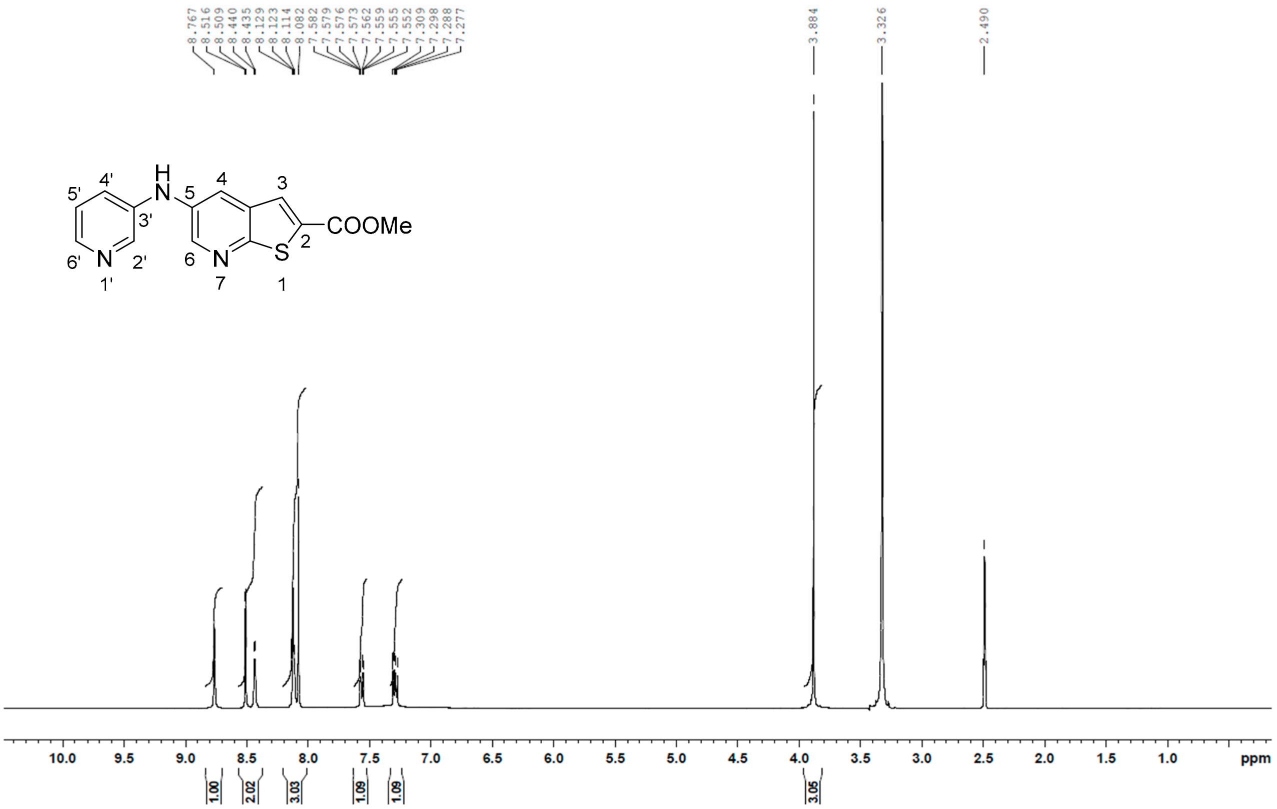

^{13}C NMR (DMSO-*d*6, 100.6 MHz) of compound 2s

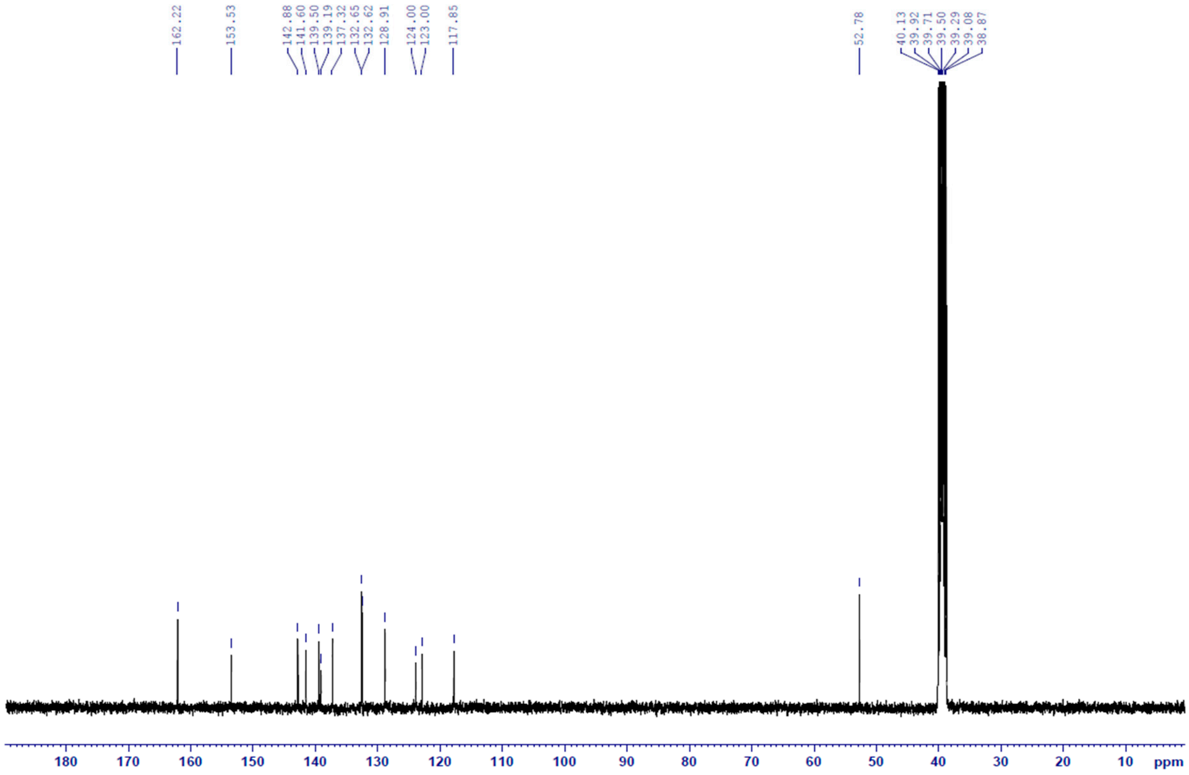

21.NMR spectra of compound 2t

^{1}H NMR (DMSO- *d6*, 400 MHz) of compound 2t

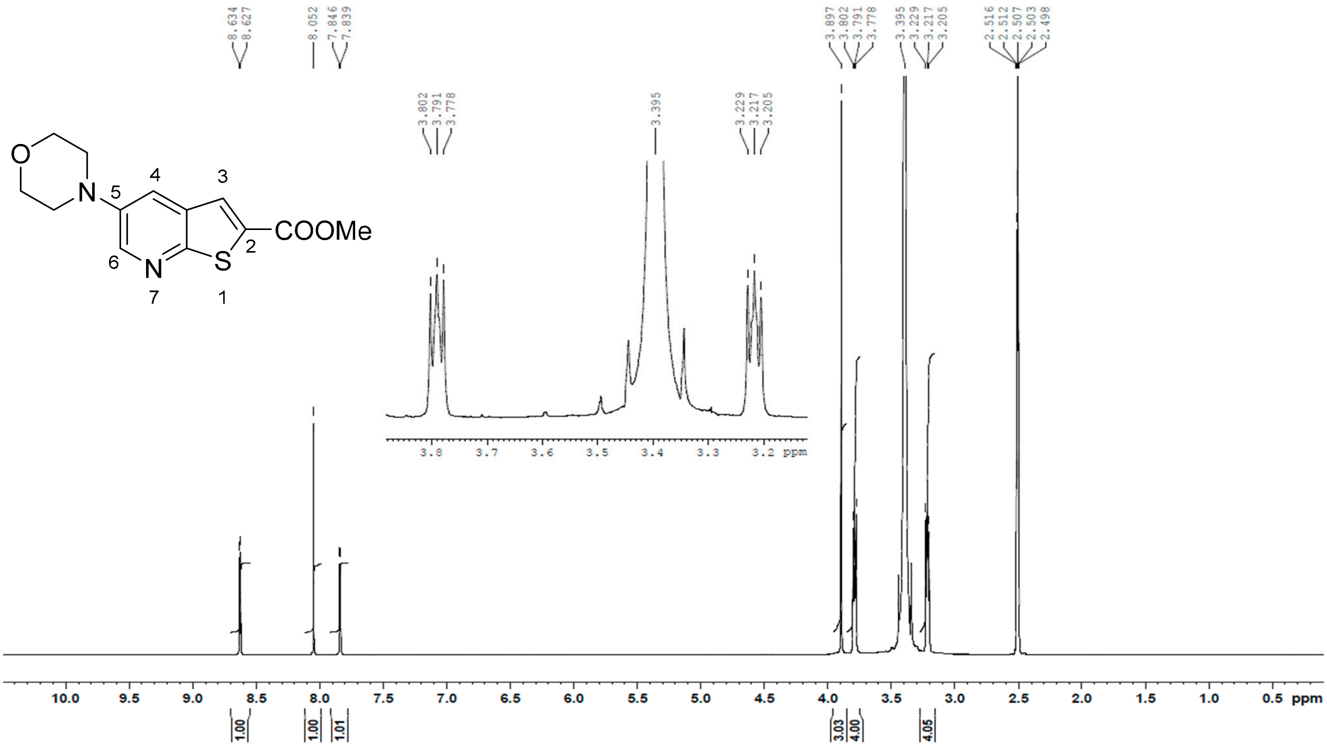

^{13}C NMR (DMSO-*d6*, 100.6 MHz) of compound 2t

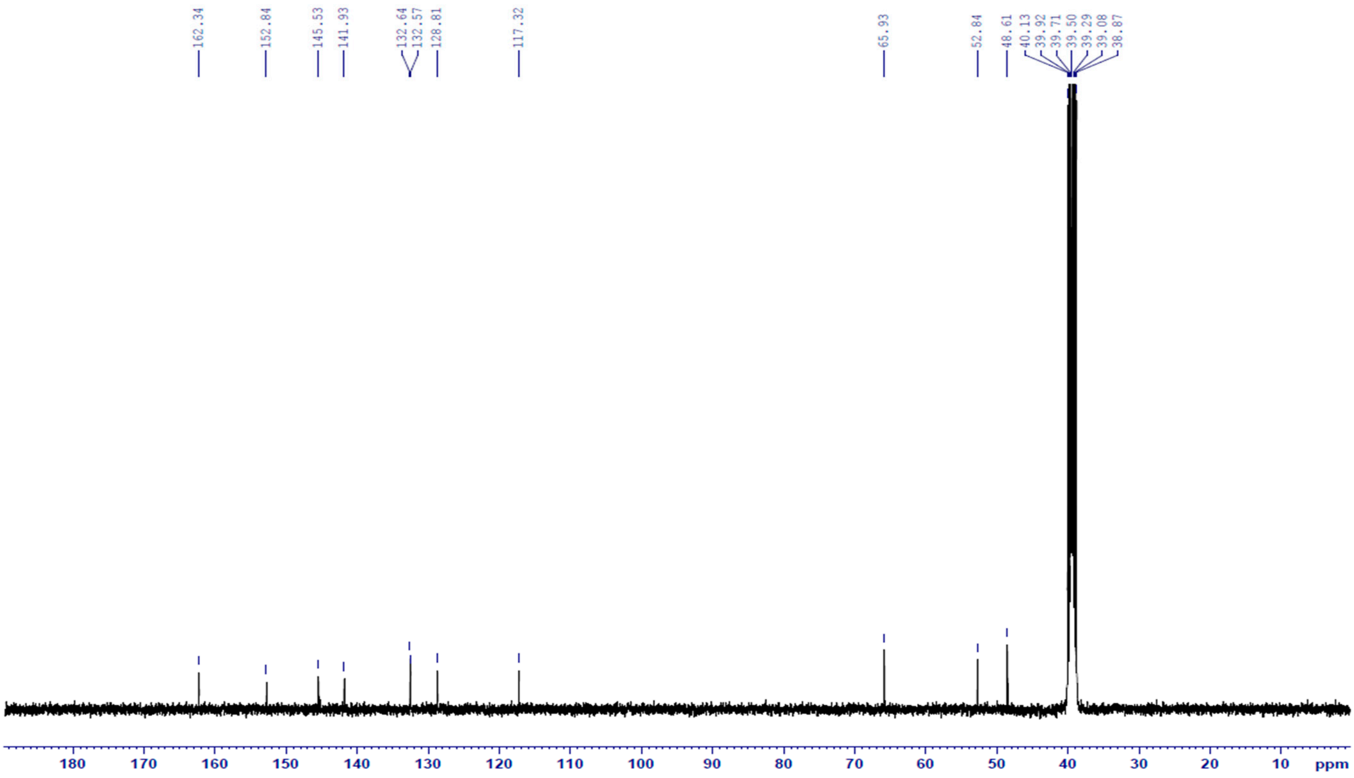

22.NMR spectra of compound 2u

^{1}H NMR (DMSO- d_6, 400 MHz) of compound 2u

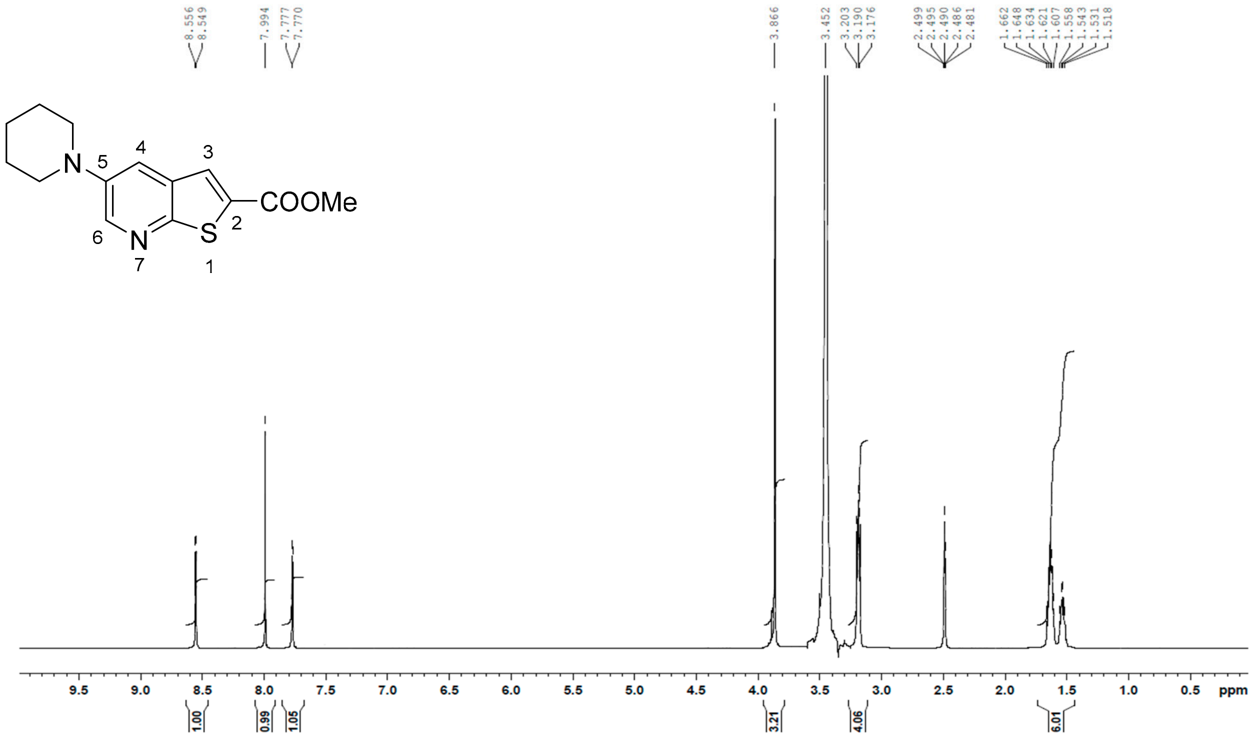

^{13}C NMR (DMSO-d_6, 100.6 MHz) of compound 2u

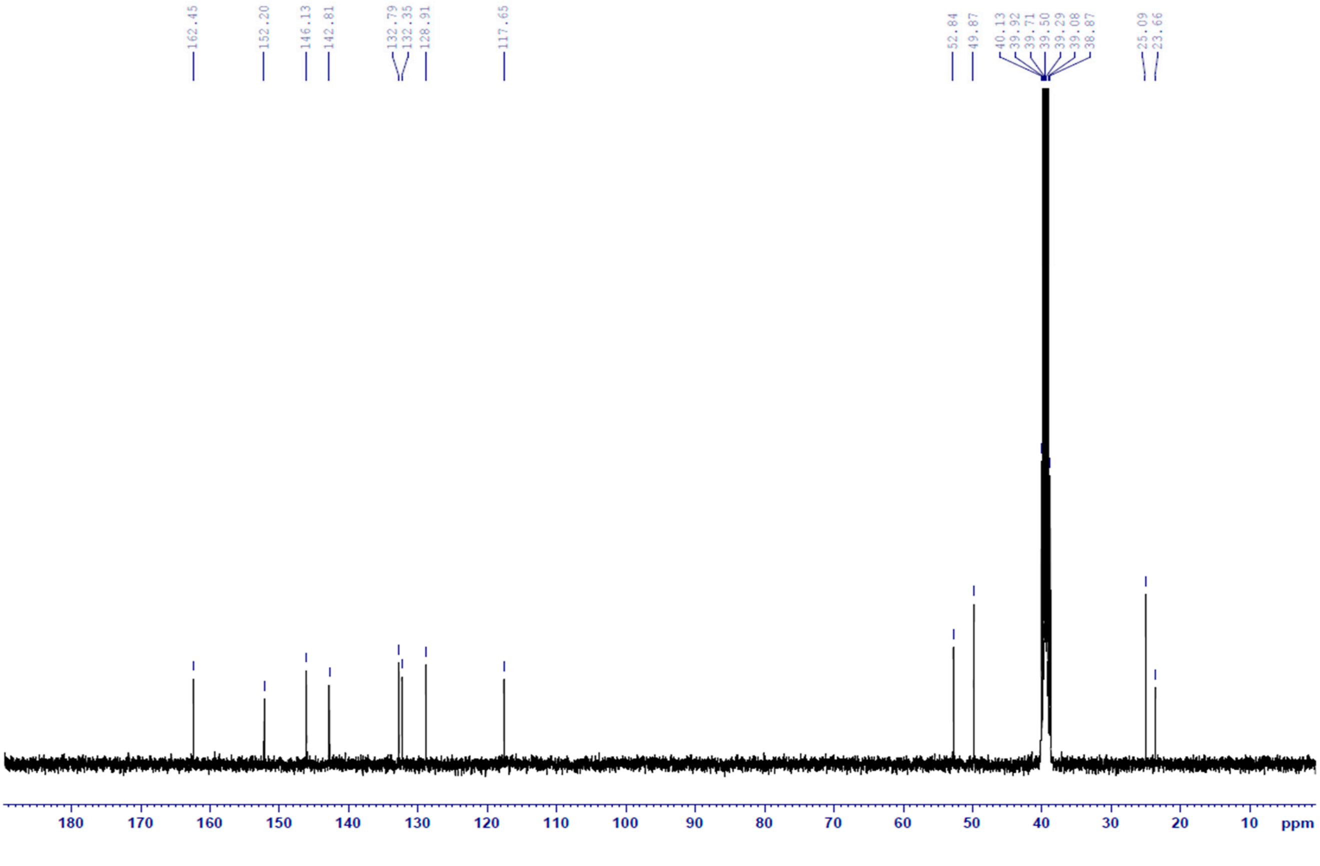

Table S1. Antiparasitic activity (single dose 20 μM) against *T. brucei* and *L. infantum* promastigotes for compounds **2a**-**2u**

Compounds	*T. brucei*	*L. infantum* promastigotes
	% of activity ± ST.DEV (single dose 20 μM)	% of activity ± ST.DEV (single dose 20 μM)
2a	47±8.0	N.A.
2b	50±26	N.A.
2c	91±5.0	N.A.
2d	61±12	N.A.
2e	54±7.0	95±4.0
2f	96±3.0	N.A.
2g	99±1.0	42±5.0
2h	35±1.0	70±26
2i	53±3.0	80±20
2j	77±32	86±12
2k	76±23	80±18
2l	54±30	N.A.
2m	N.A.	N.A.
2n	58±1.0	39±42
2o	N.A.	N.A.
2p	N.A.	73±9.0
2q	N.A.	70±12
2r	N.A.	N.A.
2s	N.A.	N.A.
2t	N.A.	N.A.
2u	N.A.	N.A.

N.A. = No Activity.
